# G6PD Deficiency Prevalence and Estimates of Affected Populations in Malaria Endemic Countries: A Geostatistical Model-Based Map

**DOI:** 10.1371/journal.pmed.1001339

**Published:** 2012-11-13

**Authors:** Rosalind E. Howes, Frédéric B. Piel, Anand P. Patil, Oscar A. Nyangiri, Peter W. Gething, Mewahyu Dewi, Mariana M. Hogg, Katherine E. Battle, Carmencita D. Padilla, J. Kevin Baird, Simon I. Hay

**Affiliations:** 1 Spatial Ecology and Epidemiology Group, Department of Zoology, University of Oxford, Oxford, United Kingdom; 2 Kenya Medical Research Institute/Wellcome Trust Programme, Centre for Geographic Medicine Research-Coast, Kilifi District Hospital, Kilifi, Kenya; 3 Eijkman-Oxford Clinical Research Unit, Jakarta, Indonesia; 4 Department of Pediatrics, College of Medicine, University of the Philippines Manila, Manila, Philippines; 5 Newborn Screening Reference Center, National Institutes of Health (Philippines), Ermita, Manila, Philippines; 6 Centre for Tropical Medicine, Nuffield Department of Clinical Medicine, University of Oxford, Oxford, United Kingdom; Menzies School of Health Research, Australia

## Abstract

**Background:**

Primaquine is a key drug for malaria elimination. In addition to being the only drug active against the dormant relapsing forms of *Plasmodium vivax*, primaquine is the sole effective treatment of infectious *P. falciparum* gametocytes, and may interrupt transmission and help contain the spread of artemisinin resistance. However, primaquine can trigger haemolysis in patients with a deficiency in glucose-6-phosphate dehydrogenase (G6PDd). Poor information is available about the distribution of individuals at risk of primaquine-induced haemolysis. We present a continuous evidence-based prevalence map of G6PDd and estimates of affected populations, together with a national index of relative haemolytic risk.

**Methods and Findings:**

Representative community surveys of phenotypic G6PDd prevalence were identified for 1,734 spatially unique sites. These surveys formed the evidence-base for a Bayesian geostatistical model adapted to the gene's X-linked inheritance, which predicted a G6PDd allele frequency map across malaria endemic countries (MECs) and generated population-weighted estimates of affected populations. Highest median prevalence (peaking at 32.5%) was predicted across sub-Saharan Africa and the Arabian Peninsula. Although G6PDd prevalence was generally lower across central and southeast Asia, rarely exceeding 20%, the majority of G6PDd individuals (67.5% median estimate) were from Asian countries. We estimated a G6PDd allele frequency of 8.0% (interquartile range: 7.4–8.8) across MECs, and 5.3% (4.4–6.7) within malaria-eliminating countries. The reliability of the map is contingent on the underlying data informing the model; population heterogeneity can only be represented by the available surveys, and important weaknesses exist in the map across data-sparse regions. Uncertainty metrics are used to quantify some aspects of these limitations in the map. Finally, we assembled a database of G6PDd variant occurrences to inform a national-level index of relative G6PDd haemolytic risk. Asian countries, where variants were most severe, had the highest relative risks from G6PDd.

**Conclusions:**

G6PDd is widespread and spatially heterogeneous across most MECs where primaquine would be valuable for malaria control and elimination. The maps and population estimates presented here reflect potential risk of primaquine-associated harm. In the absence of non-toxic alternatives to primaquine, these results represent additional evidence to help inform safe use of this valuable, yet dangerous, component of the malaria-elimination toolkit.

*Please see later in the article for the Editors' Summary*

## Abstract 2

Rosalind Howes and colleagues present a map of glucose-6-phosphate dehydrogenase deficiency prevalence and severity. Individuals with the deficiency are at risk of mild to severe hemolysis when taking the antimalarial primaquine.

## Editors' Summary

**Background:**

Malaria is a parasitic infection that is transmitted to people through the bites of infected mosquitoes. Of the four parasites that cause malaria, *Plasmodium falciparum* is the most deadly and *P. vivax* is the commonest and most widely distributed. Malaria parasites have a complex life cycle. Infected mosquitoes inject “sporozoites” into people, a form of the parasite that replicates inside human liver cells. After a few days, the liver cells release “merozoites,” which invade red blood cells where they replicate rapidly before bursting out and infecting other red blood cells. This increase in the parasitic burden causes malaria's characteristic fever and can cause organ damage and death. Infected red blood cells also release “gametocytes,” which infect mosquitoes when they take a blood meal. In the mosquito, gametocytes multiply and develop into sporozoites, thus completing the parasite's life cycle. Malaria can be prevented by controlling the mosquitoes that spread the parasite and by avoiding mosquito bites by sleeping under insecticide-treated bed nets. Treatment with effective antimalarial drugs also decreases malaria transmission.

**Why Was This Study Done?:**

The Global Malaria Action Plan aims to reduce malaria deaths to zero by 2015 and to eradicate malaria in the long-term through its progressive elimination in malaria-endemic countries (countries where malaria is always present). Primaquine is a key drug for malaria elimination. It is the only treatment effective against the gametocytes that transmit malaria between people and mosquitoes and against *P. vivax* “hypnozoites,” which hibernate in the liver and cause malaria relapses. Unfortunately, primaquine induces mild to severe destruction of red blood cells (hemolysis) in people who have a deficiency in the enzyme glucose-6-phosphate dehydrogenase (G6PD). G6PD deficiency (G6PDd) is common in some ethnic groups but the global distribution of individuals at risk of primaquine-induced hemolysis is unknown and there is no practical field test for G6PDd. Consequently, it is hard to design and implement primaquine treatment practices that balance the benefits of malaria transmission reduction and relapse prevention against the risk of hemolysis. Here, the researchers use a geostatistical model to map the prevalence (frequency in a population) of G6PDd in malaria-endemic countries and to estimate how many people are affected in these countries. They also develop a national index of relative hemolytic risk.

**What Did the Researchers Do and Find?:**

The researchers fed data from community surveys of the prevalence of phenotypic G6PDd (reduced enzyme activity) for 1,734 sites (including 1,289 sites in malaria-endemic countries) into a geostatistical model originally developed to map global malaria endemicity. The model predicted that G6PDd is widespread across malaria-endemic regions, with the lowest prevalences in the Americas and the highest in tropical Africa and the Arabian Peninsula, but that most G6PDd individuals live in Asian countries. The predicted prevalence of G6PDd varied considerably over relatively short distances in many areas but, averaged across malaria-endemic countries it was 8%, which corresponds to about 350 million affected individuals; averaged across countries that are currently planning for malaria elimination, the prevalence was 5.3% (nearly 100 million affected individuals). Finally, the researchers used data on the geographical occurrence of G6PD variants classified according to their enzyme activity levels as mild or severe to derive an index of hemolytic risk from G6PDd for each malaria-endemic country. The greatest risk was in the Arabian Peninsula and west Asia where the predicted prevalence of G6PDd and the occurrence of severe G6PD variants were both high.

**What Do These Findings Mean?:**

These findings suggest that G6PDd is widespread and spatially heterogeneous across most of the malaria-endemic countries where primaquine would be valuable for malaria control and elimination. The accuracy of these findings is limited, however, by the assumptions made in the geostatistical model, by the accuracy of the data fed into the model, and by the lack of data for some malaria-endemic countries. Moreover, there is considerable uncertainty associated with the proposed index of hemolysis risk because it is based on phenotypic G6PDd enzyme activity classifications, which is presumed, but not widely demonstrated, to be a surrogate marker for hemolysis. Nevertheless, these findings pave the way for further data collection and for the refinement of G6PDd maps that, in the absence of non-toxic alternatives to primaquine, will guide the design of safe primaquine regimens for the elimination of malaria.

**Additional Information:**

Please access these Web sites via the online version of this summary at http://dx.doi.org/10.1371/journal.pmed.1001339.

- Information is available from the World Health Organization on malaria; its 2011 World Malaria Report provides details of the current global malaria situation (some information is available in several languages)
- The US Centers for Disease Control and Prevention provide information on malaria (in English and Spanish), including a selection of personal stories about malaria
- Information is available from the Roll Back Malaria Partnership on the global control of malaria and on the Global Malaria Action Plan
- Information on the global mapping of malaria is available at the Malaria Atlas Project website where G6PD deficiency prevalence maps, population estimates and the data used in this study can also be accessed
- Information about G6PD deficiency for affected families can be found on KidsHealth from the Nemous Children's Health System and the G6PD Deficiency Association website
- MedlinePlus provides links to additional information on malaria; the MedlinePlus Encyclopedia provides information about G6PD deficiency (in English and Spanish)

## Introduction

A third of malaria endemic countries (MECs, 35/99) now plan for malaria elimination [1]–[3]. This strategy is very distinct from routine malaria control, requiring not only the reduction of clinical burden, but complete depletion of the parasite reservoir by attacking the gametocytes responsible for transmission and killing the silent hypnozoites that may otherwise relapse [4]–[8]. Primaquine, an 8-aminoquinoline, is the only drug available for each of those therapeutic compartments [9],[10], and is thus key to any elimination strategy [11]. However, this drug can also be dangerously toxic to individuals with a genetic deficiency in glucose-6-phosphate dehydrogenase (G6PDd), usually a clinically silent condition [12]. Tafenoquine (GSK) is a new drug in phase IIb/III clinical trials intended to replace primaquine, but is likely to retain haemolytic toxicity in G6PDd patients [13]. No alternative non-toxic drugs with these unique modes of action are currently close to clinical trials [8].

The 2010 World Health Organization (WHO) guidelines for uncomplicated *P. falciparum* malaria treatment recommend a single dose of primaquine alongside artemisinin-based combination therapy (ACT) to prevent parasite transmission, particularly as a component of pre-elimination or elimination programmes [14],[15] and as part of artemisinin resistance containment programmes [16]. This gametocytocidal therapy has been shown to be effective in low endemicity settings in combination with an ACT [17], and in theory could significantly reduce transmission levels [18]. However, evidence for a derived community benefit is poor and a recent Cochrane review finds little support for these WHO treatment guidelines [19]. Transmission may be sustained by sub-microscopic gametocyte levels [7],[20], meaning that effective blocking of community transmission may require wider drug administration beyond symptomatic cases [4],[21].

Key to sustaining progress towards malaria elimination is the prevention of parasite reintroduction from the relapsing malarias *P. vivax* and *P. ovale* [8]. This therapeutic target is complicated by the absence of diagnostic testing for liver-stage parasites [22], and recent studies suggest high prevalence of hypnozoites, even in areas considered to have relatively low transmission intensity [23]. Although recommended dosages vary regionally, 14-d regimens of primaquine (either 15 or 30 mg daily adult doses) are advised for successful hypnozoite treatment [14]. The key impediment to attacking hypnozoite reservoirs among endemic populations in this way is the risk of potential harm from primaquine [24].

Primaquine can cause mild to severe haemolysis in G6PDd patients. The mechanism of primaquine-induced haemolysis is not fully understood. Reduced G6PD enzyme activity levels are likely to create a redox equilibrium within red blood cells that favours oxidised species of highly reactive primaquine metabolites. In one hypothesis, the 5-hydroxyprimaquine metabolite would be dominated by its oxidised quinoneimine species in G6PDd red blood cells, which may then react with the haem moiety of haemoglobin and cause its displacement to the lipid bilayer of red blood cells [25]. The resulting acute intravascular haemolysis may be mild and self-limiting, or very severe and threaten life [26],[27]. Freely circulating haemoglobin may cause the most severe clinical symptoms, such as renal failure [28]. There is currently no practical point-of-care field test for G6PDd [29], leaving most primaquine treatment decisions blind to haemolytic risk. There is a difficult ethical balance for weighing the benefits of transmission reduction and relapse prevention against poorly defined haemolytic risks [24].

Understanding the distribution and prevalence of this genetic risk factor in any given area may substantially inform risk and thus better equip policy makers and practitioners alike in designing and implementing primaquine treatment practices. We respond here to demands from the malaria community for a prevalence map of this genetic condition [22],[30]. Existing published maps of G6PDd have important limitations. They either present average frequency data summarised to national levels thereby masking sub-national variation [31],[32] and enabling mapping only for countries from where surveys were identified, leaving gaps in the maps [33]; or use broad categorical classes to present basic data extrapolation [34]. None exclude potentially skewed or unrepresentative survey samples (such as malaria patients), none consider prevalence in females, none have a framework for assessing statistical uncertainty, and none have mechanisms for incorporating G6PDd spatial heterogeneity into population affected estimates.

In addition to the public health importance of G6PDd in the context of malaria elimination, the clinical burden of this genetic condition includes a range of haematological conditions, including neonatal jaundice and acute haemolytic anaemia in adults triggered by a range of foods, infections, and other drugs [26],[35]. Across the Asia-Pacific region, risk of neonatal complications due to G6PDd already justifies significant investment through inclusion in neonatal screening programmes in Malaysia, the Philippines, Taiwan, and Hong Kong [35].

In this study, we compile data from available sources of G6PDd prevalence surveys, and use these as the evidence-base to inform a Bayesian geostatistical model specifically adapted to the gene's X-chromosome inheritance mechanism. This model generates spatially continuous G6PDd prevalence predictions, and allows quantification of prediction uncertainty. The model predictions are then matched with high-resolution population data to estimate numbers of deficient individuals within MECs, accounting for the predicted sub-national heterogeneity in deficiency rates. Finally, we assemble a database of G6PDd variant occurrences and propose here an index for how the prevalence map could be used to stratify haemolytic risk at the national level.

## Methods

**Figure 1:**
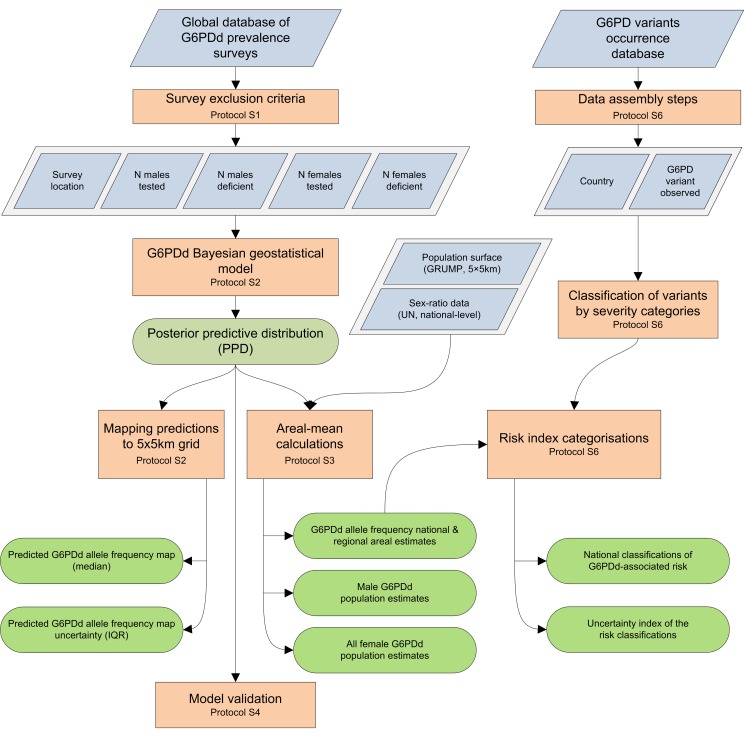
Schematic overview of the procedures and model outputs. Blue diamonds describe input data. Orange boxes denote data selection methods and analytical models. Green rods indicate model outputs.

This study's methodological objectives involved the assembly of representative community G6PDd prevalence surveys and the development of a Bayesian geostatistical model used to derive (i) maps of G6PDd prevalence within MECs, (ii) sex-specific estimates of the populations affected by this deficiency, and (iii) associated uncertainty metrics. These results were then combined with information on the distribution of the underlying G6PDd variants to generate an index for stratifying haemolytic risk from G6PDd. Each of these aspects is discussed briefly here, and in more detail in Protocols S1, S2, S3, S4, S5, S6. A schematic overview of the methodology is given in Figure 1.

### Prevalence Survey Database Assembly and Inclusion Criteria

A literature search of online bibliographic databases was conducted to identify published community surveys of G6PDd. Existing databases published by Singh et al. in 1973 [36], Mourant et al. in 1976 [37], Livingstone in 1985 [38], and Nkhoma et al. in 2009 [33] were reviewed for any further sources. Direct contact with national screening programmes and researchers in the field was also undertaken to identify additional unpublished data. All identified surveys were reviewed for suitability for informing the G6PDd prevalence mapping analysis (Protocol S1).

Inclusion criteria were applied to ensure: (i) community representativeness: all potentially biased samples were excluded (e.g., any patient groups including malaria patients, ethnically selected samples, and family-based studies); (ii) gender representativeness: only surveys reporting sex-specific raw data were included; (iii) spatial representativeness: only surveys that could be mapped with relatively confined extents (≤3,867 km^2^) were included to ensure that sub-national variation could be represented [39],[40]; (iv) clinically significant deficiency: only phenotypic diagnoses were considered. Because of the narrow range of primers usually used in molecular investigations, DNA-based diagnoses were excluded as they are susceptible to underestimating deficiency rates (Protocol S1) [24]. This study focused on G6PDd prevalence within MECs (corresponding to 99 countries, as defined in Protocol S1.5), with a particular focus on countries eliminating malaria (35 countries), but imposed no spatial restrictions to the dataset in order to make maximal use of existing information, particularly around the edges of the MEC limits.

The WHO uses mild and severe categorisations for G6PDd [31], with different treatment recommendations for each in relation to primaquine regimens [14]. Only through specific individual level G6PD testing can these be differentiated. The community level G6PD deficiency map presented here represents the prevalence of all clinically significant enzyme deficiency, as would be diagnosed by the common phenotypic diagnostic tests. Additional resolution into the severity of the deficiency is derived from the G6PDd variant database described below.

### The Model

A Bayesian geostatistical framework [41]–[46] was adopted to model the global prevalence of G6PDd. This framework used the evidence-base of surveys to generate predictions for G6PDd frequencies across the MECs, together with quantified uncertainty estimates for the predictions. This framework, developed for mapping the prevalence of a range of inherited blood disorders [40],[47],[48] was adapted to the X-linked inheritance mechanism of the G6PD gene [12]. Unlike females who have two copies, males inherit only a single copy of the G6PD gene, thus frequencies of deficiency in males correspond to the population-level allele frequency. Assuming populations to be at Hardy-Weinberg equilibrium [49],[50], squaring the deficiency allele frequency (*q*) gives an estimate of the expected prevalence of homozygous females (*q* ^2^). Phenotypic expression of female heterozygous (2*q*(1−*q*)) deficiency ranges across a spectrum of enzyme activity levels. Expression is variable due to irregular Lyonization rates [51] and inconsistent cut-off points of phenotypic diagnostic methods (Protocols S1, S2, and S5). Thus only a proportion of heterozygotes are diagnosed as phenotypically “deficient” [51],[52]. As no clear genotype-phenotype relationship could be identified from the observed survey data (Protocol S5), the model was given the flexibility to determine this relationship empirically, directly from the input data. The deviance of expected genetic heterozygotes from observed phenotypic deficiency cases (*h*) varied between surveys; *h* was modelled as a spatial variable, with values learned from the data, but not modelled as a spatially structured variable. The deviance value represents both the proportion of heterozygotes diagnosed as phenotypically normal, as well as actual deviance from expected Hardy-Weinberg equilibrium due to factors such as selection, consanguinity, migration, or small population sizes.

The model framework is thus *p*(*d*) = *q*+*q* ^2^+2*q*(1−*q*)*h*; where *p*(*d*) is the probability of an individual being phenotypically deficient, and *q* is the allele frequency for deficiency. From this equation, frequencies of hemizygotes (males, *q*), homozygotes (females, *q* ^2^), and all deficient females (homozygotes and phenotypically deficient heterozygotes: *q* ^2^+2*a*(1−*q*)*h* could be estimated. The model was fitted to the data and 1 million Markov chain Monte Carlo (MCMC) iterations [53] were used to generate full posterior predictive distributions (PPDs). The PPDs are summarised by the median value of the predictions and mapped continuously at 5×5 km resolution. Prediction uncertainty was quantified as the interquartile range (IQR) of the PPD. The model and its implementation are fully described in Protocol S2.

To validate the model predictions, an independent model iteration was implemented with a 95% subset of the dataset, allowing comparison of the predicted frequencies with observed frequencies from the 5% hold-out data. The hold-out data sample was selected to preferentially include spatially isolated data points, so as to ensure that the full prediction surface was included in the validation. Moreover, isolated areas are harder to make predictions for, and are therefore a conservative assessment of model reliability. Further details about validation methodology and derived statistics are given in Protocol S3.

### Estimating Populations Affected

To quantify the prevalence of G6PDd across national and regional populations, areal estimates (regional aggregates that account for uncertainty) [47] were calculated by relating the model predictions to high resolution population density data from the Global Rural Urban Mapping Project (GRUMP) *beta* version, adjusted to United Nations (UN) population estimates for the year 2010 [41],[54]. The areal-prediction model [47] was implemented to repeatedly sample G6PDd PPDs from selected locations, weighted according to population density, at a 5×5 km resolution. So, for each area of interest, the model generated an areal frequency PPD adjusted to the population density distribution across the area of interest. Multiplying the resulting aggregated G6PDd frequencies from the areal PPDs by UN 2010 national level population data adjusted for national-level sex ratio [55] gave estimates of the population numbers affected by each phenotype. To account for the stochasticity of the sampling, this process was repeated ten times for the national estimates, and five times for aggregated regional estimates (because of computational constraints) in order to calculate the Monte Carlo standard error associated with the estimates. This process is fully described in Protocol S4.

### Stratifying National G6PDd Severity

In order to stratify the potential haemolytic risk associated with G6PDd, a simple index was developed that incorporated both the national prevalence of the trait and the severity of the local genetic variants.

Predicted national prevalence was stratified into three categories (≤1%, >1–10%, and >10%). Stratifying the severity of the local forms of G6PDd was more involved. A second online literature review was conducted to assemble all reports of genetic and biochemical variants, using the same search methods as for assembling the prevalence data. All occurrences of named G6PDd variants were abstracted into a database and mapped to the country where they had been observed. Variants were then grouped according to their severity: the only severity classification widely applied to all variants is that proposed by Yoshida et al. [56], and endorsed by the WHO [31], which classifies variants according to their residual enzyme activity levels, their polymorphic/sporadic occurrence in populations, and the severity of their clinical symptoms (Protocol S6). Limitations to this classification system are reviewed in the Discussion. Only variants of class II (residual enzyme activity <10%) and class III (10%–60%) were relevant to this study. A score based on the relative composition of variants from these classes was assigned to each country to represent the relative proportions of class II and III variants: a proxy indicator of the severity of local variants. If no data were available from a country, a conservative approach was followed which took the highest score (most severe) from any neighbouring country.

The prevalence and variant severity scores were then multiplied to give a stratified measure of the relative haemolytic risk of G6PDd in each country. A similar uncertainty index was determined on the basis of the uncertainty in the prevalence estimates, and the availability and heterogeneity of variant data in each country. The variant data, risk scoring tables, and uncertainty estimates are presented in more detail in Protocol S6.

## Results

### The Prevalence Survey Database

Literature searches were conducted to collate all available reports of representative community G6PDd prevalence. A total of 17,272 G6PD abstracts were identified from online bibliographic databases, together with 472 potential data sources found in existing G6PDd databases [33],[36]–[38] and unpublished reports. Following careful review, 1,601 abstracts were considered suitable for our study and their full texts were reviewed for data. The Filipino Newborn Screening Reference Center (National Institutes of Health, Philippines) also contributed their universal screening results since 2004 to this study, adding 636 spatially unique locations to the database.

**Figure 2:**
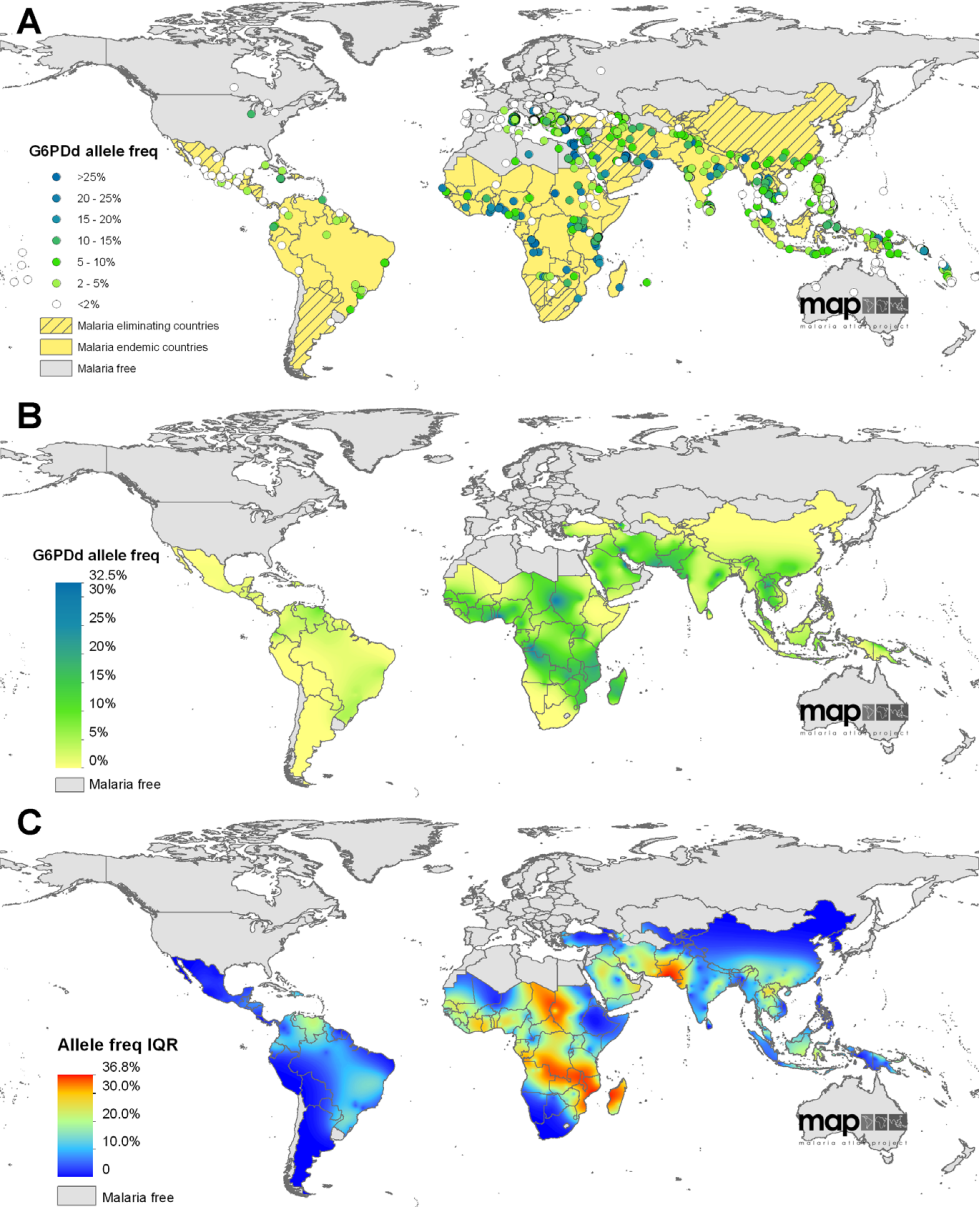
The global distribution of G6PDd. (A) shows the global assembly of G6PDd community surveys included in the model dataset; data points are coloured according to the reported prevalence of deficiency in males (*n* = 1,720). Background map colour indicates the national malaria status (malaria free/malaria endemic/malaria eliminating). (B) is the median predicted allele frequency map of G6PDd. (C) presents the associated prediction uncertainty metrics (IQR); highest uncertainty is shown in red and indicates where predictions are least precise.

The total number of surveys identified that met the inclusion criteria was 1,734 globally, with 74% from MECs (*n* = 1,289) (Figure 2A). Surveys were unevenly distributed, some areas having been examined in micro-mapping studies (such as Sri Lanka) and universal screening (Philippines) while large extents of other areas remain unstudied (e.g., extensive parts of Indonesia, Madagascar, and central Africa). Within the MECs, 85% of surveys (*n* = 1,101) were from 23 Asian countries; 10% of surveys (*n* = 132) represented 23 African countries; data from only nine countries in the Americas were identified, corresponding to 4% of surveys (*n* = 56). Male data were reported from 99% of the surveys, while 62% presented female data. Overall numbers of individuals sampled were 2.4 million males and 2.0 million females.

The database is described in more detail in Protocols S1 and S5, with additional discussion about the influence of diagnostic methodology on test outcome in males and females. Female diagnosis is known to depend on numerous factors; however, in the absence of any standardised or established mathematical relationships for modelling the genotype-phenotype association in females, we decided to use the input dataset as the evidence-base, and the mapping model was given the freedom to determine this spatially variable relationship according to the raw data (Protocols S1, S2, S5).

### G6PDd Prevalence Predictions: Overview

**Figure 3:**
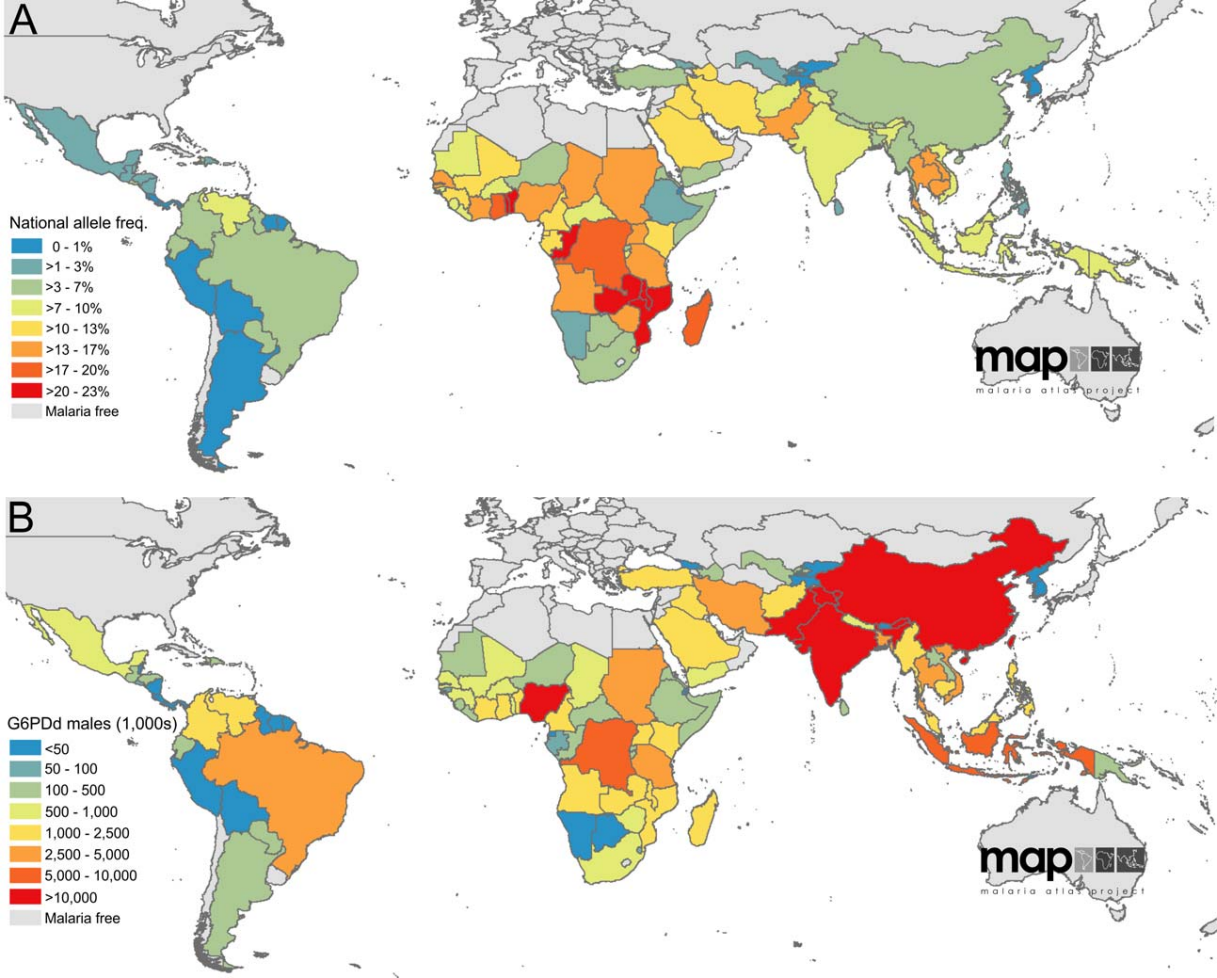
Population-weighted areal estimates of national G6PDd prevalence predictions. (A) summarises national-level allele frequencies, while (B) displays national-level population estimates of G6PDd males. Values are in thousands.

The survey database formed the evidence-base for the geostatistical model, which predicted both the spatially continuous map of G6PDd allele frequency (Figure 2) and the estimates of G6PDd populations (Figure 3); all model predictions are summarised with median values [53]. Model outputs indicated G6PDd to be widespread across malarious regions, with lowest frequencies in the Americas and highest in tropical Africa; an overall allele frequency of 8.0% (IQR: 7.4–8.8) was predicted across all MECs (Table 1). High population density in Asia meant that the highest numbers of G6PDd individuals were predicted to be from this continent (Table S1). The database and resulting model outputs indicated heterogeneity in G6PDd prevalence, with considerable variation across relatively short geographical distances in many areas (Figure 2B). All model predictions must be considered in relation to their associated uncertainty metrics (IQR; Figure 2C, Tables 1, S1 and S2). Model uncertainty is greatest where data points are scarce (Figure 2A) or where available data indicates heterogeneity (Protocol S2). Limitations to the database and the weaknesses that these lead to in the predictions are considered in the Discussion.

**Table 1:** G6PDd allele frequency and G6PDd population estimates across malaria endemic countries (*n* = 99) and the subset of malaria eliminating countries (*n* = 35).

G6PDd Allele Frequency and Population Estimates	Median (SE)		Q25(SE)		Q75(SE)	
	MEC^a^	Eliminating^b^	MEC^a^	Eliminating^b^	MEC^a^	Eliminating^b^
Allele frequency	8.04%(0.02%)	5.30%(0.01%)	7.44%(0.02%)	4.43%(0.02%)	8.81%(0.03%)	6.68%(0.02%)
G6PDd males	220,130(669)	61,227(96)	203,729(597)	51,200(184)	241,114(847)	77,223(251)
G6PDd females (homozygotes only^c^)	17,115(n/a)	3,100(n/a)	(n/a)	(n/a)	(n/a)	(n/a)
G6PDd females (all females)	132,932(467)	35,205(71)	121,618(550)	28,862(96)	147,814(693)	45,608(144)

All figures are in thousands. Q25 and Q75 refer to the low and high limits of the IQR of the model predictions. Numbers in brackets represent the Monte Carlo standard error (SE) of the estimates; presented in the same units as the associated estimate. Full explanations are given in Protocol S4.

a Total regional male population: 2,736,515; Total regional female population: 2,644,975. Source: GRUMP-adjusted projected UN 2010 population estimates and sex-ratio data from UN World Population Prospects 2010 Revision.

b Total regional male population: 1,156,300; Total regional female population: 1,105,603. Source: GRUMP-adjusted projected UN 2010 population estimates and sex-ratio data from UN World Population Prospects 2010 Revision.

c Figures derived from the allele frequency estimates so do not have specific model-derived uncertainty metrics.

n/a, not available.

### G6PDd Allele Frequency Map

Large swathes of the American MECs were predicted to have median G6PDd frequencies ≤1% (40.8% land area), with G6PDd being virtually absent from northern Mexico, Costa Rica, Peru, Bolivia, and much of Argentina (Figure 2). Prevalence increased towards coastal regions, peaking in Venezuela where the majority of the continent's predictions of >5% were located. Model uncertainty was relatively low across most of the Americas (IQR: <5%), with the IQR increasing to 5%–10% across the Amazon region where data were extremely scarce, and peaking between 15%–20% across Venezuela.

At the continental level, G6PDd was most prevalent across sub-Saharan Africa: 65.9% of the land area was predicted to have median G6PDd prevalence ≥5%, and 37.5% a median prevalence ≥10%. Predictions ranged from <1% at the continental extremities (western Sahel, Horn of Africa, and southern Africa) to >20% in isolated pockets of Sudan, coastal west Africa, and around the mouth of the river Congo. These broad patterns were interspersed with some striking sub-national variation within countries with deficiency hotspots, including Nigeria (range: 2% [IQR: 1–6] to 31% [22]–[42]), Sudan (1% [0–2] to 29% [19]–[41]) and Democratic Republic of Congo (DRC) (4% [1]–[11] to 32% [23]–[41]). These areas were also associated with the highest levels of model uncertainty—a reflection of this sub-national heterogeneity and also of the scarcity of input data from these areas. Highest prediction uncertainty across the continent was found in Sudan, Chad, and central Africa between DRC and Madagascar.

The highest median predicted prevalence of G6PDd across the entire MEC region was 32.5% in the Eastern Province of Saudi Arabia (specifically, around the urbanised coastal areas of Al-Qatif and Ad-Dammam). More broadly, rates across this disparately populated peninsula as a whole were heterogeneous, for example, dropping to prevalence of 3% (IQR: 2–4) in the central Al-Kharj and Riyadh area of Saudi Arabia. Further east, predicted prevalence remained high into southern Pakistan.This region had the highest uncertainty of the entire map (IQR exceeding 30%). No surveys were available from the south of Pakistan, and the closest neighbouring surveys in southern Iran, Oman, and western India reported prevalence of >20%, contrasting data from northern Pakistan. Prediction uncertainty dropped across central and southeast Asia, and predicted prevalence remained largely <10%, with three notable G6PDd prevalence hotspots in the central and southeast Asia regions peaking to >20%: (i) among the tribal, endogamous groups of Orissa province in east India, (ii) a patch along the northern Lao/Thai border, and (iii) much of the Solomon Islands archipelago. Underlying the broadly smooth continental-level variation, some areas were predicted to have highly heterogeneous sub-national G6PDd prevalence. Across Lao People's Democratic Republic (PDR), for instance, frequencies were predicted to range from 1% (IQR: 0–2) to 23% (16–32); predictions in Indonesia were from 0% (0–1) to 15% (10–21) in Nusa Tenggara; in Papua New Guinea, frequencies ranged from 1% (0–2) along the southern coast to 15% (10–22) along the East Sepik northern coast (Figure 2B–2C).

### Validation Statistics

The predicted allele frequency surface was evaluated against a hold-out subset of the data selected with spatially declustered randomization that preferentially selected data sparse sites where model predictions would be inherently most difficult [41]. Differences between predicted and observed prevalence returned a mean error of 1.45% and a mean absolute error of 4.07%. These indicate a slight tendency of the model to overestimate prevalence, and relatively more substantial error in the magnitude of prediction precision. Full validation results are given in Protocol S3.

### G6PDd Prevalence Predictions: Population Affected Estimates

The second modelling process related the allele frequency predictions to population distribution, generating sex-specific aggregated estimates of G6PDd populations, weighted by population distribution across the spatial regions of interest: national, malaria endemic, and the subset of 35 MECs targeting malaria elimination. These population-weighted estimates were modelled separately from the mapping process, and used the full model predictions, not just the summary median allele frequency map (Figure 2B). As with the map, these areal predictions and their associated uncertainty were summarised with median and IQR values (Tables 1 and S1).

We estimated overall G6PDd allele frequency across MECs to be 8.0% (IQR: 7.4–8.8); using 2010 population data (Protocol S4), this corresponded to 220 million males (IQR: 203–241) and an estimated 133 million females (122–148), including 17 million homozygous females (assuming Hardy-Weinberg equilibrium). Across the subset of malaria eliminating countries (Figure 1), prevalence was lower, at 5.3% (4.4–6.7). Population estimates for 2010 across this subset of eliminating countries were 61 million G6PDd males (51–77) and an expected 35 million G6PDd females (29–46), including 3 million homozygous females.

National frequency estimates ranged from 0.1% in Cape Verde (IQR: 0.0–0.5) and the Democratic People's Republic of Korea (0.0–0.4) to 22.3% in the Solomon Islands (15.7–30.9), 22.5% in the Congo (17.3–29.6) and 23.0% in Benin (17.0–30.1). Reflecting the prevalence map, national allele frequency estimates were generally lowest in the Americas and highest in Africa (Figure 3A). Converting these national-level allele frequency estimates to G6PDd population numbers (G6PDd males; Figure 3B), however, shifts attention away from Africa towards the highly populous Asian countries, notably China and India where 41.3% of G6PDd males within MECs were predicted to be. Overall, the Americas contributed only 4.5% of the MEC G6PDd male population, sub-Saharan Africa 28.0%, and Asia an estimated 67.5%.

### Index of National G6PDd Severity

**Figure 4:**
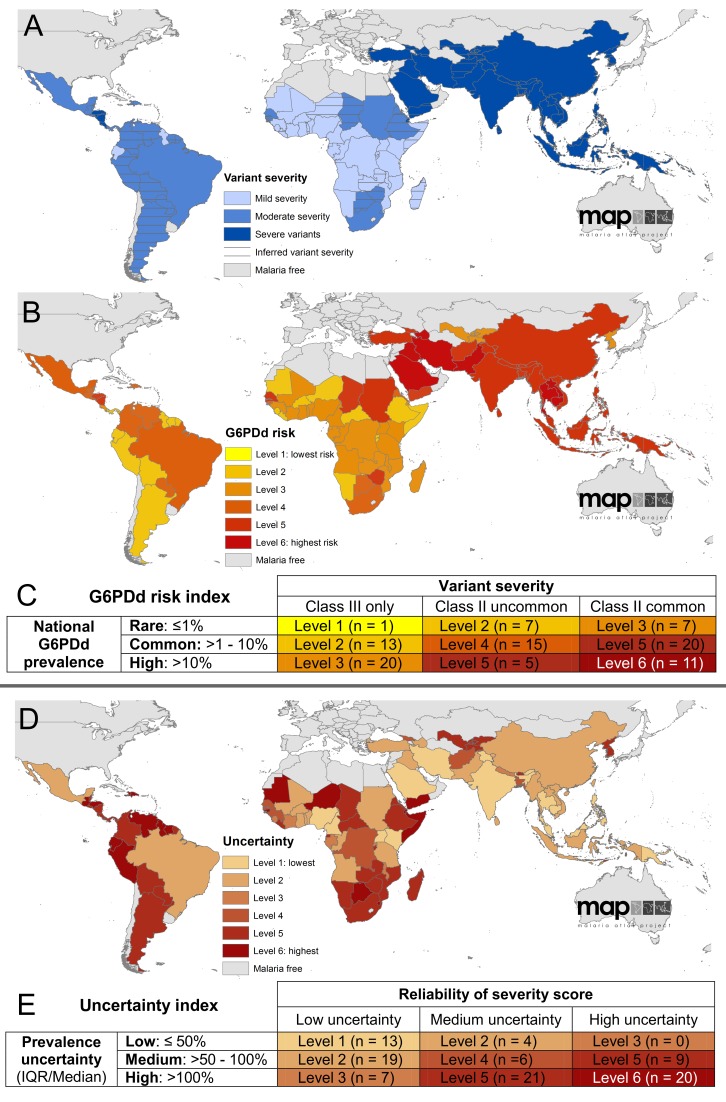
Index of severity risk from G6PDd. (A) shows the national score of variant severity, determined by the ratio of class II to class III variant occurrences reported from each country; (B) maps the risk index from G6PDd, accounting for both the severity of variants (A) and the overall prevalence of G6PDd (Figure 3A); the scoring matrix describing these scores is given in (C), specifying the different categories of risk determined by the scores of national-level prevalence of phenotypic deficiency (rows) multiplied by severity scores of the variants present (columns). (D) represents the uncertainty in the assembly of the risk index based on the prevalence scores (E rows) and in the assessment of variant severity (E columns). These uncertainties relate specifically to the analysis of these data into the risk index, and do not account for the underlying uncertainty in their interpretation in relation to haemolysis (see Discussion).

Data searches for reports of G6PDd variants identified 527 occurrences of class II variants and 405 class III variants from a total of 54 countries out of 99 MECs (Table S3). Occurrences of these data points were used to score the severity of the overall composition of variants in each country, with scores inferred from neighbouring countries in instances where no data points had been reported (Figure 4A). Once combined with a rank of G6PDd prevalence, an overall score of the severity of risk from G6PDd was derived for each country (Figures 4B–4C). A similar scoring was used to determine the relative confidence in the severity scores, shown in Figures 4D–4E. Further figures and the table of all variant occurrences by country are given inProtocol S6 and Table S3.

This index of risk is predicated on the current state of knowledge of G6PDd variant occurrence and the relationship between variants and haemolysis, as outlined in the Discussion. From the present dataset, we see strong regional patterns in the distribution of variants, with sub-Saharan Africa being predominantly ranked as having mildly severe variants (class III), predominantly A−, though some class II variants were reported from Sudan and South Africa, and Senegal and the Gambia in west Africa (Table S3). Relatively few data were available from the Americas, but these included a greater diversity of variants including a minority of class II variants. In contrast, variant reports were more heterogeneous across Asia, a majority of which were class II (most commonly Mediterranean, then Canton and Kaiping), though certain class III variants were also widely reported (Mahidol, then Chinese-5 and Gaohe being most frequently identified); the predominance of class II variants put the classification of all Asian countries as having severe variants.

Combining these variant severity scores with the scores of G6PDd prevalence gave an index of overall risk from G6PDd for each MEC. Greatest haemolytic risk from G6PDd was found in the Arabian Peninsula and across west Asia, where both prevalence and variant severity (dominated by the class II Mediterranean variant) were high. Across the Asian continent, risk remained high (level 5 of 6, increasing to level 6 in the Mekong region where prevalence was at its highest). In contrast, despite high prevalence, the low severity of the variants reported from sub-Saharan Africa resulted in the lowest risk categorizations from G6PDd globally, which was a moderate risk (mostly levels 2 to 3 of 6, though increasing to level 5 in countries where class II variants had been reported).

The uncertainty inherent in this synthesis is considerable; however, the index indicated that according to the metrics employed in this study, uncertainty ranked highest in many sub-Saharan countries and most countries in the Americas (where 19 of 21 countries had uncertainty ranked 5–6 out of 6). Further data from these regions would substantially improve reliability both of the modelled prevalence predictions, as well as of the variant severity categorisations, many of which had to be inferred from neighbouring countries.

The framework proposed here can be updated and refined as new data about variant occurrence and haemolytic risk become available.

## Discussion

G6PDd is widespread across malarious regions, where we estimated the deficiency to have an overall allele frequency of 8.0%. We have developed here an evidence-based, geostatistically modelled, and spatially continuous prevalence map of G6PDd, together with uncertainty metrics and population estimates of affected individuals. Although highest levels of G6PDd frequency are predicted in sub-Saharan Africa, high population density makes Asia the centre of weight of G6PD deficiency-burdened populations. We discuss our results first in relation to existing G6PDd maps, and then in their public health context in relation to the coincident severity of local variants. Important limitations to the maps and population estimates stem from weaknesses in the underlying database of surveys. These are also discussed, in relation to the difficulties of predicting deficiency in females, in assessing the robustness of the model predictions, and in overcoming the barriers to predicting the severity of primaquine-induced haemolysis.

### Comparison with Existing Maps and Population Estimates

Previous G6PDd maps have been published by the WHO G6PD Working Group in 1989 [31], Cavalli-Sforza et al. in 1994 [34], and more recently in 2009 by Nkhoma et al. [33]. Both the WHO and Nkhoma et al. maps present data averages at national levels, thus masking all sub-national variation and making direct comparisons with our continuous prevalence map difficult. Further, Nkhoma et al.'s map has many gaps for countries from where no data could be found. However, all maps show broadly similar patterns, with lowest frequencies in the Americas, highest rates predicted across the tropical belt of sub-Saharan Africa, and generally heterogeneous distributions across Asia ranging from virtually absent to relatively high. Comparison of the national-level, population-weighted allele frequency estimates generated here with the WHO categories showed no obvious trends, with estimates for 29% of MECs predicted higher here than by WHO, frequencies in 36% of countries being predicted lower than those predicted by WHO, and 35% having consistent values. Reasons for these disparities relate both to the criteria imposed on the survey evidence-base (with both WHO and Nkhoma et al. including surveys that were excluded from this current study for risk of bias or lack of spatial specificity, corresponding to 108 and 17 surveys, respectively) and the statistical methods involved (accounting for the sample size and spatial distribution of data points, and relating G6PDd prevalence to spatial patterns of population density). The new map also has the benefit of two decades of additional surveys since the publication of the WHO map, and more than six times the number of surveys (in spite of the stricter inclusion criteria) than were used by Nkhoma et al. (280 surveys versus 1,734). Globally, the WHO study estimates 2.6% of male newborns to be hemizygous for G6PDd alleles. As our study focused on the subset of countries with highest G6PDd prevalences (MEC versus non-MEC [31]), our MEC regional estimate (8.0%; IQR: 7.4–8.8) cannot be directly compared to the global WHO figure. However, the considerably higher regional estimate predicted here is more consistent with the recent estimate of 7.3% (95%; confidence interval: 7.0–7.6) of the global population by Nkhoma et al. [33]. Disparity between estimates may result from the population weighting used in this present study, which ensures that prevalence in densely populated regions contributes proportionally more in the regional estimate than through simple national estimate averages. Finally, this study is the first to model G6PDd prevalence in females. The previous studies discussed here, selected that 10% of heterozygous females would be diagnosed as phenotypically deficient. The flexible Bayesian model developed for the current study, and the extensive database of female survey data, enabled an empirical assessment of this spatially variable threshold. The resulting estimates, however, are subject to the same limitations as the original diagnostic tests used (Protocol S5). Diagnosing heterozygotes, who express two populations of red blood cells—normal and deficient—is highly sensitive to the enzyme activity level thresholds imposed, as the deficiency can be masked by cells expressing normal activity. The population of G6PDd cells, however, is as vulnerable to haemolytic stress as the deficient cells of hemizygotes or female homozygotes. This source of diagnostic uncertainty should be considered when interpreting these predictions of deficient females, which are based directly upon the diagnostic results.

### Model Uncertainty

The evidence-based nature of the analysis leaves the model predictions vulnerable to weaknesses in the underlying database. While some of these limitations can be quantified, such as prediction uncertainty in areas with very scarce data, others cannot. The current study presents a methodological advance over previously published maps for being the first to quantify any aspect of prediction uncertainty. In brief, our mapping procedure involved 500 repeated predictions being made from the optimised Markov chain Monte Carlo (MCMC) algorithm (Protocol S2). The median of all predicted values for each pixel is displayed in Figure 2B, and the IQR (50% confidence interval) of the repeated predictions was used to quantify model uncertainty (Figure 2C). Where model uncertainty is lowest, the 500 repeated predictions will fall within a small range, and the IQR will be correspondingly small; less straightforward predictions are associated with larger IQR values. In general, model uncertainty increases where fewer data are available and sample sizes are smaller, and where observed prevalence values are heterogeneous. This same principle applies to the population affected estimates.

Not all sources of uncertainty, however, could be accounted for by the model, which is dependent on the input dataset to represent the underlying G6PDd prevalence patterns. No global resource of genetic relatedness among populations was available, thus differences in prevalence between geographically close but genetically distant communities could only be represented in the map through the inclusion of surveys, thus, a scarcity of data may mask significant heterogeneity. For example, high prevalence of G6PDd among populations such as the endogamous groups of Orissa could not have been predicted by the model without data points from those communities. While the final dataset provides relatively good coverage, there are some large expanses lacking data where additional surveys are most needed to improve confidence in our knowledge of G6PDd prevalence, as indicated in the uncertainty map. These include several South American countries, large parts of central and southern Africa, and some highly populous Indonesian islands; the careful geopositioning of all surveys in this study allows specific gaps in the datasets to be identified that are masked in nationally aggregated maps. However, uncertainty in some of the data point geopositioning was also unaccounted for. While 80% of surveys could be mapped as points (<10 km^2^), 20% were less specific and mapped as polygons up to 35 km in radius of which centroid coordinates were used in the model (Protocol S1). The relative uncertainty introduced from this level of geopositioning uncertainty was deemed acceptable relative to the level of uncertainty, which would have been introduced by excluding those 20% of data points altogether. Finally, uncertainty in the prevalence estimates themselves stemming from the diagnostics is discussed in Protocols S1 and S5. In brief, the binary expression of normal activity versus deficiency is generally considered to be relatively reliably detected in males by most diagnostics [26],[31], though the quality of reagents and the practical difficulties of field-based settings for instance will produce some errors. As discussed previously, diagnostics for heterozygous females are altogether more complex and uncertain. The most ambiguous diagnostics for assessing the deficiency phenotype—molecular-based methods, due to the gene's extensive genetic variability—were excluded (Protocol S1).

### G6PDd Applications to Malaria Treatment

G6PDd is of pertinence to malaria treatment due to the potentially dangerous consequences of exposing G6PD deficient individuals to the vitally important anti-malarial drug primaquine. An endemicity map of *P. vivax* has recently been developed [57] indicating where this anti-relapse drug is likely to be most needed, with greatest demand being in countries targeting elimination [14]. The G6PDd map presented here can contribute to the evidence-base for weighing risk and benefit in formulating primaquine treatment strategies that could greatly accelerate the elimination of malaria transmission. We predict here that within countries targeting malaria elimination, G6PDd had an allele frequency of 5.3%, corresponding to an estimated 61 million G6PDd males and 35 million G6PDd females, with most of those occurring in Asia. However, there is evidence of a protective role for G6PDd against severe *P. falciparum* malaria [58],[59], and an effect has recently been reported against *P. vivax* parasitaemia as well [60],[61]. This being so, the prevalence of G6PDd in clinical cases of malaria may be lower than among the general population, though the precise nature of the protective effect (including which genotypes benefit) remains controversial [62],[63]. In any event, G6PDd prevalence in the broader population, as we present, remains a useful measure of the risks incurred with prescribed primaquine therapy. This may be particularly true where mass drug administration that includes primaquine is considered.

### G6PDd Severity

The diagnostic tests commonly used in community surveys determine a binary deficient/non-deficient classification; the prevalence map presented here corresponds to this binary classification, an indicator of whether primaquine may or may not be tolerated. Such diagnostics, however, cannot predict clinical severity of primaquine-induced harm, which is known to range from clinically inconsequential to life threatening [24]. More than 186 mutations have been described to the gene [64], which encode proteins expressing a spectrum of residual enzyme activity. In an attempt to encapsulate a measure of that variability in deficiency severity, we devised a simple index accounting for the relative prevalence and severity of G6PDd variants, which is intended as a guide to stratify broad categories of G6PDd-associated risk between countries and regions. However, interpretation of this analysis is constrained by major knowledge gaps. First, in relation to the evidence-base: there were no data from almost half of MECs (45 of 99) meaning that severity scores had to be inferred for many of them. Further, it is likely that reporter bias and preconceptions regarding which mutations are common, and thus worthwhile testing for, will have a strong effect on the collated database. Second, relating this index to primaquine-induced haemolytic risk assumes an inverse correlation between variant enzyme activity levels and primaquine sensitivity. Although this relationship has been found with the three variants in which the primaquine sensitivity phenotype has been characterised (A−, Mediterranean, and Mahidol [24]), further research into the association between the numerous other genetic variants and their susceptibility to primaquine is essential to substantiate this assumption. Third, the classification used here to distinguish “more severe” from “less severe” variants, in other words, the enzyme classifications into classes II and III, uses an arbitrary cut-off of 10% enzyme activity, which is not founded on clinical evidence of significance to haemolytic severity [31],[56]. It has been suggested that the distinction between these classes is blurred and may no longer be useful [65]. Fourth, the mechanism of haemolytic trigger by primaquine remains to be determined: this basic biochemical research would offer a rational basis for all of the above, and enable much more robust predictions of haemolytic risk using the datasets already collated here (of G6PDd prevalence and of the distribution of G6PDd variants).

In the absence of evidence supporting robust predictions of relative risk of severe haemolysis, residual enzyme activity is an easily obtained, albeit as yet not validated, surrogate. While such a surrogate could help inform the risk and benefit for using primaquine in any given population, in clinical practice with patients it is the dichotomy of normal versus deficient that guides primaquine treatment decisions. No treatment recommendations refer to residual enzyme activity [66]. As such, the current map of phenotypic deficiency prevalence remains the most detailed, robust, and appropriate risk assessment of overall G6PDd-associated harm, whether mild or severe, relevant to public health policies of mass primaquine administration. The insight offered by the severity index presented here corroborates the high G6PDd-associated risk that the majority of the global population at risk of *P. vivax* [57] faces.

### G6PDd in African Malaria Endemic Countries

At the continental level, highest prevalence of G6PDd is predicted across sub-Saharan Africa, where prevalence drops below 5% only on the edges of its distribution in eastern and southern Africa. In spite of being so common, the implications of G6PDd-associated primaquine reactions are not currently of major concern due to the present status of malaria control across much of the continent. High *P. falciparum* endemicity [41] means that drug policy almost exclusively targets the clinical stages. Transmission blocking therapies in such settings have not proven effective or sustainable [67]. Furthermore, the continent has relatively few people at risk of *P. vivax* [57],[68] due to the predominance of the Duffy negativity blood group [40], which is generally refractory to *P. vivax*. Thus, despite endemicity of the other relapsing human malaria, *P. ovale*, primaquine for anti-relapse is not applied in Africa [69]. However, this basis for not applying primaquine may well disappear as malaria control programmes reduce endemicity to sustainably low transmission levels, thus increasing the feasibility of elimination. When low transmission intensity is reached, policy in Africa will need to consider the treatment and practice questions now being faced in Asian and American MECs. Any primaquine treatment policy will have to account for the high prevalence of G6PDd across this continent. The G6PDd variant causing deficiency across the African population is commonly attributed to the “mild” A− mutant (Table S3) [70], and thus primaquine-associated risk of harm is thought to be minor and self-limiting [71], reflected by the moderate risk levels predicted across most of the continent (Figure 4B). However, recent evidence of low primaquine dosage triggering severe anaemia in an A– type individual (a genotype commonly considered very mildly deficient) [72], and findings from extensive DNA sequencing identifying a greater diversity of G6PD mutations than previously acknowledged [70],[73], calls for caution when using primaquine in these areas of high G6PDd prevalence, in spite of the relatively mild nature of primaquine sensitivity of the A− variants, as determined in otherwise healthy adults (rather than in children with malaria).

### G6PDd in Countries Targeting Malaria Elimination

Malaria eliminating countries (Figure 2) face steep challenges in achieving their ambitions. Prominent among these many challenges include: (i) endemic *P. vivax* malaria, and emerging resistance to chloroquine, previously the drug of choice for treating acute attacks, and recently arteminisin resistance also; (ii) high prevalence of carriers of the clinically silent and diagnostically invisible *P. vivax* hypnozoite; and (iii) the predominance of asymptomatic carriers of sexual and asexual blood stages despite low transmission intensity. The problem of *P. vivax* resistance to chloroquine is discussed elsewhere [74], but is most prevalent and threatening in south and southeast Asia [75], where its emergence greatly compounds the difficulty of the therapeutic problem [76]. A recent study along the Thai-Myanmar border [23] documented very high prevalence of *P. vivax* parasitaemia in the 63 d following therapy for acute *P. falciparum* malaria (20%–51%; correlated with drug half-life). Those rates seem to support rational and pragmatic use of anti-hypnozoiticidal primaquine treatment for all malaria patients where these parasites occur together [77]. Further, another study in the hypo-endemic Solomon Islands found that fewer than 30% of PCR-diagnosed blood infections were detected by expert microscopists, and only about 5% of infected individuals were symptomatic (overall prevalence was 9% according to PCR diagnostics but only 2.7% with microscopy) [78]. Both of these studies demonstrate the important parasite reservoir represented by asymptomatic, sub-microscopic, and latent infections, and the WHO now reconsiders its long-standing recommendation against mass drug administration as an element of malaria control [79].

Primaquine is the only chemotherapeutic tool currently available for attacking hypnozoites and mature gametocytes. As explained elsewhere [24], the available data on the safety of any regimen of primaquine may be considered almost completely inadequate by any contemporary clinical and pharmacological standards. Any MEC considering a strategy for attacking the silent hypnozoite and gametocyte reservoirs would greatly benefit from an adequate evidence-base for rational weighing of clinical risk and benefit in their areas of operations. Such an assessment may require evaluation of local G6PDd variants for vulnerability to primaquine and, ideally, point-of-care G6PDd screening to exclude those at risk of harm. Such strategies would come with substantial financial and logistical outlays, but could be most usefully directed to areas with highest potential benefit with minimal risk of harm, as indicated by the many national maps of G6PDd prevalence embedded within the global map presented here. Additional information about the severity of local variants would help support this decision-making process. The map in this study, and any subsequent iterations (worthwhile if substantial numbers of new surveys become available), provides one of the many pieces of evidence to consider when strategizing for chemotherapeutic policy aimed at elimination of transmission and relapse.

### Future Prospects and Conclusions

There is no immediate prospect of relief from the serious constraints to chemotherapeutics for malaria elimination. A new drug in phase IIb/III trials in 2012, Tafenoquine, is strategized as a successor to primaquine, but it is also likely to come with haemolytic toxicity in G6PDd patients, and thus the same constraints would apply [8]. The very brief dosing with Tafenoquine, combined with its relatively long plasma half-life, will require even greater caution in individuals affected by severe variants; though risks will be similar for patients with mild variants that lead to self-limiting haemolysis. Minimising treatment duration of primaquine from the standard 14 d has also been discussed as a means to promote course adherence and reduce risk of resistance emergence [30]. In other words, the stakes in 8-aminoquinoline therapies will increase as the commitment to elimination rises alongside a determination to attack the parasite stages that threaten success. Evaluation of risk informed by the G6PDd maps and population estimates presented here may guide appropriate investments in measures that will minimise the harm incurred by hypnozoites and gametocytes chemotherapeutics. For instance, an important potential tool in minimizing harm is a point-of-care diagnostic capable of excluding those at risk of harm caused by 8-aminoquinoline therapies. One such rapid diagnostic test in laboratory development showed promise in its first field evaluation [80]. As well as directly improving individual-level safety, such a kit may also vastly expand the available data to refine prevalence maps like that presented here, improving its resolution and margins of error. Areas where additional data would be most informative are those with highest uncertainty in the current map (Figure 3) where no, or only very few, surveys were found. Furthermore, a single diagnostic test could contribute towards standardising diagnoses and removing the potential variation between diagnostic kits, which is inherent within the current database. Although diagnosis in males is generally considered consistent with existing kits (Protocol S1), a single test would ensure this.

The prominence of G6PDd represents a barrier to current options for malaria elimination therapy. Nevertheless, the unique properties of primaquine are increasingly in demand as communities target depletion of their parasite reservoirs. It is evident that no measures are currently in place to ensure safe delivery of primaquine within the context of G6PDd risk. The complexity and diversity of both malaria epidemiology and G6PDd mean that no single solution will be applicable for ensuring safe and effective primaquine treatment. The maps and population estimates presented here represent one component of this treatment decision-making framework, and pave the way for further data collection and refinement of mapping studies of G6PDd severity. The relative urgency of this important component to determining appropriate elimination therapy may be determined by the relative prevalence of G6PDd and malaria endemicity in any given area [57],[81].

All maps at national and regional scales and in GIS and image formats, population estimates, as well as the input surveys database are freely available on the Malaria Atlas Project website (MAP; http://www.map.ox.ac.uk/).

## Supporting Information

Dataset S1 **Bibliography of sources from which surveys included in the model were identified.**
(RTF)

Protocol S1 **Assembling a global database of G6PD deficiency (G6PDd) prevalence surveys.** (S1.1) Overview of database requirements. (S1.2) Library assembly. (S1.3) Dataset inclusion criteria. (S1.4) Survey diagnostic methods. (S1.5) The final G6PDd survey dataset. (S1.6) Defining MECs' limits.
(DOCX)

Protocol S2 **Model based geostatistical framework for predicting G6PDd prevalence maps.** (S2.1) Model requirements in relation to G6PD genetics. (S2.2) The model. (S2.3) Model implementation. (S2.4) Overview of mapping procedure. (S2.5) Uncertainty.
(DOCX)

Protocol S3 **Model validation procedures and results.** (S3.1) Creation of the validation datasets. (S3.2) Model validation methodology. (S3.3) Validation results.
(DOCX)

Protocol S4 **Demographic database and population estimate procedures.** (S4.1) GRUMP-beta human population surface. (S4.2) Areal prediction procedures.
(DOCX)

Protocol S5 **Mapping the prevalence of G6PDd in females.** (S5.1) Overview of G6PDd in females. (S5.2) Heterozygous G6PDd expression and diagnosis. (S5.3) Overview of female data in the G6PD database. (S5.4) Modelling phenotypic G6PDd prevalence in females. (S5.5) Maps of G6PDd in females and population estimates. (S5.6) Improving the map of G6PDd in females.
(DOCX)

Protocol S6 **Developing an index of overall national-level risk from G6PD deficiency.** (S6.1) G6PDd variants database. (S6.2) Generating an index of national-level risk from G6PDd. (S6.3) Generating an uncertainty index of the national-level risk index categories.
(DOCX)

Table S1 **National-level demographic metrics and G6PDd allele frequency and population estimates.**
(PDF)

Table S2 **National areal prediction summary statistics and Monte Carlo standard error (SE) for each model output.**
(PDF)

Table S3 **Reported observations of class II and III G6PD variants from malaria endemic countries.**
(PDF)

## Abbreviations

G6PDd: glucose-6-phosphate dehydrogenase deficiency
GRUMP: Global Rural-Urban Mapping Project
IQR: interquartile range
MEC: malaria endemic country
PPD: posterior predictive distribution
UN: United Nations
WHO: World Health Organization
