# Supplementary material for: G6PD Deficiency Prevalence and Estimates of Affected Populations in Malaria Endemic Countries: A Geostatistical Model-Based Map
Source: PLoS Med. 2012 Nov 13;9(11):e1001339. doi: 10.1371/journal.pmed.1001339 (PMC3496665; doi:10.1371/journal.pmed.1001339)
Supplement: Dataset S1 — Bibliography of sources from which surveys included in the model were identified. (RTF) [file pmed.1001339.s001.rtf]

Dataset S1. Published and unpublished sources from which surveys were identified. Only sources reporting surveys included in the final model are listed. Population samples from these met the inclusion criteria for community representativeness, enzymatic diagnosis, sex-specificity and were geographically specific. References are listed alphabetically by first author name; n=261.


1. Abeyaratne KP, Premawansa S, Rajapakse L, Roberts DF, Pipiha SS (1976) A survey of glucose-6-phosphate-dehydrogenase deficiency in the North Central Province of Sri Lanka (formerly Ceylon). Am J Phys Anthropol 44: 135-138.
2. Abreu de Miani MS, Penalver JA (1983) [Incidence of beta-thalassemia carriers and those deficient in erythrocyte glucose-6-phosphate dehydrogenase in the greater Buenos Aires area]. Sangre (Barc) 28: 537-541.
3. Ademowo OG, Falusi AG (2002) Molecular epidemiology and activity of erythrocyte G6PD variants in a homogeneous Nigerian population. East Afr Med J 79: 42-44.
4. Ainoon O, Yu YH, Amir Muhriz AL, Boo NY, Cheong SK, et al. (2002) Glucose-6-phosphate dehydrogenase (G6PD) variants in Malaysian Malays. Hum Mutat 21: 101.
5. Akinkugbe FM (1980) Anaemia in a rural population in Nigeria (Ilora). Ann Trop Med Parasitol 74: 625-633.
6. Al Arrayed S (2005) Campaign to control genetic blood diseases in Bahrain. Community Genet 8: 52-55.
7. Ali N, Anwar M, Ayyub M, Bhatti FA, Nadeem M, et al. (2005) Frequency of glucose-6-phosphate dehydrogenase deficiency in some ethnic groups of Pakistan. J Coll Physicians Surg Pak 15: 137-141.
8. Allison AC (1960) Glucose-6-phosphate dehydrogenase deficiency in red blood cells of East Africans. Nature 186: 531-532.
9. Allison AC, Charles LJ, McGregor IA (1961) Erythrocyte glucose-6-phosphate dehydrogenase deficiency in West Africa. Nature 190: 1198-1199.
10. al-Nuaim L, Talib ZA, el-Hazmi MA, Warsy AS (1997) Sickle cell and G-6-PD deficiency gene in cord blood samples: experience at King Khalid University Hospital, Riyadh. J Trop Pediatr 43: 71-74.
11. al-Riyami A, Ebrahim GJ (2003) Genetic blood disorders survey in the Sultanate of Oman. J Trop Pediatr 49 Suppl 1: i1-20.
12. Amini F, Ismail E, Zilfalil BA (2011) Prevalence and molecular study of G6PD deficiency in Malaysian Orang Asli. Intern Med J 41: 351-353.
13. Amin-Zaki L, el-Din ST, Kubba K (1972) Glucose-6-phosphate dehydrogenase deficiency among ethnic groups in Iraq. Bull World Health Organ 47: 1-5.
14. Amoozegar H, Mirshekari M, Pishva N (2006) Does the history before blood transfusion identify donors who are glucose-6-phosphate dehydrogenase (G-6-PD) deficient? Turk J Hematol 23: 147-150.
15. Arambula E, Aguilar LJ, Vaca G (2000) Glucose-6-phosphate dehydrogenase mutations and haplotypes in Mexican Mestizos. Blood Cells Mol Dis 26: 387-394.
16. Ardati KO, Bajakian KM, Mohammad AM, Coe EL (1995) Glucose-6-phosphate-dehydrogenase phenotypes in Bahrain - quantitative-analysis and electrophoretic characterization. Saudi Med J 16: 102-104.
17. Askerova TA, Kichibekov BR, Movsum-zade KM (1992) [Hereditary glucose-6-phosphate dehydrogenase deficiency in newborn infants]. Pediatriia: 10-13.
18. Azevedo ES, Alves AF, Da Silva MC, Souza MG, Muniz Dias Lima AM, et al. (1980) Distribution of abnormal hemoglobins and glucose-6-phosphate dehydrogenase variants in 1200 school children of Bahia, Brazil. Am J Phys Anthropol 53: 509-512.
19. Azevedo ES, Costa Silva KM, Da Silva MCBO, Dias Lima AM, Mascaronhas Fortuna CM, et al. (1981) Genetic and anthropological studies in the island of Itaparica, Bahia, Brazil. Hum Hered 31: 353-357.
20. Azhar A (1998) Kajian genetika biokemis dehidrogenase glukosa-6-fosfat (G6PD) dan dehidrogenase 6-fosfoglukonat (6-PGD) pada tiga populasi Nusa Tenggara. Yogyakarta: Universitas Gadjah Mada.
21. Azhar A, Husin A (2001) Prevalence of glucose 6-phosphate dehydrogenase (G6PD) deficiency in two populations of Aceh province. Jurnal Kedokteran Yarsi 9: 93-95.
22. Azim AA, Kamel K, Gaballah MF, Sabry FH, Ibrahim W, et al. (1974) Genetic blood markers and anthropometry of the populations in Aswan Governorate, Egypt. Hum Hered 24: 12-23.
23. Azofeifa J, Barrantes R (1991) Genetic variation in the Bribri and Cabecar Amerindians from Talamanca, Costa Rica. Rev Biol Trop 39: 249-253.
24. Badens C, Leclaire M, Collomb J, Auquier P, Soyer P, et al. (2001) [Glucose-6-phosphate dehydrogenase et neonatal jaundice]. Presse Med 30: 524-526.
25. Baer A, Lie-Injo LE, Welch QB, Lewis AN (1976) Genetic factors and malaria in the Temuan. Am J Hum Genet 28: 179-188.
26. Balgir RS (2007) Genetic burden of red cell enzyme glucose-6-phosphate dehydrogenase deficiency in two major Scheduled Tribes of Sundargarh district, Northwestern Orissa, India. Current Science 92: 768-774.
27. Balgir RS, Sharma JC (1988) Genetic markers in the Hindu and Muslim Gujjars of Northwestern India. Am J Phys Anthropol 75: 391-403.
28. Banerjee B, Saha N, Daoud ZF, Khalaf FH, Qudah H (1981) A genetic study of the Jordanians. Hum Hered 31: 65-69.
29. Barretto OC, Nonoyama K (1978) [Malaria-dependent polymorphism related to erythrocyte glucose-6-phosphate dehydrogenase and glutathione among Brazilian Indians]. Rev Hosp Clin Fac Med Sao Paulo 33: 231-233.
30. Barretto OCO (1970) Erythrocyte glucose-6-phosphate dehydrogenase deficiency in Sao Paulo, Brazil. Rev Bras Pesqui Med Biol 3: 61-65.
31. Basu S, Jindal A, Kumar CS, Khan AS (1995) Genetic marker profile of primitive Kutia Kondh tribal population of Phulbani district (Orissa). Indian J Med Res 101: 36-38.
32. Bayoumi RA, Taha TS, Saha N (1985) A study of some genetic characteristics of the Fur and Baggara tribes of the Sudan. Am J Phys Anthropol 67: 363-370.
33. Beck HP, Felger I, Kabintik S, Tavul L, Genton B, et al. (1994) Assessment of the humoral and cell-mediated immunity against the Plasmodium falciparum vaccine candidates circumsporozoite protein and SPf66 in adults living in highly endemic malarious areas of Papua New Guinea. Am J Trop Med Hyg 51: 356-364.
34. Benabadji M, Benlatrache C, Merad F, Suaudeau C, Benmoussa M, et al. (1977) [Glucose-6-phosphate dehydrogenase deficiency in Algeria.]. Sem Hop 53: 899-904.
35. Bernstein RE (1965) Inborn errors of metabolism in Central Africa: red cell glucose-6-phosphate dehydrogenase deficiency and sickle haemoglobin. In: GJ S, editor. Science and Medicine in Central Africa. NY: Pergamon Press. pp. 739-747.
36. Bernstein SC, Bowman JE, Kaptue Noche L (1980) Population studies in Cameroon: hemoglobin S, glucose-6-phosphate dehydrogenase deficiency and falciparum malaria. Hum Hered 30: 251-258.
37. Bernstein SC, Bowman JE, Noche LK (1980) Interaction of sickle cell trait and glucose-6-phosphate dehydrogenase deficiency in Cameroon. Hum Hered 30: 7-11.
38. Best WR (1959) Absence of erythrocyte glucose-6-phosphate dehydrogenase deficiency in certain Peruvian Indians. J Lab Clin Med 54: 791.
39. Bienzle U, Ayeni O, Lucas AO, Luzzatto L (1972) Glucose-6-phosphate dehydrogenase and malaria. Greater resistance of females heterozygous for enzyme deficiency and of males with non-deficient variant. Lancet 1: 107-110.
40. Bienzle U, Okoye VC, Gogler H (1972) Haemoglobin and glucose-6-phosphate dehydrogenase variants: distribution in relation to malaria endemicity in a Togolese population. Z Tropenmed Parasitol 23: 56-62.
41. Blibech R, Gharbi Y, Mrad A, Zahra H, Mahjoub T, et al. (1989) Incidence of glucose-6-phosphate dehydrogenase (G6PD) deficiency in Tunisian populations. Nouv Rev Fr Hematol 31: 189-191.
42. Blinov MN, Rodriges N, Sanches Perovani Kh A (1973) [Incidence in the glucose-6-phosphate dehydrogenase deficiency of erythrocytes in the population of North Oriente province (Republic of Cuba)]. Probl Gematol Pereliv Krovi 18: 26-29.
43. Bloch M, Rivera H (1969) [Abnormal hemoglobins and glucose-6-phosphate dehydrogenase deficiency in El Salvador]. Sangre (Barc) 14: 121-124.
44. Bonne B, Godber M, Ashbel S, Mourant AE, Tills D (1971) South-Sinai Beduin. A preliminary report on their inherited blood factors. Am J Phys Anthropol 34: 397-408.
45. Bottini N, Meloni G, Porcu S, Gloria-Bottini F (2001) Cyclic seasonal variation of G-6-PD deficiency in newborn infants from Sardinia. Biol Rhythm Res 32: 413-421.
46. Bowman JE, Paul E. Carson, Henri Frischer, Robin D. Powell, Edward J. Colwell, et al. (1971) Hemoglobin and red cell enzyme variation in some populations of the republic of Vietnam with comments on the malaria hypothesis. Am J Phys Anthropol 34: 313-324.
47. Brabin L, Brabin BJ (1990) Malaria and glucose 6-phosphate dehydrogenase deficiency in populations with high and low spleen rates in Madang, Papua New Guinea. Hum Hered 40: 15-21.
48. Buchanan JG, Wilson FS, Nixon AD (1973) Survey for erythrocyte glucose-6-phosphate dehydrogenase deficiency in Fiji. Am J Hum Genet 25: 36-41.
49. Budtz-Olsen O, Kidson C (1961) Absence of red cell enzyme deficiency in Australian Aborigines. Nature 192: 765.
50. Cao A, Congiu R, Sollaino MC, Desogus MF, Demartis FR, et al. (2008) Thalassaemia and glucose-6-phosphate dehydrogenase screening in 13- to 14-year-old students of the Sardinian population: preliminary findings. Community Genet 11: 121-128.
51. Castaneda BF, Colwell EJ, Phintuyothin P, Hickman RJ (1972) Investigations of the fluorescent spot test for erythrocyte glucose-6-phosphate dehydrogenase deficiency in southeast Thailand. J Med Assoc Thai 55: 331-338.
52. Ceda-Flores RM, Arriaga-Rios G, Munoz-Campos J, Bautista-Pena VA, Angeles Rojas-Alvarado M, et al. (1990) [Frequency of color blindness and glucose-6-phosphate dehydrogenase enzyme deficiency in non-industrialized populations in the state of Nuevo Leon, Mexico]. Arch Invest Med (Mex) 21: 229-234.
53. Chan TK (1983) Glucose 6 phosphate dehydrogenase (G6PD) [MD Thesis]: University of Hong Kong.
54. Chan YK, Tay MT, Lim MK (1992) Xq28: epidemiology and sex-linkage between red-green colour blindness and G6PD deficiency. Ann Acad Med Singapore 21: 318-322.
55. Chien YH, Lee NC, Wu ST, Liou JJ, Chen HC, et al. (2008) Changes in incidence and sex ratio of glucose-6-phosphate dehydrogenase deficiency by population drift in Taiwan. Southeast Asian J Trop Med Public Health 39: 154-161 & additional data from authors.
56. Choremis C, Fessas P, Kattamis C, Stamatoyannopoulos G, Zannos-Mariolea L, et al. (1963) Three inherited red-cell abnormalities in a district of Greece. Thalassaemia, sickling, and glucose-6-phosphate-dehydrogenase deficiency. Lancet 1: 907-909.
57. Choremis C, Zannos-Mariolea L, Katamis MD (1962) Frequency of glucose-6-phosphate-dehydrogenase deficiency in certain highly malarious areas of Greece. Lancet 1: 17-18.
58. Chowdhury A (1976) Glucose-6-phosphate-dehydrogenase in a Bengalee sample of Calcutta. Man India 56: 263-268.
59. Cladera Serra A, Oliva Berini E, Torrent Quetglas M, Bartolozzi Castilla E (1997) [Prevalence of glucose-6-phosphate dehydrogenase deficiency in a student population on the island of Menorca]. Sangre (Barc) 42: 363-367.
60. Cocco P, Manca P, Dessi S (1987) Preliminary results of a geographic correlation study on G6PD deficiency and cancer. Toxicol Pathol 15: 106-108.
61. Compri MB, Saad ST, Ramalho AS (2000) [Genetico-epidemiological and molecular investigation of G-6-PD deficiency in a Brazilian community]. Cad Saude Publica 16: 335-342.
62. Daar S, Vulliamy TJ, Kaeda J, Mason PJ, Luzzatto L (1996) Molecular characterization of G6PD deficiency in Oman. Hum Hered 46: 172-176.
63. DaCosta H, Pattani J, Dandekar S, Kotnis U, Mehendale K, et al. (1967) Glucose-6-phosphate dehydrogenase (G-6-PD) defect in Maharashtrian children. Indian J Med Sci 21: 809-812.
64. David S, Trincao C (1963) [Drepanocytemia, erythrocytic glucose-6-phosphate dehydrogenase deficiency (G-6-Pd) and malaria in the Cuango Post (Lunda-Angola).]. An Inst Med Trop (Lisb) 20: 5-15.
65. Devi ST, Saran SK, Nair G (1993) Study of glucose-6-phosphate dehydrogenase (G6PD) in the Kissan tribals of Orissa and the Kannikar tribals of Kerala, India. Anthropol Anz 51: 179-181.
66. Diatewa M, Ganga-Zanzou SP, Gangoue N, Miehakanda J (1992) [Neonatal icterus and erythrocyte glucose-6-phosphate dehydrogenase deficiency in Congolese newborn infants in Brazzaville]. Arch Fr Pediatr 49: 939-940.
67. Doeblin TD, Ingall GB, Pinkerton PH, Donambaju KR, Bannerman RM (1968) Genetic studies of the Seneca Indians: haptoglobins, transferrins, G-6-PD Deficiency, hemoglobinopathy, color blindness, morphological traits and dermatoglyphics. Hum Hered 18: 251-260.
68. Doxiadis SA, Karaklis A, Valaes T, Stavrakakis D (1964) Risk of severe jaundice in glucose-6-phosphate-dehydrogenase deficiency of the newborn. Differences in population groups. Lancet 2: 1210-1212.
69. Dube RK, Dube B, Gupta YN (1976) Erythrocytic glucose-6-phosphate dehydrogenase deficiency at Varanasi. Indian J Pathol Microbiol 19: 245-251.
70. Duflo B, Diallo A, Toure K, Soula G (1979) [Glucose-6-phosphate dehydrogenase deficiency in Mali. Epidemiology and pathological aspects]. Bull Soc Pathol Exot Filiales 72: 258-264.
71. Duflo B, Ranque P, Quilici M, Balique H, Dembele O, et al. (1982) Glucose-6-phosphate-dehydrogenase deficiency and malaria in Mali. Nouvelle Presse Medicale 11: 2713-2713.
72. Egesie OJ, Joseph DE, Isiguzoro I, Egesie UG (2008) Glucose-6-phosphate dehydrogenase (G6PD) activity and deficiency in a population of Nigerian males resident in Jos. Niger J Physiol Sci 23: 9-11.
73. El-Hazmi MA, Warsy AS (1987) Interaction between glucose-6-phosphate dehydrogenase deficiency and sickle cell gene in Saudi Arabia. Trop Geogr Med 39: 32-35.
74. El-Hazmi MAF, Warsy AS (1997) Phenotypes of glucose-6-phosphate dehydrogenase in different regions of Saudi Arabia - A comparative assessment. Saudi Med J 18: 393-399.
75. El-Migdadi F, Al-Tellawi A, Al-Hussain S, Rawashdeh M (2008) Pyruvate kinase and glucose-6-phosphate dehydrogenase activities in children living above (Jordan Valley) and below (Amman and Irbid) sea level. Journal of Chinese Clinical Medicine 3: 627-632.
76. Eng LL, Giok PH (1964) Glucose-6-phosphate dehydrogenase deficiency in Indonesia. Nature 204: 88-89.
77. Eng LL, Ti TS (1964) Glucose-6-phosphate dehydrogenase deficiency in Malayans. Trans R Soc Trop Med Hyg 58: 500-502.
78. Estrada M, Gonzalez R (1983) [Neonatal jaundice and glucose-6-phosphate dehydrogenase deficiency in Havana]. Rev Invest Clin 35: 297-299.
79. Fernando WP, Ratnapala PR (1988) Report: A survey to ascertain the prevalence of G-6-PD enzyme deficiency in Sri Lanka, and its geographical and ethnical distribution. funded by ISTI.
80. Flatz G, Chakravartti MR, Das BM, Delbruck H (1972) Genetic survey in the population of Assam. I. ABO blood groups, glucose-6-phosphate dehydrogenase and haemoglobin type. Hum Hered 22: 323-330.
81. Flatz G, Sringam S (1963) Malaria and glucose-6-phosphate dehydrogenase deficiency in Thailand. Lancet 2: 1248-1250.
82. Flatz G, Sringam S, Premyothin C, Penbharkkul S, Ketusingh R, et al. (1963) Glucose-6-phosphate dehydrogenase deficiency and neonatal jaundice. Arch Dis Childh 38: 566-570.
83. Fraser GR, Defaranas B, Kattamis CA, Race RR, Sanger R, et al. (1964) Glucose-6-phosphate dehydrogenase, colour vision and Xg blood groups in Greece: linkage and population data. Ann Hum Genet 27: 395-403.
84. Fraser GR, Grunwald P, Stamatoyannopoulos G (1966) Glucose-6-phosphate dehydrogenase (G6PD) deficiency, abnormal haemoglobins, and thalassaemia in Yugoslavia. J Med Genet 3: 35-41.
85. Fraser GR, Stamatoyannopoulos G, Kattamis C, Loukopoulos D, Defaranas B, et al. (1964) Thalassemias, abnormal hemoglobins and glucose-6-phosphate dehydrogenase deficiency in the Arta area of Greece: diagnostic and genetic aspects of complete village studies. Ann N Y Acad Sci 119: 415-435.
86. Fraser GR, Steinberg AG, Defaranas B, Mayo O, Stamatoyannopoulos G, et al. (1969) Gene frequencies at loci determining blood-group and serum-protein polymorphisms in two villages of northwestern Greece. Am J Hum Genet 21: 46-60.
87. Ganczakowski M, Town M, Bowden DK, Vulliamy TJ, Kaneko A, et al. (1995) Multiple glucose 6-phosphate dehydrogenase-deficient variants correlate with malaria endemicity in the Vanuatu archipelago (southwestern Pacific). Am J Hum Genet 56: 294-301.
88. Garcia SC, Moragon AC, Lopez-Fernandez ME (1979) Frequency of glutathione reductase, pyruvate kinase and glucose-6-phosphate dehydrogenase deficiency in a Spanish population. Hum Hered 29: 310-313.
89. Garg A, Bhatia BD, Chaturvedi P, Garg S (1984) G6PD deficiency in newborn infants. Indian J Pediatr 51: 29-33.
90. Garlipp CR, Ramalho AS (1988) [Clinical and laboratory aspects of glucose-6-phosphate-dehydrogenase (G-6-Pd) deficiency in Brazilian newborns]. Rev Bras Genet 11: 717-728.
91. Geerdink RA, Bartstra HA, Schillhorn Van Veen JM (1974) Serum proteins and red cell enzymes in Trio and Wajana Indians from Surinam. Am J Hum Genet 26: 581-587.
92. Geerdink RA, Okhura K, Li Fo Sjoe E, Schillhorn van Veen JM, Bartstra HA (1975) Serum factors and red cell enzymes in Carib and Arowak Indians from Surinam. Trop Geogr Med 27: 269-273.
93. Gelpi AP (1965) Glucose-6-phosphate dehydrogenase deficiency in Saudi Arabia: a survey. Blood 25: 486-493.
94. Gelpi AP (1967) Glucose-6-phosphate dehydrogenase deficiency, the sickling trait, and malaria in Saudi Arab children. J Pediatr 71: 138-146.
95. Ghosh K, Mukherjee MB, Shankar U, Kote SL, Nagtilak SB, et al. (2002) Clinical examination and hematological data in asymptomatic & apparently healthy school children in a boarding school in a tribal area. Indian J Public Health 46: 61-65.
96. Gibbs WN, Ottey F, Dyer H (1972) Distribution of glucose-6-phosphate dehydrogenase phenotypes in Jamaica. Am J Hum Genet 24: 18-23.
97. Giles E, Curtain CC, Baumgarten A (1967) Distribution of beta-thalassemia trait and erythrocyte glucose-6-phosphate dehydrogenase deficiency in the Markham River Valley of New Guinea. Am J Phys Anthropol 27: 83-88.
98. Gualandri V, Orsini GB, Porta E, Gerli GC (1983) [Glucose-6-phosphate dehydrogenase deficiency among the student population of Milan]. J Genet Hum 31: 201-209.
99. Gupta JC, Yagnik U, Seth P (1982) Incidence of glucose-6-phosphate dehydrogenase deficiency in Jabalpur area. Indian J Pathol Microbiol 25: 66-69.
100. Gupta S, Ghai OP, Chandra RK (1970) Glucose-6-phosphate dehydrogenase deficiency in the newborn and its relation to serum bilirubin. Indian J Pediatr 37: 169-176.
101. Gupte SC, Shaw AN, Shah KC (2005) Hematological findings and severity of G6PD deficiency in Vataliya Prajapati subjects. J Assoc Physicians India 53: 1027-1030.
102. Haghighi B, Suzangar M, Yazdani A, Mehnat M (1985) A genetic variant of human erythrocyte glucose 6-phosphate dehydrogenase. Biochem Biophys Res Commun 132: 1151-1159.
103. Harley JD, Agar NS, Turner TB (1976) Letter: sickle-cell anaemia and trait in Sydney. Med J Aust 1: 894.
104. Hashmi JA, Farzana F, Ahmed M (1976) Abnormal hemoglobins, thalasemia trait & G6PD deficiency in young Pakistani males. J Pak Med Assoc 26: 2-4.
105. Hoan NKH (2010) Personal communication: unpublished data from Vietnam.
106. Hussein L, Yamamah G, Saleh A (1992) Glucose-6-phosphate dehydrogenase deficiency and sulfadimidin acetylation phenotypes in Egyptian oases. Biochem Genet 30: 113-121.
107. Ibrahim WN, Kamel K, Selim O, Azim A, Gaballah MF, et al. (1974) Hereditary blood factors and anthropometry of the inhabitants of the Egyptian Siwa Oasis. Hum Biol 46: 57-68.
108. Idel'son LI, Kotoian ER (1970) [Incidence of glucose-6-phosphate dehydrogenase deficiency in erythrocytes of the population in Armenia]. Probl Gematol Pereliv Krovi 15: 39-44.
109. Itskan SB, Saldanha PH (1975) [Erythrocyte glucose-6-phosphate dehydrogenase activity in the population of a malarial region in Sao Paulo (Iguape)]. Rev Inst Med Trop Sao Paulo 17: 83-91.
110. Jenkins T, Blecher SR, Smith AN, Anderson CG (1968) Some hereditary red-cell traits in Kalahari Bushmen and Bantu: hemoglobins, glucose-6-phosphate dehydrogenase deficiency, and blood groups. Am J Hum Genet 20: 299-309.
111. Jeremiah ZA, Uko EK, Usanga EA (2008) Relation of nutritional status, sickle cell trait, glucose-6-phosphate dehydrogenase deficiency, iron deficiency and asymptomatic malaria infection in the Niger Delta, Nigeria. J Med Sci 8: 269-274.
112. Jiang J, Ma X, Song C, Lin B, Cao W, et al. (2003) Using the fluorescence spot test for neonatal screening of G6PD deficiency. Southeast Asian J Trop Med Public Health 34 Suppl 3: 140-142.
113. Jiang W, Yu G, Liu P, Geng Q, Chen L, et al. (2006) Structure and function of glucose-6-phosphate dehydrogenase-deficient variants in Chinese population. Hum Genet 119: 463-478.
114. Johnson MK, Clark TD, Njama-Meya D, Rosenthal PJ, Parikh S (2009) Impact of the method of G6PD deficiency assessment on genetic association studies of malaria susceptibility. PLoS One 4: e7246.
115. Jolly JG, Sarup BM, Bhatnagar DP, Maini SC (1972) Glucose-6-phosphate dehydrogenase deficiency in India. J Indian Med Assoc 58: 196-200.
116. Kageoka T, Satoh C, Goriki K, Fujita M, Neriishi S, et al. (1985) Electrophoretic variants of blood proteins in Japanese. IV. Prevalence and enzymologic characteristics of glucose-6-phosphate dehydrogenase variants in Hiroshima and Nagasaki. Hum Genet 70: 101-108.
117. Kamal I, Gabr M, Mohyeldin O, Talaat M (1967) Frequency of glucose6-phosphate dehydrogenase deficiency in Egyptian children. Acta Genet Stat Med 17: 321-327.
118. Kamel K, Umar M, Ibrahim W, Mansour A, Gaballah F, et al. (1975) Anthropological studies among Libyans. Erythrocyte genetic factors, serum haptoglobin phenotypes and anthropometry. Am J Phys Anthropol 43: 103-111.
119. Kaneko A, Taleo G, Kalkoa M, Yaviong J, Reeve PA, et al. (1998) Malaria epidemiology, glucose 6-phosphate dehydrogenase deficiency and human settlement in the Vanuatu Archipelago. Acta Trop 70: 285-302.
120. Kaplanoglou LB, Triantaphyllidis CD (1982) Genetic polymorphisms in a North-Greek population. Hum Hered 32: 124-129.
121. Kate SL, Mukherjee BN, Malhotra KC, Phadke MA, Mutalik GS, et al. (1978) Red cell glucose-6-phosphate dehydrogenase deficiency and haemoglobin variants among ten endogamous groups of Maharshtra and West Bengal. Hum Genet 44: 339-343.
122. Kate SL, Phadke MA, Sainani GS, Mutalik GS (1976) Study of erythrocyte glucose-6-phosphate dehydrogenase and abnormal haemoglobins in an endogamous community--"Katkaris"--a survey. J Assoc Physicians India 24: 1-3.
123. Kattamis CA, Chaidas A, Chaidas S (1969) G6PD deficiency and favism in the island of Rhodes (Greece). J Med Genet 6: 286-291.
124. Kidson C (1961) Deficiency of glucose-6-phosphate dehydrogenase: some aspects of the trait in people of Papua-New Guinea. Med J Aust 48(2): 506-509.
125. Kigoni EP, Kujwalile JM, Nhonoli AM (1978) Frequency of glucose-6-phosphate dehydrogenase (G-6-PD) deficiency and its relationship to hepatitis B surface antigen (HBS-Ag) in normal Tanzanian males. East Afr Med J 55: 247-251.
126. Kirk RL, Keats B, Blake NM, McDermid EM, Ala F, et al. (1977) Genes and people in the Caspian Littoral: a population genetic study in Northern Iran. Am J Phys Anthropol 46: 377-390.
127. Kirkman HN, Walker DH (1982) Glucose-6-phosphate-dehydrogenase deficiency and Mediterranean fever in northern Sardinia - Reply. J Infect Dis 146: 302-302.
128. Knight RH, Robertson DH (1963) The prevalence of the erythrocyte glucose-6-phosphate dehydrogenase deficiency among Africans in Uganda. Trans R Soc Trop Med Hyg 57: 95-100.
129. Kotea R, Kaeda JS, Yan SL, Sem Fa N, Beesoon S, et al. (1999) Three major G6PD-deficient polymorphic variants identified among the Mauritian population. Br J Haematol 104: 849-854.
130. Krasnopol'skaia KD, Filippov IK, Sotnikova EN, Movsum-zade KM, Gadzhiev BO (1980) [Patterns in the distribution of GPD- alleles in Azerbaijan. I. Incidence and polymorphism of glucose-6-phosphate dehydrogenase deficiency in the Shekii region of the Azerbaijan SSR]. Genetika 16: 1685-1692.
131. Krasnopol'skaia KD, Iakovlev SA, Smirnova OA, Prytkov AN (1985) [Patterns of Gd- allele distribution in Azerbaijan. IV. The incidence and polymorphism of erythrocytic glucose-6-phosphate dehydrogenase deficiency in the settlement of Kobi, Apsheron District]. Genetika 21: 487-492.
132. Krasnopolskaya XD, Shatskaya TL (1987) Distribution of Gd- alleles in some ethnic groups of the USSR. Hum Genet 75: 258-263.
133. Krželj V, Markiæ J, Karaman K, Æurin K, Uniæ I, et al. (2011) Personal communication: unpublished data from Croatia.
134. Kuhn VL, Lisboa V, de Cerqueira LP (1983) [Glucose-6-phosphate dehydrogenase deficiency in blood donors in a general hospital of Salvador, Bahia, Brazil]. Rev Paul Med 101: 175-177.
135. Kumakawa T, Suzuki S, Fujii H, Miwa S (1987) Frequency of glucose 6-phosphate dehydrogenase (G6PD) deficiency in Tokyo and a new variant: G6PD Musashino. Nippon Ketsueki Gakkai Zasshi 50: 25-28.
136. Kuwahata M, Wijesinghe R, Ho MF, Pelecanos A, Bobogare A, et al. (2010) Population screening for glucose-6-phosphate dehydrogenase deficiencies in Isabel Province, Solomon Islands, using a modified enzyme assay on filter paper dried bloodspots. Malar J 9: 223.
137. Lai HC, Lai MP, Leung KS (1968) Glucose-6-phosphate dehydrogenase deficiency in Chinese. J Clin Pathol 21: 44-47.
138. Le Xuan C, Le Si Q, Humbert C, Chu Quang G (1968) [Glucose-6-phosphate dehydrogenase deficiency in Viet-nam]. Nouv Rev Fr Hematol 8: 878-884.
139. Lefevre-Witier P, Vergnes H (1977) Enzyme polymorphisms of Ideles populations (Ahaggar, Algeria) and the Iwellemeden Kel Kummer Twaregs (Menaka, Mali). Hum Hered 27: 454-469.
140. Lehmann H, Ala F, Hedeyat S, Montazemi K, Nejad HK, et al. (1973) Biological studies of Yemenite and Kurdish Jews in Israel and other groups in south-west Asia. XI. The hereditary blood factors of the Kurds of Iran. Phil Trans R Soc London B 266: 195-205.
141. Lisker R, Cordova MS, Graciela Zarate QB (1969) Studies on several genetic hematological traits of the Mexican population. XVI. Hemoglobin, S and glucose-6-phosphate dehydrogenase deficiency in the east coast. Am J Phys Anthropol 30: 349-354.
142. Lisker R, Loria A, Cordova MS (1965) Studies on several genetic hematological traits of the Mexican population. VIII. Hemoglobin S, glucose-6-phosphate dehydrogenase deficiency, and other characteristics in a malarial region. Am J Hum Genet 17: 179-187.
143. Lisker R, Loria A, Gonzales Llaven J, Guttman S, Ruiz Reyes G (1962) [Preliminary note on the incidence of abnormal hemoglobulins and glucose-6-phosphate dehydrogenase deficiency in the Mexican population.]. Rev Fr Etud Clin Biol 7: 76-78.
144. Lothe F (1967) Erythrocyte glucose-6-phosphate dehydrogenase deficiency in Uganda. Nature 215: 299-300.
145. Lysenko A, Abrashkin-Zhuchkov RG, Alekseeva MI, Gorbunova Iu P, Krasil'nikov AA (1973) [Incidence of hereditary deficiency of glucose-6-phosphate dehydrogenase activity of erythrocytes in Azerbaijan SSR]. Probl Gematol Pereliv Krovi 18: 16-21.
146. Madanat F, Karadsheh N, Shamayleh A, Tarawneh M, Khraisha S, et al. (1986) Glucose-6-phosphate dehydrogenase deficiency in male newborns. Jordan Medical Journal 21: 205-212.
147. Markic J, Krzelj V, Markotic A, Marusic E, Stricevic L, et al. (2006) High incidence of glucose-6-phosphate dehydrogenase deficiency in Croatian island isolate: example from Vis island, Croatia. Croat Med J 47: 556-570.
148. Martinez-Labarga C, Rickards O, Scacchi R, Corbo RM, Biondi G, et al. (1999) Genetic population structure of two African-Ecuadorian communities of Esmeraldas. Am J Phys Anthropol 109: 159-174.
149. Martins MC, Olim G, Melo J, Magalhaes HA, Rodrigues MO (1993) Hereditary anaemias in Portugal: epidemiology, public health significance, and control. J Med Genet 30: 235-239.
150. Mathews ST, Kumaresan PR, Selvam R (1991) Glucose-6-phosphate dehydrogenase deficiency and malaria--a study on north Madras population. J Commun Dis 23: 178-181.
151. Matsuoka H, Arai M, Yoshida S, Tantular IS, Pusarawati S, et al. (2003) Five different glucose-6-phophate [correction phosphate]dehydrogenase (G6PD) variants found among 11 G6PD-deficient persons in Flores Island, Indonesia. J Hum Genet 48: 541-544.
152. Matsuoka H, Ishii A, Panjaitan W, Sudiranto R (1986) Malaria and glucose-6-phosphate dehydrogenase deficiency in North Sumatra, Indonesia. Southeast Asian J Trop Med Public Health 17: 530-536.
153. Matsuoka H, Nguon C, Kanbe T, Jalloh A, Sato H, et al. (2005) Glucose-6-phosphate dehydrogenase (G6PD) mutations in Cambodia: G6PD Viangchan (871G>A) is the most common variant in the Cambodian population. J Hum Genet 50: 468-472.
154. Mayer G, Mayoux A (1966) Recherches d'anomalies sanguines genetiques dans une population de la cote Est de Madagascar. Arch Inst Pasteur Madagascar 35: 209-211.
155. McGuinness R, Saunders RA (1967) Erythrocyte galactose-I-phosphate uridyl transferase and glucose-6-phosphate dehydrogenase activity in the population of the Rhondda Fach. Clin Chim Acta 16: 221-226.
156. Ménard D (2011) Personal communication: unpublished data from Cambodia.
157. Miall WE, Milner PF, Lovell HG, Standard KL (1967) Haematological investigations of population samples in Jamaica. Br J Prev Soc Med 21: 45-55.
158. Miguel A, Ramon M, Petitpierre E, Goos CM, Vermeesch-Markslag AM, et al. (1983) Population screening for glucose-6-phosphate dehydrogenase deficiency on the Baleares. Hum Genet 64: 176-179.
159. Milbauer B, Peled N, Svirsky S (1973) Neonatal hyperbilirubinemia and glucose-6-phosphate dehydrogenase deficiency. Isr J Med Sci 9: 1547-1552.
160. Mir NA, Fakhri M, Abdelaziz M, Kishan J, Elzouki A, et al. (1985) Erythrocyte glucose-6-phosphate dehydrogenase status of newborns and adults in eastern Libya. Ann Trop Paediatr 5: 211-213.
161. Mohammed N, Amanzai O, Rashid H, Jan S, Leslie T (2010) Report: Assessment of the prevalence of G6PD deficiency in Afghanistan. HealthNet TPO Malaria Control Programme, funded by a Global Fund for AIDS TB and Malaria Round 5 grant.
162. Monchy D, Babin FX, Srey CT, Ing PN, von Xylander S, et al. (2004) [Frequency of G6PD deficiency in a group of preschool-aged children in a centrally located area of Cambodia]. Med Trop (Mars) 64: 355-358.
163. Mortazavi YM, Soleimani MS, Lahijani ANA, Omidkhoda AO, Ghavamzadeh A (2008) The Frequency and Molecular Genetics of G6pd Deficiency in Northwest and Southeast of Iran. Haematol-Hematol J 93: 485-486.
164. Moscarelli G, Ferraro G, Infantone MA, Scola S, Montaperto A, et al. (1999) Screening della glucosio 6 fosfato deidrogenasi in 500 gravide. Giornale Italiano di Ostetricia e Ginecologia 21: 27-28.
165. Motulsky AG, Vandepitte J, Fraser GR (1966) Population genetic studies in the Congo. I. Glucose-6-phosphate dehydrogenase deficiency, hemoglobin S, and malaria. Am J Hum Genet 18: 514-537.
166. Mourant AE, Kopec AC, Ikin EW, Lehmann H, Bowen-Simpkins P, et al. (1974) The blood groups and haemoglobins of the Kunama and Baria of Eritrea, Ethiopia. Ann Hum Biol 1: 383-392.
167. Murhekar KM, Murhekar MV, Mukherjee MB, Gorakshakar AC, Surve R, et al. (2001) Red cell genetic abnormalities, beta-globin gene haplotypes, and APOB polymorphism in the Great Andamanese, a primitive Negrito tribe of Andaman and Nicobar Islands, India. Hum Biol 73: 739-744.
168. Muzaffer MA (2005) Neonatal screening of glucose-6-phosphate dehydrogenase deficiency in Yanbu, Saudi Arabia. J Med Screen 12: 170-171.
169. Nasserullah Z, Al Jame A, Abu Srair H, Al Qatari G, Al Naim S, et al. (1998) Neonatal screening for sickle cell disease, glucose-6-phosphate dehydrogenase deficiency and a-thalassemia in Qatif and Al Hasa. Ann Saudi Med 18: 289-292.
170. Neto JPD, Dourado MV, dos Reis MG, Goncalves MS (2008) A novel c. 197T -> A variant among Brazilian neonates with glucose-6-phosphate dehydrogenase deficiency. Genet Mol Biol 31: 33-35.
171. Nezhad SRK, Mashayekhi A, Khatami SR, Daneshmand S, Fahmi F, et al. (2009) Prevalence and molecular identification of mediterranean glucose-6-phosphate dehydrogenase deficiency in Khuzestan Province, Iran. Iran J Public Health 38: 127-131.
172. Nicolielo DB, Ferreira RIP, Leite AA (2006) Activity of 6-phosphogluconate dehydrogenase in glucose-6-phosphate dehydrogenase deficiency. Rev Bras Hematol Hemoter 28: 135-138.
173. Nieuwenhuis F, Wolf B, Bomba A, De Graaf P (1986) Haematological study in Cabo Delgado province, Mozambique; sickle cell trait and G6PD deficiency. Trop Geogr Med 38: 183-187.
174. Ninokata A, Kimura R, Samakkarn U, Settheetham-Ishida W, Ishida T (2006) Coexistence of five G6PD variants indicates ethnic complexity of Phuket islanders, Southern Thailand. J Hum Genet 51: 424-428.
175. Nixon AD, Buchanan JG (1969) Survey for erythrocyte glucose-6-phosphate dehydrogenase deficiency in Polynesians. Am J Hum Genet 21: 305-309.
176. Nosten F, Bancone G (2011) Personal communication: unpublished data from Thailand.
177. Nuchprayoon I, Louicharoen C, Charoenvej W (2008) Glucose-6-phosphate dehydrogenase mutations in Mon and Burmese of southern Myanmar. J Hum Genet 53: 48-54.
178. Nuchprayoon I, Sanpavat S, Nuchprayoon S (2002) Glucose-6-phosphate dehydrogenase (G6PD) mutations in Thailand: G6PD Viangchan (871G>A) is the most common deficiency variant in the Thai population. Hum Mutat 19: 185.
179. Nurse GT, Jenkins T (1977) Serogenetic studies on the Kavango peoples of South West Africa. Ann Hum Biol 4: 465-478.
180. Nurse GT, Jenkins T, David JH, Steinberg AG (1979) The Njinga of Angola: a serogenetic study. Ann Hum Biol 6: 337-348.
181. Nwankwo MU, Bunker CH, Ukoli FA, Omene JA, Freeman DT, et al. (1990) Blood pressure and other cardiovascular disease risk factors in black adults with sickle cell trait or glucose-6-phosphate dehydrogenase deficiency. Genet Epidemiol 7: 211-218.
182. Ohkura K, Miyashita T, Nakajima H, Matsumoto H, Matsutomo K, et al. (1984) Distribution of polymorphic traits in Mazandaranian and Guilanian in Iran. Hum Hered 34: 27-39.
183. Omer A, Ali M, Omer AH, Mustafa MD, Satir AA, et al. (1972) Incidence of G-6-PD deficiency and abnormal haemoglobins in the indigenous and immigrant tribes of the Sudan. Trop Geogr Med 24: 401-405.
184. Ondei LS, Silveira LM, Leite AA, Souza DR, Pinhel MA, et al. (2009) Lipid peroxidation and antioxidant capacity of G6PD-deficient patients with A-(202G>A) mutation. Genet Mol Res 8: 1345-1351.
185. Oudart JL, Tchernia G, Giscard R, Boal MR, Zucker JM, et al. (1971) [Erythrocyte glucose-6-phosphate dehydrogenase deficiency in African newborn infants in Dakar]. Afr J Med Sci 2: 87-100.
186. Padilla C (2011) Personal communication: unpublished data from the Philippines.
187. Palmarino R, Agostino R, Gloria F, Lucarelli P, Businco L, et al. (1975) Red cell acid phosphatase: another polymorphism correlated with Malaria? Am J Phys Anthropol 43: 177-186.
188. Pao M, Kulkarni A, Gupta V, Kaul S, Balan S (2005) Neonatal screening for glucose-6-phosphate dehydrogenase deficiency. Indian J Pediatr 72: 835-837.
189. Parsons IC, Ryan BPK (1962) Observations on Glucose-6-Phosphate Dehydrogenase Deficiency in Papuans. Med J Aust 2: 585-587.
190. Patel S (1977) Incidence of glucose-6-phosphate-dehydrogenase deficiency and correlation with some other laboratory findings among Tibetan refugees in Orissa. J Anthropol Soc Nip 85: 347-349.
191. Perine PL, Michael MT (1974) A preliminary survey for glucose-6-phosphate dehydrogenase deficiency and haemoglobin S in Ethiopia. Ethiop Med J 12: 179-184.
192. Plato CC, Cruz MT, Kurland LT (1964) Frequency of glucose-6-phosphate dehydrogenase deficiency red-green colour blindness and Xga blood-group among Chamorros. Nature 202: 728.
193. Plato CC, Rucknagel DL, Gershowitz H (1964) Studies on the distribution of glucose-6-phosphate dehydrogenase deficiency, thalassemia, and other genetic traits in the coastal and mountain villages of Cyprus. Am J Hum Genet 16: 267-283.
194. Prins HK, Loos JA, Meuwissen JH (1963) Glucose-6-phosphate dehydrogenase (G6pd) deficiency in West New Guinea. Trop Geogr Med 15: 361-370.
195. Ragab AH, el-Alfi OS, Abboud MA (1966) Incidence of glucose-6-phosphate dehydrogenase deficiency in Egypt. Am J Hum Genet 18: 21-25.
196. Rahimi Z, Raygani AV, Siabani S, Mozafari H, Nagel RL, et al. (2008) Prevalence of glucose-6-phosphate dehydrogenase deficiency among schoolboys in Kermanshah, Islamic Republic of Iran. East Mediterr Health J 14: 978-979.
197. Ramalho AS, Beiguelman B (1977) [Glucosephosphate dehydrogenase deficiency (G6-PD) in Brazilian blood donors]. AMB; Revista da Associação Médica Brasileira 23: 259-260.
198. Ratrisawadi V, Horpaopan S, Chotigeat U, Sangtawesin V, Kanjanapattanakul W, et al. (1999) Neonatal screening program in Rajavithi Hospital, Thailand. Southeast Asian J Trop Med Public Health 30 Suppl 2: 28-32.
199. Reclos GJ, Hatzidakis CJ, Schulpis KH (2000) Glucose-6-phosphate dehydrogenase deficiency neonatal screening: preliminary evidence that a high percentage of partially deficient female neonates are missed during routine screening. J Med Screen 7: 46-51.
200. Restrepo AM, Gutierrez E (1968) The frequency of glucose-6-phosphate dehydrogenase deficiency in Colombia. Am J Hum Genet 20: 82-85.
201. Reys L, Manso C, Stamatoyannopoulos G (1970) Genetic studies on southeastern Bantu of Mozambique. I. Variants of glucose-6-phosphate dehydrogenase. Am J Hum Genet 22: 203-215.
202. Richard F, Belhani M, Colonna P (1974) [G-6PD deficiency in newborns in Algiers (author's transl)]. Nouv Rev Fr Hematol 14: 453-459.
203. Ringelhann B, Dodu SRA, Konotey-Ahulu FID, Lehmann H (1968) A survey for haemoglobin variants, thalassaemia and glucose-6-phosphate dehydrogenase deficiency in Northern Ghana. Ghana Med J 7: 120-124.
204. Roberts DF, Triger DR, Morgan RJ (1970) Glucose-6-phosphate dehydrogenase deficiency and haemoglobin level in Jamaican children. West Indian Med J 19: 204-211.
205. Saha N, Bhattacharyya SP, Mukhopadhyay B, Bhattacharyya SK, Gupta R, et al. (1987) A genetic study among the Lepchas of the Darjeeling area of eastern India. Hum Hered 37: 113-121.
206. Saha N, Hong SH, Wong HA, Tay JS (1990) Red cell glucose-6-phosphate dehydrogenase phenotypes in several Mongoloid populations of eastern India: existence of a non-deficient fast variant in two Australasian tribes. Ann Hum Biol 17: 529-532.
207. Saha N, Ramzan M, Tay JS, Low PS, Basair JB, et al. (1994) Molecular characterisation of red cell glucose-6-phosphate dehydrogenase deficiency in north-west Pakistan. Hum Hered 44: 85-89.
208. Saha N, Tay JS (1990) Genetic studies among the Nagas and Hmars of eastern India. Am J Phys Anthropol 82: 101-112.
209. Saleem TH, Mendis BS, Osanyintuyi SO (1991) Glucose-6-phosphate dehydrogenase deficiency in a rural Saudi population. J Trop Med Hyg 94: 327-328.
210. Samuel AP, Saha N, Omer A, Hoffbrand AV (1981) Quantitative expression of G6PD activity of different phenotypes of G6PD and haemoglobin in a Sudanese population. Hum Hered 31: 110-115.
211. Sanchez MC, Villegas VE, Fonseca D (2008) [Glucose-6-phosphate dehydrogenase deficiency: enzimatic and molecular analysis in a Bogota population]. Colombia Medica 39: 14-23.
212. Sans M, Alvarez I, Bentancor N, Abilleira D, Bengochea M, et al. (1995) Blood protein genetic-markers in a northeastern Uruguayan population. Rev Bras Genet 18: 317-320.
213. Santana MS, de Lacerda MV, Barbosa MG, Alecrim WD, Alecrim MG (2009) Glucose-6-phosphate dehydrogenase deficiency in an endemic area for malaria in Manaus: a cross-sectional survey in the Brazilian Amazon. PLoS One 4: e5259.
214. Sarma DK, Shukla R, Lodha A, Abdulla A, Pataridze L (2006) Neonatal screening for glucose-6-phosphate dehydrogenase (G6PD) deficiency: Experience in a private hospital. Emirates Med J 24: 211-214.
215. Say B, Ozand P, Berkel I, Cevik N (1965) Erythrocyte glucose-6-phosphate dehydrogenase deficiency in Turkey. Acta Paediatr Scand 54: 319-324.
216. Schuurkamp GJ, Bhatia KK, Kereu RK, Bulungol PK (1989) Glucose-6-phosphate dehydrogenase deficiency and hereditary ovalocytosis in the Ok Tedi impact region of Papua New Guinea. Hum Biol 61: 387-406.
217. Segeja MD, Mmbando BP, Kamugisha ML, Akida JA, Savaeli ZX, et al. (2008) Prevalence of glucose-6-phosphate dehydrogenase deficiency and haemoglobin S in high and moderate malaria transmission areas of Muheza, north-eastern Tanzania. Tanzan J Health Res 10: 9-13.
218. Seth PK, Seth S (1971) Biogenetical studies of Nagas: glucose-6-phosphate dehydrogenase deficiency in Angami Nagas. Hum Biol 43: 557-561.
219. Sethuraman M, Rao KV (1978) A survey of glucose-6-phosphate dehydrogenase deficiency & sickle-cell trait on a local population of Tirupati. Indian J Exp Biol 16: 1098-1099.
220. Shah SS, Macharia A, Uyoga S, Williams TN (2011) Personal communication: unpublished data from Kenya.
221. Shimizu H, Tamam M, Soemantri A, Ishida T (2005) Glucose-6-phosphate dehydrogenase deficiency and Southeast Asian ovalocytosis in asymptomatic Plasmodium carriers in Sumba island, Indonesia. J Hum Genet 50: 420-424.
222. Singh H (1986) Glucose-6-phosphate dehydrogenase deficiency: a preventable cause of mental retardation. Br Med J (Clin Res Ed) 292: 397-398.
223. Siniscalco M, Bernini L, Filippi G, Latte B, Meera Khan P, et al. (1966) Population genetics of haemoglobin variants, thalassaemia and glucose-6-phosphate dehydrogenase deficiency, with particular reference to the malaria hypothesis. Bull World Health Organ 34: 379-393.
224. Sonnet J, Michaux JL (1960) Glucose-6-phosphate dehydrogenase deficiency, haptoglobin groups, blood groups and sickle cell trait in the Bantus of west Belgian Congo. Nature 188: 504-505.
225. Sözüöz A, Çamber I (1998) G6PD deficiency in Turkish Cypriots. Turk J Med Sci 28: 673-676.
226. Stamatoyannopoulos G, Fessas P (1964) Thalassaemia, glucose-6-phosphate dehydrogenase deficiency, sickling, and malarial endemicity in Greece: a study of Five Areas. Br Med J 1: 875-879.
227. Stamatoyannopoulos G, Panayotopoulos A, Motulsky AG (1966) The distribution of glucose-6-phosphate dehydrogenase deficiency in Greece. Am J Hum Genet 18: 296-308.
228. Suradi R, Monitja HE, Munthe BG, Suparno (1979) Glucose-6-phosphate dehydrogenase deficiency in the Dr. Cipto Mangunkusumo General Hospital. Paediatr Indones 19: 30-40.
229. Suryantoro P (2003) Glucose-6-phosphate dehydrogenase (G6PD) deficiency in Yogyakarta and its surrounding areas. Southeast Asian J Trop Med Public Health 34 Suppl 3: 138-139.
230. Sutton RN (1963) Erythrocyte glucose-6-phosphate-dehydrogenase deficiency in Trinidad. Lancet 1: 855.
231. Syahyuni R (2003) Hubungan defisiensi glucose-6-phosphate dehydrogenase (G-6-PD) degan kepadatan parasit malaria pada anak usia sekolah di daerah endemis malaria [Author translation: Glucose-6-phosphate defficiency during school children in malaria endemic area]. Semarang: Universitas Diponegoro.
232. Szathmary EJE, Cox DW, Gershowitz H, Rucknagel DL, Schanfield MS (1974) The Northern and Southeastern Ojibwa: serum proteins and red cell enzyme systems. Am J Phys Anthropol 40: 49-65.
233. Tagarelli A, Bastone L, Cittadella R, Calabro V, Bria M, et al. (1991) Glucose-6-phosphate dehydrogenase (G6PD) deficiency in southern Italy: a study on the population of the Cosenza province. Gene Geogr 5: 141-150.
234. Tagarelli A, Cittadella R, Bria M, Brancati C (1992) Glucose-6-phosphate dehydrogenase (G6PD) deficiency in the Albanian ethnic minority of Cosenza province, Italy. Gene Geogr 6: 71-78.
235. Talafih K, Hunaiti AA, Gharaibeh N, Gharaibeh M, Jaradat S (1996) The prevalence of hemoglobin S and glucose-6-phosphate dehydrogenase deficiency in Jordanian newborn. J Obstet Gynaecol Res 22: 417-420.
236. Taleb N, Loiselet J, Guorra F, Sfeir H (1964) [on glucose-6-phosphate dehydrogenase deficiency in autochthonous populations of Lebanon.]. C R Hebd Seances Acad Sci 258: 5749-5751.
237. Tantular IS, Iwai K, Lin K, Basuki S, Horie T, et al. (1999) Field trials of a rapid test for G6PD deficiency in combination with a rapid diagnosis of malaria. Trop Med Int Health 4: 245-250.
238. Tartaglia M, Scano G, DeStefano GF (1996) An anthropogenetic study on the Oromo and Amhara of central Ethiopia. Am J Hum Biol 8: 505-516.
239. Thakur A, Verma IC (1992) Interaction of malarial infection and glucose-6-phosphate dehydrogenase deficiency in Muria gonds of district Bastar, central India. Trop Geogr Med 44: 201-205.
240. Tills D, Warlow A, Lord JM, Suter D, Kopec AC, et al. (1983) Genetic factors in the population of Plati, Greece. Am J Phys Anthropol 61: 145-156.
241. Tsoneva M, Proinova N, Mavrudieva M (1974) [Incidence of glucose-6-phosphate dehydrogenase deficiency in blood donors of Sofia]. Vutr Boles 13: 46-51.
242. Tuchinda S, Rucknagel DL, Na-Nakorn S, Wasi P (1968) The Thai variant and the distribution of alleles of 6-phosphogluconate dehydrogenase and the distribution of glucose 6-phosphate dehydrogenase deficiency in Thailand. Biochem Genet 2: 253-264.
243. Tuda JSB, Kepel BJ, Nakatsu M, Matsuoka H (2007) Prevalensi defisiensi Glucose-6-Phosphate Dehydrogenase (G6PD) pada anak Sekolah Dasar yang tinggal di daerah endemis malaria di Sulawesi utara. Jurnal Kedokteran Yarsi 15: 59-63.
244. Turan Y (2006) Prevalence of erythrocyte glucose-6-phosphate dehydrogenase (G6PD) deficiency in the population of western Turkey. Arch Med Res 37: 880-882.
245. Usanga EA, Ameen R (2000) Glucose-6-phosphate dehydrogenase deficiency in Kuwait, Syria, Egypt, Iran, Jordan and Lebanon. Hum Hered 50: 158-161.
246. Voronov AA, Krasilnikov AA (1973) [Population study of glucose-6-phosphate deficiency in the Transcaucasus]. Probl Gematol Pereliv Krovi 18: 21-23.
247. Walter H, Neumann S, Nemeskeri J (1968) Investigations on the occurrence of glucose-6-phosphate-dehydrogenase deficiency in Hungary. Acta Genet Stat Med 18: 1-11.
248. Weimer TA, Salzano FM, Hutz MH (1981) Erythrocyte isozymes and hemoglobin types in a southern Brazilian population. J Hum Evol 10: 319-328.
249. Welch SG, Lee J, McGregor IA, Williams K (1978) Red cell glucose 6 phosphate dehydrogenase genotypes of the population of two West African villages. Hum Genet 43: 315-320.
250. White JM, Byrne M, Richards R, Buchanan T, Katsoulis E, et al. (1986) Red cell genetic abnormalities in Peninsular Arabs: sickle haemoglobin, G6PD deficiency, and alpha and beta thalassaemia. J Med Genet 23: 245-251.
251. Willcox M, Bjorkman A, Brohult J (1983) Falciparum malaria and beta-thalassaemia trait in northern Liberia. Ann Trop Med Parasitol 77: 335-347.
252. Woodfield DG, Scragg RFR, Blake NM (1974) Distribution of blood, serum protein and enzyme groups among the Fuyuge speakers of the Goilala sub district. Hum Hered 24: 507-519.
253. Wu CX, Shan KR, He Y, Qi XL, Li Y, et al. (2007) Detection of glucose-6-phosphate dehydrogenase gene mutations of Tujia ehtnic in Jiangkou, Guizhou. Chinese Journal of Endemiology 26: 415-417.
254. Xiu J, Qi XL, Shan KR, Xie Y, He Y, et al. (2005) [G6PD gene mutations in Shui people in Sandu of Guizhou]. Zhongguo Shi Yan Xue Ye Xue Za Zhi 13: 147-150.
255. Yamamoto T, Amano H, Sano A, Takahash.Y, Takahash.H (1974) Study about glucose-6-phosphate-dehydrogenase deficiency in Laos. Jpn J Hum Genet 19: 64-64.
256. Yenchitsomanus P, Summers KM, Chockkalingam C, Board PG (1986) Characterization of G6PD deficiency and thalassaemia in Papua New Guinea. P N G Med J 29: 53-58.
257. Young GP, Smith MB, Woodfield DG (1974) Glucose-6-phosphate dehydrogenase deficiency in Papua New Guinea using a simple methylene blue reduction test. Med J Aust 1: 876-878.
258. Yudhaputri FA, Baird JK, Nixon C (2011) Personal communication: unpublished data from Indonesia.
259. Yue PC, Strickland M (1965) Glucose-6-phosphate-dehydrogenase deficiency and neonatal jaundice in Chinese male infants in Hong Kong. Lancet 1: 350-351.
260. Zaidman JL, Leiba H, Scharf S, Steinman I (1976) Red cell glucose-6-phosphate dehydrogenase deficiency in ethnic groups in Israel. Clin Genet 9: 131-133.
261. Zannos-Mariolea L, Kattamis C (1961) Glucose-6-phosphate dehydrogenase deficiency in Greece. Blood 18: 34-47.
