# Supplementary material for: G6PD Deficiency Prevalence and Estimates of Affected Populations in Malaria Endemic Countries: A Geostatistical Model-Based Map
Source: PLoS Med. 2012 Nov 13;9(11):e1001339. doi: 10.1371/journal.pmed.1001339 (PMC3496665; doi:10.1371/journal.pmed.1001339)
Supplement: Protocol S1 — Assembling a global database of G6PD deficiency (G6PDd) prevalence surveys. (S1.1) Overview of database requirements. (S1.2) Library assembly. (S1.3) Dataset inclusion criteria. (S1.4) Survey diagnostic methods. (S1.5) The final G6PDd survey dataset. (S1.6) Defining MECs' limits. (DOCX) [file pmed.1001339.s002.docx]

**Protocol S1. Assembling a global database of G6PD deficiency (G6PDd) prevalence surveys**

**S1.1 Overview of database requirements**

This document summarises the methodological steps involved in assembling the input dataset for the mapping model, documenting both the assembly of sources and data abstraction of survey details into a customised database. The aim of this literature search was to assemble a database of G6PD deficiency (G6PDd) surveys which would form the evidence base for the geostatistical mapping model (Protocol S2). A similar search strategy and data abstraction protocol has been previously described in detail in reference to the Duffy blood group variants by Howes et al. [[1](#_ENREF_1)] and malaria parasite rate data by Guerra et al. [[2](#_ENREF_2)]. The final input dataset is available for download from: <http://www.map.ox.ac.uk/>.

**S1.2 Library assembly**

Extensive efforts were invested in attempting to assemble all available surveys of G6PDd. These came both from the published and unpublished literature, dating from 1959. Systematic searches of the online biomedical literature databases PubMed (http://www.pubmed.gov), ISI Web of Science (http://wok.mimas.ac.uk/) and Scopus (http://www.scopus.com) were conducted for all articles including the terms ‘G6PD’, ‘glucose-6-phosphate dehydrogenase’ and ‘glucose 6 phosphate dehydrogenase’. Following duplicate removal, a total of 17,272 unique sources were found to contain these terms. Titles and abstracts were reviewed conservatively for relevance to the project and, for example, clinical case reports, laboratory studies and animal studies were excluded. Searches were then conducted to identify full-text articles of the potentially relevant sources.

Unpublished sources were also identified through contact with the research and medical communities. In particular, the Filipino Newborn Screening Reference Center (NIH, Philippines) contributed their universal screening results since 2004 to this study, adding 636 locations to the database. Individuals who shared data with the project are gratefully acknowledged on the Malaria Atlas Project (MAP) website (<http://www.map.ox.ac.uk/inherited-blood-disorders/acknowledgements/>).

**S1.3 Dataset inclusion criteria**

All sources for which full text copies could be identified were reviewed in detail. Specific inclusion criteria for the final dataset are detailed below. A schematic breakdown of these criteria is illustrated in Figure S1.1.

**Figure S1.1.** **Breakdown of the exclusions applied in assembling the input dataset.** Sources include published journal articles, book chapters, published and unpublished reports, etc; each source may report multiple surveys. Orange rectangles correspond to review steps; blue rectangles show the number of sources or surveys at each level of exclusion; pale blue indicating when the decisions are based on full-text review. Diamonds indicate the number of remaining surveys following each exclusion. The final input dataset, separated by sex, is represented by parallelograms.

^†^Most of the surveys reporting these methods had already been excluded before this step; this is not a representative estimate of the number of surveys available using those diagnostic methods.

A comprehensive protocol was developed to ensure objectivity and consistency in the abstraction decision-making process across the data-abstraction team [MMH, REH, OAN, FBP]. All data entered into the database were checked by a second individual and then reviewed by a senior member of the team [REH or FBP] before inclusion in the model input dataset.

Sex

To account for the G6PD gene’s position on the X-chromosome and hence its sex-specific inheritance patterns [[3](#_ENREF_3)], rates of deficiency were recorded separately for males and females. Data were then considered separately by the model (Protocol S2). Surveys which did not report sex-specific data were excluded.

Representativeness

To generate a map showing the overall population prevalence of G6PDd, only surveys of representative population samples were included. Surveys preferentially selecting individuals from specific ethnic groups were excluded as these would provide incomplete, and potentially skewed, information about the overall local frequency of G6PDd. Furthermore, samples of patients, both with minor ailments and all hospitalised cases, including malaria patients, were excluded for the same reasons of being potential biased samples.

Spatial specificity

Prerequisite to inclusion in the database is that surveys be geographically specific and possible to map. Both the latitude and longitude of all surveys, as well as the survey’s spatial extent, were recorded. A specific mapping protocol has been developed within the Malaria Atlas Project (MAP) to ensure that a maximum of surveys can be positioned with the required specificity [[1](#_ENREF_1),[2](#_ENREF_2)]. This involved both the use of online geopositioning gazetteers and direct contact with authors where online searches failed. Wherever possible, surveys were mapped to their specific recruitment sites. However, surveys were often reported to administrative level, rather than village or city levels; administrative level 1 corresponds to provinces or states, and administrative level 2 to districts. Globally, the sizes of these administrative divisions are highly variable. Previous MAP databases have used administrative region levels as a cut-off for inclusion: for instance allowing administrative level 2 data, but not administrative level 1 or national-level data. To refine the specificity of the data used here, the G6PD database included the spatial extent of all data points alongside geographic coordinates. We used ArcGIS Desktop (ArcMap 10.0, ESRI Inc., Redlands, CA, USA) to digitise and calculate surface areas of spatial polygons. Where data presented were amalgamated from multiple sites, the maximum distance between sites was measured in Encarta (Microsoft Corporation, Redmond, WA, USA), and the area of the circle around these was calculated to obtain a conservative estimate of the maximal area represented. A conservative cut-off, based on the average size of the administrative level 2 regions, was used: 3,867 km^2^. All surveys with an extent larger than this were excluded from the database as these were considered too spatially unspecific. This meant that national-level data from ten countries could be included; these were mostly Pacific islands, but also Bahrain and Singapore.

Only a single survey estimate could be entered for each pixel on the global grid. All spatial duplicates were therefore identified and only the survey considered to be most representative of the contemporary status of G6PD deficiency was included. In selecting between spatially duplicated surveys, the factors considered were survey date, sample size, whether both sexes had been tested and the diagnostic methodology. Beutler’s SPOT test [[4](#_ENREF_4)] and electrophoretic methods [[5](#_ENREF_5)] were considered more reliable than Motulsky’s cresyl blue dye decolourisation test [[6](#_ENREF_6)], for example. Spatial overlap was notable in the Philippines, due to inclusion of the extensively distributed national screening data. To overcome this, all opportunistic community surveys from the Philippines (n = 27) were excluded, leaving only the gold-standard universal screening data.

**S1.4 Survey diagnostic methods**

The International Committee for Standardization in Haematology recommends the fluorescent spot test for population screening surveys [[7](#_ENREF_7)]. However, this method requires some technical equipment and numerous alternative methods are also in wide use to overcome the constraints of the spot test. There is no single, standardised method in use today. Furthermore, the methods used are invariably modified by users (for example, in terms of the cut-off times imposed) and adapted to local conditions and survey constraints; there is therefore a broad range of methodology employed in diagnosing G6PDd. The database assembled here recorded key information about the methodology used, and these were categorised into ten methods (Figure S1.2). Where multiple methods were used, data from the initial screening method used to test the whole population was recorded; if several methods tested the full population (rare), then the method deemed most reliable was used.

Phenotypic vs. molecular diagnosis

The central dichotomy within diagnostics lies between molecular and phenotypic tests. As the objective of this study was to represent clinically significant cases, these were best detected by assessment of residual enzyme activity levels. In contrast, molecular methods look for specific mutations, and their relationship with the expressed enzyme activity level, and thus their clinical severity, is poorly established in most cases [[8](#_ENREF_8)]. A further limitation to molecular methods stems from the numerous mutations in the G6PD gene (186 mutations catalogued by Minucci et al. in 2012 [[9](#_ENREF_9)]); most molecular methods search for only a subset of these, and hence cannot reliably identify all deficiency cases; significant discrepancies in this regard were recently demonstrated by a comparative study of enzymatic and molecular methods by Johnson et al. [[10](#_ENREF_10)]. Surveys diagnosed with only molecular methods were therefore excluded from the current mapping analysis.

**Diagnostic methods reported in the G6PDd database (n = 1,734)**

**Figure S1.2. Diagnostic methodology use across the dataset.** *EAA*: enzyme activity assay (quantitative or semi-quantitative measure of NAPD>NADPH conversion) (47%); *BCB*: brilliant cresyl blue test (14%); *MRT*: methaemoglobin reduction test (12%), *SPOT*: NADPH fluorescent spot test (9%), *DPIP*: 2,6-dicholorophenol indophenol dye test (6%), *ELE*: enzyme electrophoresis (5%), *WST-8*: WST-8/1-methoxy PMS method (3%); *MTT-PMS*: 3-(4,5-dimethyl-2-thiazolyl)-2,5-diphenyl-2H tetrazolium bromide (MTT)-PMS (1%); NBT: nitro blue tetrazolium test (<1%); MBR: methylene blue reduction test (<1%). In 2% of surveys, the diagnostic method was not reported or was unclear from the author description.

Diagnosing deficiency

A number of simple, binary, qualitative/semi-quantitative/quantitative diagnostic kits were widely recorded across our database, with standardised protocols for many of these originally set by the WHO [[11](#_ENREF_11)], which are all considered adequate for diagnosing deficiency in males [[5](#_ENREF_5),[12](#_ENREF_12)]. Most of these kits assess the rate of NAPD reduction to NADPH through dye decolourisation or fluorescence, which informs G6PD enzyme activity. Sensitivity and specificity of some of the recommended methods has been previously examined and were found to be very high for males [[13](#_ENREF_13),[14](#_ENREF_14)]. Applying the principle of dye intensity to electrophoretic gels, detailed diagnoses of particular variants as well as residual enzyme activity can be ascertained. Enzyme electrophoresis is therefore another reliable diagnostic. Exceptions to this are the Heinz body test and the glutathione stability tests, which are indirect, proxy measures of G6PD activity and were not considered to be reliable indicators [[12](#_ENREF_12)]. Thus all surveys using these tests were excluded. Enzyme activity levels are the same for homozygous deficient females as they are in deficient males – all red blood cells in a homozygous deficient female are affected by the deficiency. Thus diagnosis has been demonstrated to be equally reliable in homozygotes and deficient males.

Heterozygous deficient females are harder to diagnose reliably due to their mosaic populations of deficient and wild-type red blood cells. The relative proportions of each cell-type population are variable and a given genotype may be expressed as a spectrum of phenotypic levels of deficiency. As a result, the diagnostic cut-off point may therefore influence the proportion of females found to be deficient in a survey. This is discussed in further detail in Protocol S5. All model predictions made from these input datasets, however, must be evaluated with these constraints and uncertainties in mind.

Conclusions

With the exception of Heinz body diagnoses, GSH stability tests and molecular analyses, all diagnostic methods were included in the study. Diagnostic outcome is influenced by a number of factors, including variable sensitivity of methods used, variable reaction time cut-offs used by investigators, and differing levels of residual activity associated with the numerous genetic variants. A further confounding factor for diagnosis which has not yet been discussed, is the increased probability of false-positive diagnoses due to anaemia, which reduces the overall number of red blood cells, and therefore level of G6PD enzyme per volume of blood; G6PD diagnosis should account for this [[15](#_ENREF_15)], though rarely does in most community screening surveys.

While the influence of these issues has, on the whole, been deemed relatively acceptable for diagnosing males and homozygotes, who tend to have a distinct deficient/non-deficient status, diagnosing heterozygotes across a spectrum of residual activity levels is less clear-cut. However, the absence of a single standardised methodological rapid-test suited to mass surveying, as well as the poor understanding of the genotype-phenotype relationship in


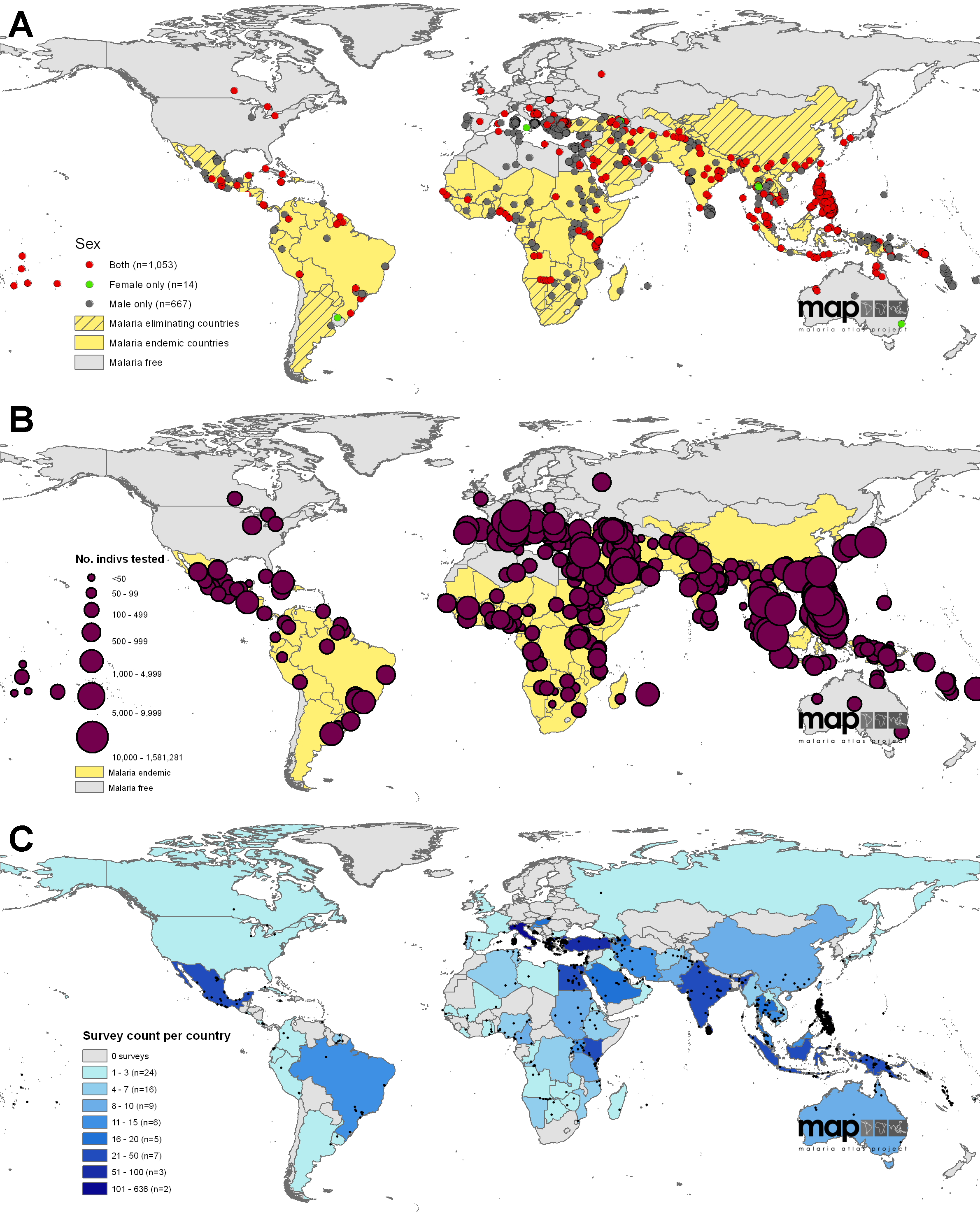


**Figure S1.3. Global distribution of the assembled G6PDd dataset.** Panel A shows the distribution of surveys according to which sex was tested: red where both sexes are included, green if female data only and black of male data only; Panel B maps the total numbers of individuals (both sexes) sampled in each survey; Panel C indicates the number of surveys identified from each country; Panel D maps the spatial extent of each of the surveys. Background map colours in Panels A, B and D represent the status of the national malaria programme (malaria free or malaria endemic).


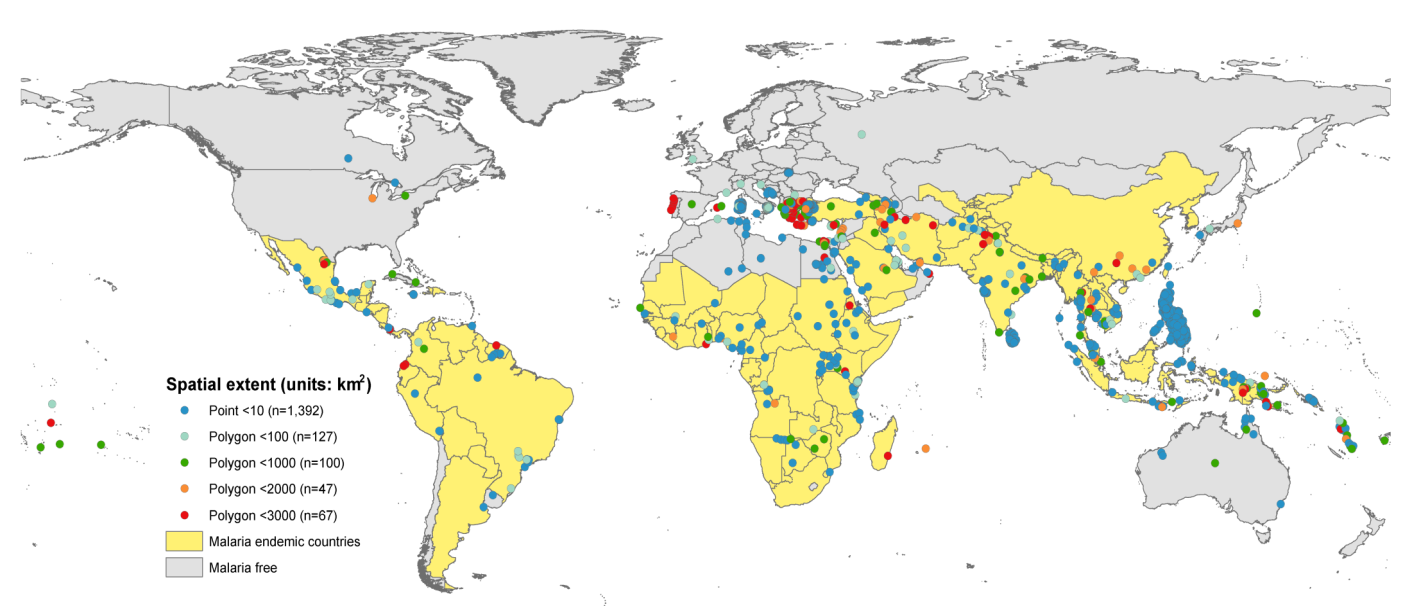

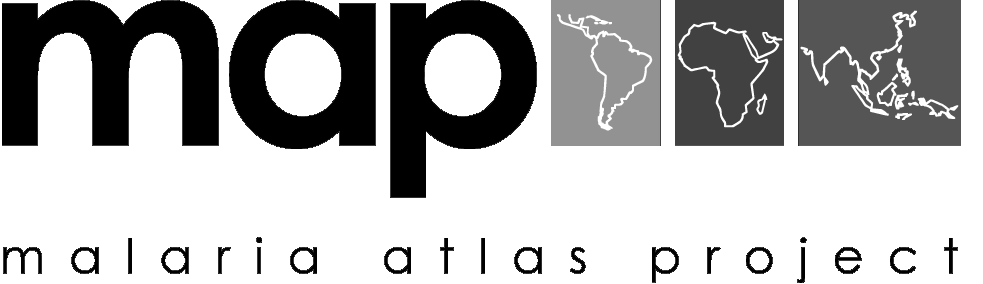


**D**

heterozygotes and of the association with haemolytic risk, there is limited potential for imposing adjustments to the input dataset. The raw survey data was therefore used, with female data being treated separately from the more reliable male data. The map aims to represent individuals at greatest risk of G6PD-associated haemolysis. While some discrepancy risks being introduced due to the difficulties of diagnosing this complex system, this will only affect a small proportion of borderline heterozygote individuals, with intermediate deficiency, thus individuals who are not necessarily at greatest risk of deficiency. In the absence of any standardised methodology for this, we made the assumption that phenotypic diagnostic test cut-offs would have been calibrated to detect locally significant levels of G6PDd and that individuals at risk of primaquine-associated haemolysis (male and female) would be included in this group.

**S1.5 The final G6PDd survey dataset**

As detailed in Figure S1.1, the original set of references included 17,272 sources from online database searches and 472 sources from existing databases and personal communications. From these, 1,734 spatially-unique surveys were identified which met all our inclusion criteria, 1,289 of which were in malaria endemic countries (MECs) (74%); 97 countries, including 55 MECs, are represented in the dataset. These surveys were reported from 261 sources, which are listed in the Supplementary References. Table S1 summarises this final set of studies used to inform the mapping model analysis.

Surveying effort, in terms of number of surveys and number of individuals tested is strongly biased towards Asia & Europe, with 85% of surveys and 98.6% of individuals sampled in the Eurasian region. Surveys were tightly clustered across Sardinia, southern

|  |  | **Africa** | | **Americas** | | **Asia & Europe** | | **Global** | |
| --- | --- | --- | --- | --- | --- | --- | --- | --- | --- |
| **Total surveys** | | **Total** | ***MEC*** | **Total** | ***MEC*** | **Total** | ***MEC*** | **Total** | ***MEC*** |
|  |  | 169 | *132* | 67 | *56* | 1,498 | *1,101* | 1,734 | *1,289* |
|  | Number of countries | 28 | *23* | 15 | *9* | 54 | *23* | 97 | *55* |
| **Publication time** | |  |  |  |  |  |  |  |  |
|  | 1959 - 1969 | 42 | *31* | 22 | *19* | 204 | *50* | 268 | *100* |
|  | 1970 - 1979 | 41 | *29* | 14 | *9* | 77 | *59* | 132 | *97* |
|  | 1980 - 1989 | 21 | *16* | 8 | *6* | 143 | *110* | 172 | *132* |
|  | 1990 - 1999 | 12 | *3* | 12 | *11* | 224 | *60* | 248 | *74* |
|  | 2000 - 2011 | 53 | *53* | 11 | *11* | 850 | *822* | 914 | *886* |
|  |  |  |  |  |  |  |  |  |  |
| **Spatial extent (all surveys are ≤3,867km^2^)** | | | |  |  |  |  |  |  |
|  | Admin 0 centroids | 1 | *0* | 0 | *0* | 9 | *0* | 10 | *0* |
|  | Admin 1 centroids | 8 | *2* | 0 | *0* | 16 | *4* | 24 | *6* |
|  | Admin 2 centroids | 3 | *3* | 9 | *8* | 62 | *17* | 74 | *28* |
|  | Admin 3 centroids | 0 | *0* | 0 | *0* | 8 | *8* | 8 | *8* |
|  | Polygons (>25km^2^ to ≤3,867km^2^) | 55 | *52* | 13 | *9* | 105 | *51* | 173 | *112* |
|  | Wide areas (>10km^2^ to ≤25km^2^) | 9 | *6* | 8 | *8* | 13 | *8* | 30 | *22* |
|  | Points (≤10km^2^) | 88 | *64* | 36 | *30* | 1,265 | *999* | 1,389 | *1,093* |
|  | Multiple points | 5 | *5* | 1 | *1* | 20 | *14* | 26 | *20* |
|  |  |  |  |  |  |  |  |  |  |
| **Data type** | |  |  |  |  |  |  |  |  |
|  | Male only | 88 | *56* | 33 | *30* | 546 | *225* | 667 | *311* |
|  | Female only | 0 | *0* | 1 | *0* | 13 | *11* | 14 | *11* |
|  | Male & Female | 81 | *76* | 33 | *26* | 939 | *865* | 1,053 | *967* |
|  |  |  |  |  |  |  |  |  |  |
| **Total individuals sampled** | |  |  |  |  |  |  |  |  |
|  | Male | 24,528 | *13,777* | 24,979 | *20,414* | 2,372,541 | *2,189,795* | 2,422,048 | *2,223,986* |
|  | Female | 6,859 | *5,172* | 5,028 | *3,374* | 2,017,773 | *1,973,873* | 2,029,660 | *1,982,419* |
|  | Total | 31,387 | *18,949* | 30,007 | *23,788* | 4,390,314 | *4,163,668* | 4,451,708 | *4,206,405* |
|  |  |  |  |  |  |  |  |  |  |
| **Survey count by sample size (male + female)** | | | |  |  |  |  |  |  |
|  | <50 | 57 | *44* | 7 | *7* | 258 | *157* | 322 | *208* |
|  | 50 - <100 | 36 | *31* | 13 | *12* | 307 | *201* | 356 | *244* |
|  | 100 - <500 | 63 | *49* | 33 | *26* | 495 | *355* | 591 | *430* |
|  | 500 - <1,000 | 9 | *7* | 9 | *7* | 126 | *109* | 144 | *123* |
|  | 1,000 - <5,000 | 4 | *1* | 5 | *4* | 195 | *171* | 204 | *176* |
|  | 5,000 - <10,000 | 0 | *0* | 0 | *0* | 49 | *45* | 49 | *45* |
|  | >10,000 | 0 | *0* | 0 | *0* | 68 | *63* | 68 | *63* |
|  |  |  |  |  |  |  |  |  |  |
|  | Mean | 185.7 | *143.6* | 447.9 | *424.8* | 2,930.8 | *3,782.0* | 2,567.0 | *3,263.3* |
|  | Median | 83.0 | *77.0* | 147.0 | *119.5* | 165.0 | *231.0* | 145.0 | *197.5* |
|  | IQR | 43 - 160 | *45 - 150* | 90 - 479 | *85 - 419* | 70 - 662 | *80 - 1009* | 66 - 551 | *77 - 759* |
|  |  |  |  |  |  |  |  |  |  |
| **G6PD deficiency prevalence (male & female data)** | | | |  |  |  |  |  |  |
|  | Surveys with no G6PDd | 22 | *15* | 23 | *20* | 212 | *98* | 257 | *133* |
|  | Surveys with G6PDd | 147 | *118* | 44 | *36* | 1,286 | *1,003* | 1,477 | *1,156* |

**Table S1. Summary of G6PD dataset characteristics by region.** Numbers correspond to spatially unique community surveys meeting the study inclusion criteria and used in the mapping analysis. Italicised terms refer to the numbers within MECs.

mainland Italy, western Turkey, Sri Lanka, and especially the Philippines from where national screening data was available. Their distribution, datatype in terms of sex, sample size and prevalence values are shown in Figure S1.3. All three aspects influence each survey’s relative influence on the model predictions (Protocol S2). Total numbers of males and females surveyed were roughly equal (2.4 million males and 2.0 million females). The distribution of these surveys through time is displayed in Figure S1.4.

A range of diagnostic methods were used to determine G6PD status, with the most common being enzyme activity assays reported in 47% of surveys (quantitative or semi-quantitative assessment of NAPD>NADPH breakdown [[12](#_ENREF_12)], this method is used in the Philippines, which made up 36% of the dataset); other diagnoses were qualitative or semi-quantitative: Motulsky and Campbell-Kraut’s [[6](#_ENREF_6)] brilliant cresyl blue dye test (14% surveys globally); Brewer’s [[16](#_ENREF_16)] methaemoglobin reduction test (12% surveys globally); Beutler’s [[4](#_ENREF_4)] NADPH fluorescent spot test (9%); and Bernstein’s [[17](#_ENREF_17)] DPIP method (6% surveys globally) (Figure S1.2)


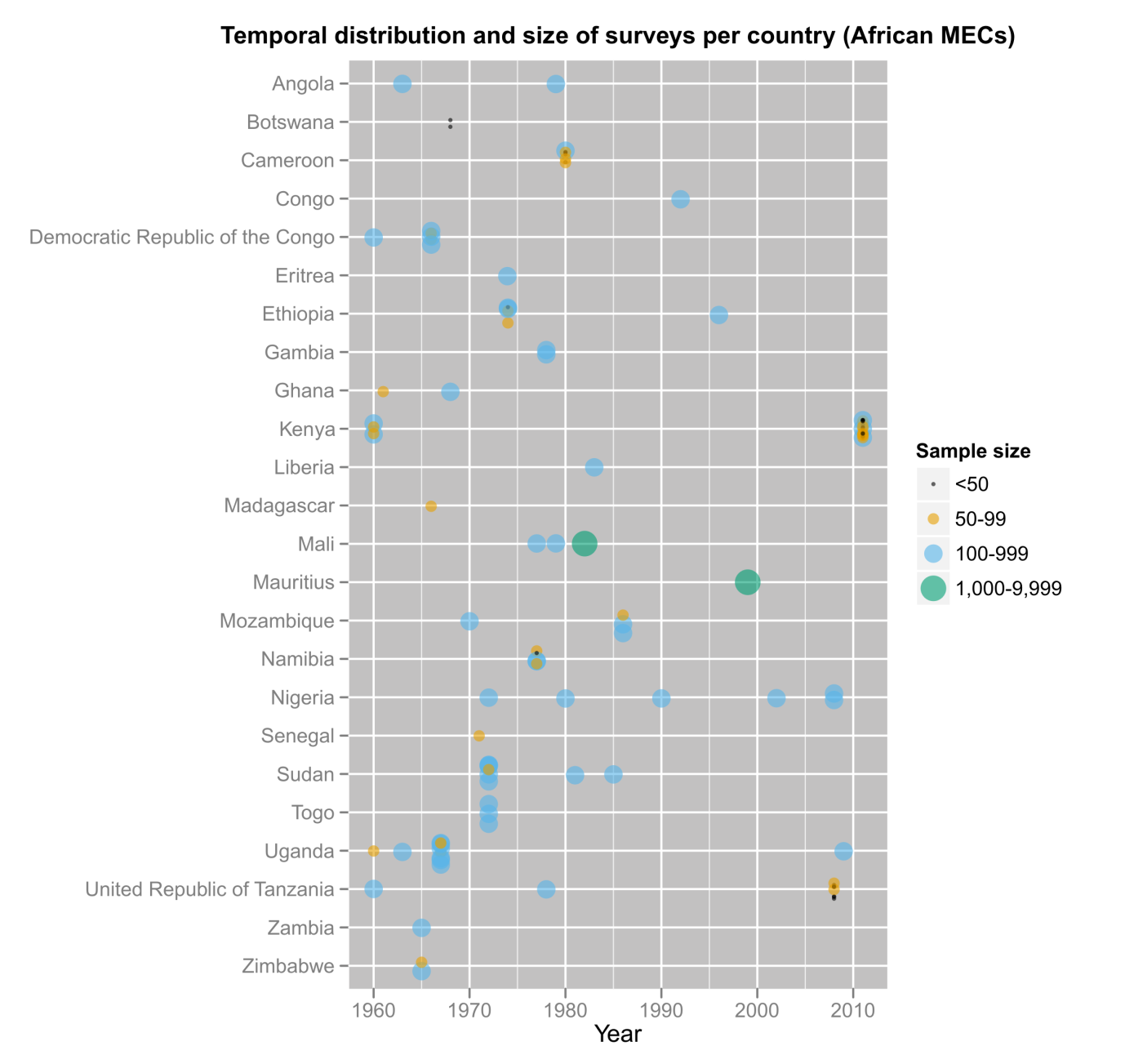


**A**


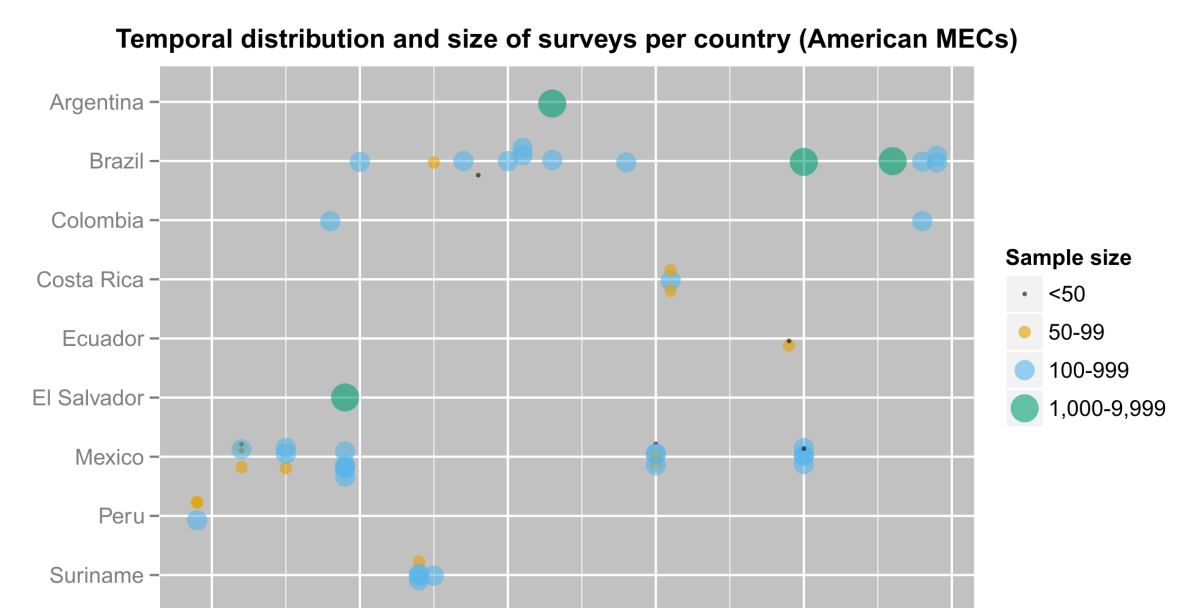


**B**

**Figure S1.4. The temporal distribution of surveys by country,** in African MECs (Panel A), MECs of the Americas (Panel B) and of Europe and Asia (Panel C). Data point colour and size reflect the total number of individuals tested in each survey. The date corresponds to the date of publication, as survey dates were not consistently reported.


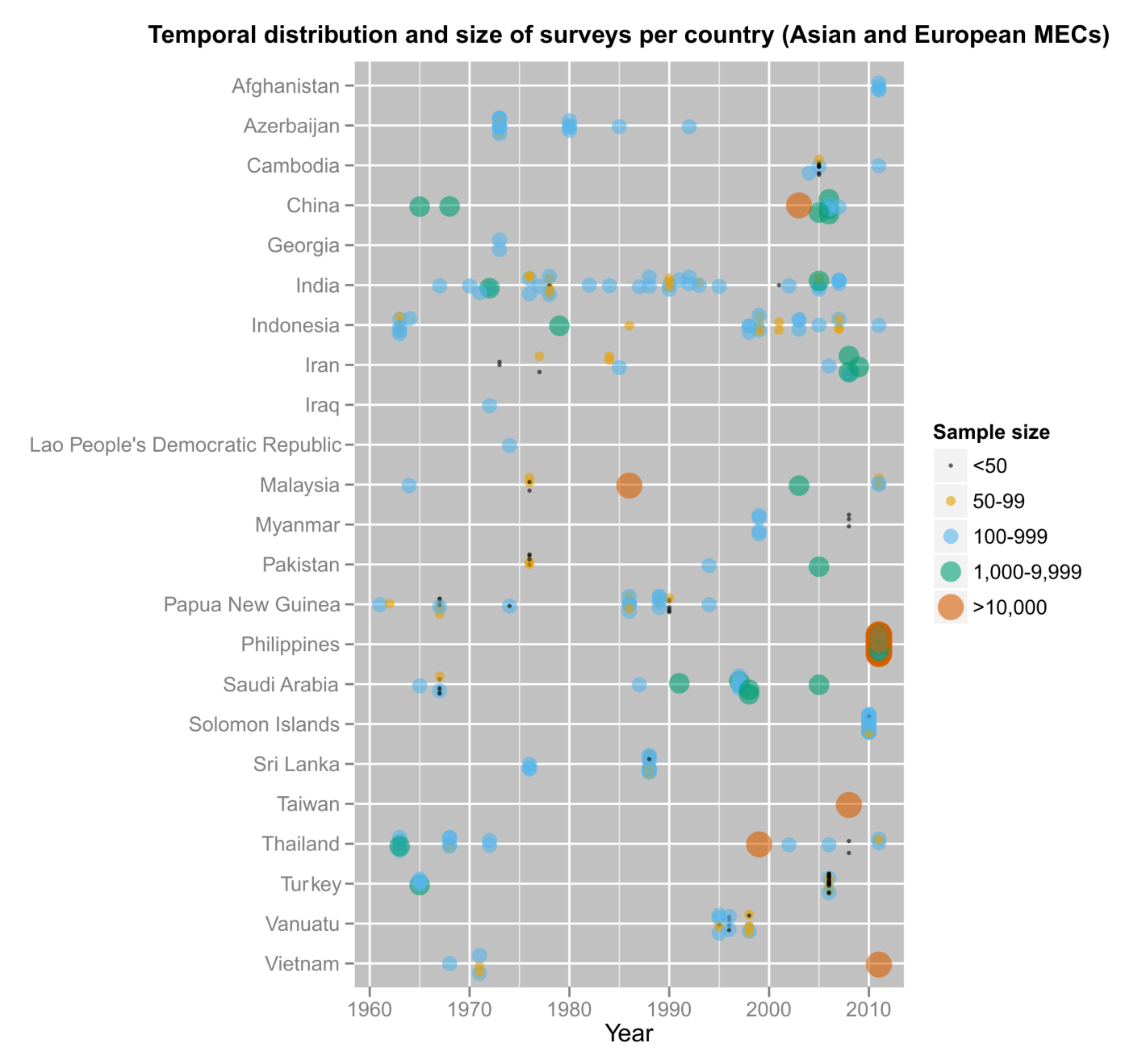


**CC**

**S1.6 Defining Malaria Endemic Countries (MECs) limits**

In considering the risk that primaquine carries for G6PDd individuals, this study is concerned with the condition’s prevalence within areas of malaria endemicity. Outside these regions, primaquine will not be used routinely, and is most likely to be prescribed from hospitals, where facilities for G6PD activity testing are more likely to be available. The public health interest in this map is anticipated to be greatest in areas where point-of-care testing is unavailable. Furthermore, few G6PD surveys were available outside historically malaria endemic regions (including current MECs and the Mediterranean regions), which would have made predictions for these data-sparse regions much less reliable.

At the low end of transmission, classifying a country’s malaria endemic status can be difficult and contentious [[18](#_ENREF_18)], and is ultimately dependent upon meeting the administrative WHO certification criteria. The G6PDd map presented here is most applicable to countries with large-scale programmes, rather than targeted individual hospital-based treatment, as is likely to be the case in countries where malaria is very rare. The MAP’s *Plasmodium* transmission mapping initiatives [[19-21](#_ENREF_19)] do not consider countries with very low endemicity where transmission is limited to small, sporadic foci stimulated by imported cases (including Algeria, Armenia, Egypt, Jamaica, Russia and Syria), to be usefully included as endemic in mapping terms. The MAP therefore considers 99 MECs [[21](#_ENREF_21)] (including Mayotte, a French overseas department). We focus this G6PDd mapping effort to the MAP MEC limits where large-scale primaquine use is still appropriate and point-of-care G6PDd testing likely to be rare. Countries targeting national elimination were identified from the 2011 Atlas of Malaria-Eliminating Countries [[22](#_ENREF_22)] and included 35 of the MECs considered in this study (Figure S1.5).

**
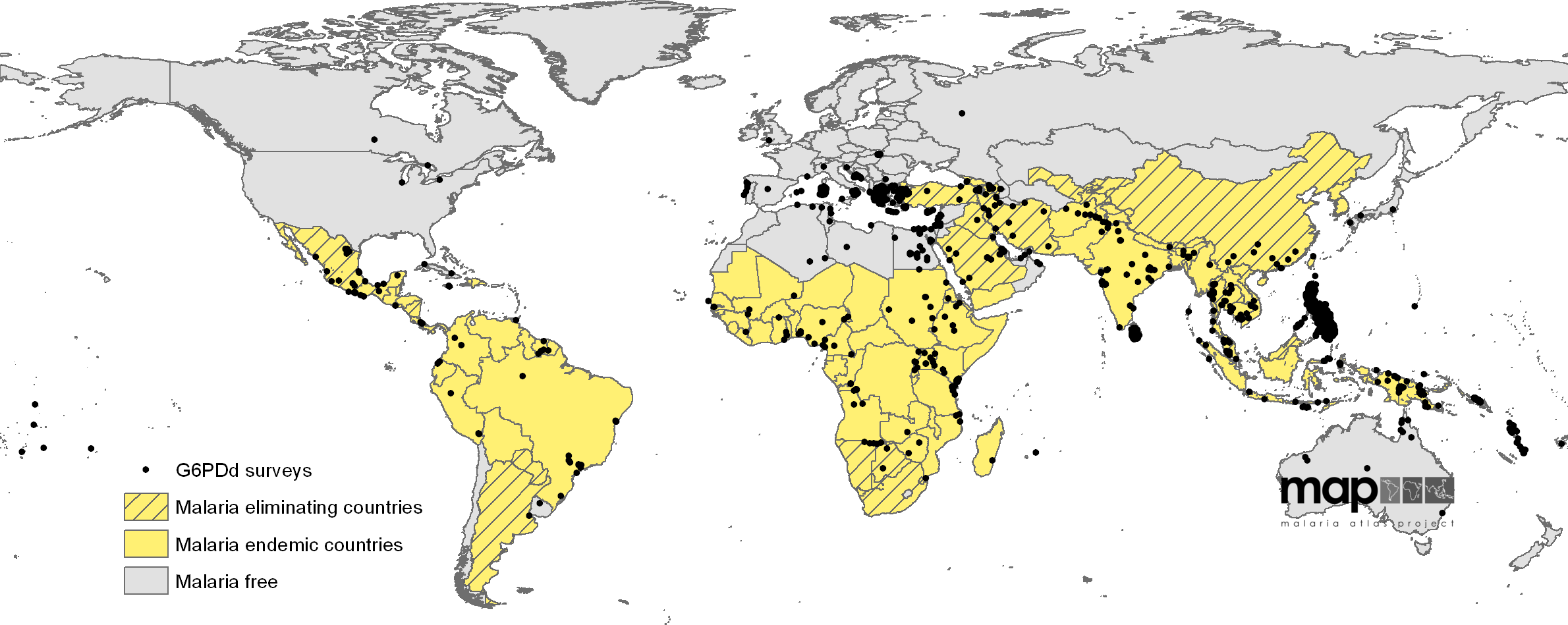
**

**Figure S1.5. Malaria endemic country limits used for mapping G6PDd.** Grey areas denote malaria-free regions; yellow areas are malaria endemic (n=99 countries), with hatches indicating those countries targeting malaria elimination (n=35) [[22](#_ENREF_22)].

**References**

1. Howes RE, Patil AP, Piel FB, Nyangiri OA, Kabaria CW, et al. (2011) The global distribution of the Duffy blood group. Nat Commun 2: 266.

2. Guerra CA, Hay SI, Lucioparedes LS, Gikandi PW, Tatem AJ, et al. (2007) Assembling a global database of malaria parasite prevalence for the Malaria Atlas Project. Malar J 6: 17.

3. Cappellini MD, Fiorelli G (2008) Glucose-6-phosphate dehydrogenase deficiency. Lancet 371: 64-74.

4. Beutler E, Mitchell M (1968) Special modifications of the fluorescent screening method for glucose-6-phosphate dehydrogenase deficiency. Blood 32: 816-818.

5. WHO Working Group (1989) Glucose-6-phosphate dehydrogenase deficiency. Bull World Health Organ 67: 601-611.

6. Motulsky AG, Campbell-Kraut JM. Population genetics of glucose-6-phosphate dehydrogenase deficiency of the red cell. In: Blumberg BS, editor; 1961; New York, NY. Grune & Stratton. pp. 159.

7. Beutler E, Blume KG, Kaplan JC, Lohr GW, Ramot B, et al. (1979) International Committee for Standardization in Haematology: recommended screening test for glucose-6-phosphate dehydrogenase (G-6-PD) deficiency. Br J Haematol 43: 465-467.

8. Baird JK, Surjadjaja C (2011) Consideration of ethics in primaquine therapy against malaria transmission. Trends Parasitol 27: 11-16.

9. Minucci A, Moradkhani K, Hwang MJ, Zuppi C, Giardina B, et al. (2012) Glucose-6-phosphate dehydrogenase (G6PD) mutations database: review of the "old" and update of the new mutations. Blood Cells Mol Dis 48: 154-165.

10. Johnson MK, Clark TD, Njama-Meya D, Rosenthal PJ, Parikh S (2009) Impact of the method of G6PD deficiency assessment on genetic association studies of malaria susceptibility. PLoS One 4: e7246.

11. Betke K, Brewer GJ, Kirkman HN, Luzzatto L, Motulsky AG, et al. (1967) Standardization of procedures for the study of glucose-6-phosphate dehydrogenase. Report of a WHO Scientific Group. World Health Organ Tech Rep Ser No. 366: 1-53.

12. Beutler E (1994) G6PD deficiency. Blood 84: 3613-3636.

13. Peters AL, Van Noorden CJ (2009) Glucose-6-phosphate dehydrogenase deficiency and malaria: cytochemical detection of heterozygous G6PD deficiency in women. J Histochem Cytochem 57: 1003-1011.

14. Beutler E, Duparc S (2007) Glucose-6-phosphate dehydrogenase deficiency and antimalarial drug development. Am J Trop Med Hyg 77: 779-789.

15. Matsuoka H, Nguon C, Kanbe T, Jalloh A, Sato H, et al. (2005) Glucose-6-phosphate dehydrogenase (G6PD) mutations in Cambodia: G6PD Viangchan (871G>A) is the most common variant in the Cambodian population. J Hum Genet 50: 468-472.

16. Brewer GJ, Tarlov AR, Alving AS (1962) The methemoglobin reduction test for primaquine-type sensitivity of erythrocytes. A simplified procedure for detecting a specific hypersusceptibility to drug hemolysis. JAMA 180: 386-388.

17. Bernstein RE (1962) A rapid screening dye test for the detection of glucose-6-phosphate dehydrogenase deficiency in red cells. Nature 194: 192-193.

18. Cohen JM, Moonen B, Snow RW, Smith DL (2010) How absolute is zero? An evaluation of historical and current definitions of malaria elimination. Malar J 9: 213.

19. Hay SI, Guerra CA, Gething PW, Patil AP, Tatem AJ, et al. (2009) A world malaria map: *Plasmodium falciparum* endemicity in 2007. PLoS Med 6: e1000048.

20. Guerra CA, Howes RE, Patil AP, Gething PW, Van Boeckel TP, et al. (2010) The international limits and population at risk of *Plasmodium vivax* transmission in 2009. PLoS Negl Trop Dis 4: e774.

21. Tatem AJ, Smith DL, Gething PW, Kabaria CW, Snow RW, et al. (2010) Ranking of elimination feasibility between malaria-endemic countries. Lancet 376: 1579-1591.

22. The Global Health Group and the Malaria Atlas Project (2011) Atlas of Malaria-Eliminating Countries. San Francisco: The Global Health Group, Global Health Sciences, University of California, San Francisco.
