# Supplementary material for: G6PD Deficiency Prevalence and Estimates of Affected Populations in Malaria Endemic Countries: A Geostatistical Model-Based Map
Source: PLoS Med. 2012 Nov 13;9(11):e1001339. doi: 10.1371/journal.pmed.1001339 (PMC3496665; doi:10.1371/journal.pmed.1001339)
Supplement: Protocol S2 — Model based geostatistical framework for predicting G6PDd prevalence maps. (S2.1) Model requirements in relation to G6PD genetics. (S2.2) The model. (S2.3) Model implementation. (S2.4) Overview of mapping procedure. (S2.5) Uncertainty. (DOCX) [file pmed.1001339.s003.docx]

**Protocol S2. Model-based geostatistical framework for predicting G6PDd prevalence maps**

This protocol provides information about the geostatistical model used to map the prevalence of G6PDd. The general principles of model-based geostatistics have been previously described by Diggle and Ribeiro [[1](#_ENREF_1)]. The general framework of the model used here has been fully described by Piel et al. in application to HbS mapping [[2](#_ENREF_2)], though specific adaptations to that model were made to reflect the G6PD gene’s sex-linked inheritance mechanism.

**S2.1 Model requirements in relation to G6PD genetics**

The G6PD gene is carried on the X-chromosome, meaning that males inherit only a single copy of the gene (“hemizygotes”). In contrast, females have two copies so may carry two wild-type alleles or two deficient alleles (“homozygotes”), or a combination of one wild-type and one deficient (“heterozygotes”). The relative frequencies of each of these genotypes can be predicted if populations are assumed to be in Hardy-Weinberg equilibrium [[3](#_ENREF_3),[4](#_ENREF_4)], where $q$ represents the frequency of deficient alleles, making $(1-q)$ the allele frequency of normal G6PD expression. The overall population allele frequency corresponds to the frequency of male hemizygotes. As a consequence, the frequency of female homozygous deficiency expression corresponds to $q^{2}$, and $2q (1-q)$ in heterozygotes. However, for reasons discussed in Protocol S5, the heterozygous female genotype is often not expressed as phenotypically deficient, so only a proportion of genetically heterozygote females are identified in surveys and reported as deficient cases. There is therefore a genotype-phenotype disjunction between frequencies of observed deficient females and expected deficient females based upon Hardy-Weinberg derivations from frequencies of deficiency in males.

The model requirements were therefore to incorporate prevalence data relating to both males and females; and from this input dataset to generate continuous frequency estimates across malaria endemic countries (MECs; Protocol S1) with quantified uncertainty measures for rates of deficiency in (i) males, (ii) homozygous females, and (iii) all females – this final category being the combination of all homozygotes and the proportion of heterozygotes expected to be diagnosed as phenotypically deficient.

**S2.2 The model**

In this section, we describe our Bayesian spatial model for the G6PDd allele frequency surface $q(.)$. $q$ takes as its argument an arbitrary location on the Earth’s surface. The posterior $[q]$ induces a posterior $[qq]$ for the homozygous frequency surface $qq(.)$. We computed summaries of $[q(x)]$ and $[qq(x)]$, such as the mean $E$ and the variance $V$, at each location $x$, to produce the maps relating to G6PDd allele frequency. Predictions of expected genetic heterozygotes $[\{2q\left( 1-q \right)\}(x)]$ were adjusted to reflect the observation that many genetic heterozygotes are not phenotypically deficient. A deviance term, $h$, was determined by the model from the input data to represent the predicted deviation from the expected rates of genetic heterozygotes (see Likelihood). National and regional level prevalence of G6PDd in males and females was then computed from the model posterior distributions, as described in Protocol S4.

Prior

We model $q$ as a nonlinear transformation of a Gaussian random field [[5](#_ENREF_5),[6](#_ENREF_6)] $f(.)$, plus a random field $\varepsilon(.)$ that associates an independent normally distributed value with each location on the earth’s surface. Specifically,

$$q\left( x \right)=g\left( f\left( x \right)+\varepsilon\left( x \right) \right)$$

The link function $g$ maps the random variable $f\left( x \right)+\varepsilon\left( x \right)$, which can be any real number, to the interval (0,1), so $q(x)$ can be used as a probability or prevalence. We used a non-standard link function, which is described below.

The prior for $f$ is parameterized by the constant mean function $M(x)=m$, and the standard exponential covariance function $Cov\left( x,y \right)=\phi^{2}\exp\frac{d(x-y)}{\theta}$with amplitude parameter $\phi$ and range parameter $\theta$. The distance function $d$ gave the great-circle distance between $x$ and $y$, unless $x$ was on a different side of the Atlantic ocean to $y$, in which case it returned $\infty$. This modification prevented data in Africa from unduly influencing the east coast of south America. Suitable priors were assigned to the scalar parameters $m$*,* $\phi$ and $\theta$:

$$p\left( m \right)\propto1$$

$$\phi\sim Exponential\left( .1 \right)$$

$$\theta\sim Exponential\left( .1 \right)|(\theta<0.5)$$

$$f\sim GP(M, Cov)$$

GP indicates a Gaussian process. The units of $x$*,* $y$ and $\theta$ are earth radii, and $m$ and $\phi$ are unitless. The unstructured component $\varepsilon(x)$ is modelled as normally distributed with unknown variance $V$:

$$V\sim Exponential\left( .1 \right)$$

$$\varepsilon\left( x \right) \begin{matrix} iid \\ \sim\end{matrix} Normal(0,V)$$

The distribution parameters for $h$, the deviance term from expected Hardy-Weinberg deficiency rates in heterozygous females were defined by $\alpha$ and $\beta$, where:

$$h \sim Beta(\alpha,\beta)$$

$$\alpha\sim Exponential\left( .01 \right)$$

$$\beta\sim Exponential(.01)$$

Likelihood

Separate likelihoods were defined for males and females, in accordance with their different inheritance mechanism of the G6PD gene.

*In males*: The frequency of G6PDd in males corresponds to the population allele frequency of deficiency, $q$. If $n_{i}$ male individuals are sampled at the $i$^th^ observation location $o_{i}$, the probability distribution for the number $k_{i}$ of copies of the G6PDd allele that will be found is binomial, with probability $q(o_{i})$:

$$k_{i}\sim Binomial(n_{i}, q\left( o_{i} \right))$$

*In females*: Under the assumptions of Hardy-Weinberg equilibrium [[3](#_ENREF_3),[4](#_ENREF_4)], if $n_{i}$ female individuals are sampled at the $i$^th^ observation location $o_{i}$ (for a total of $2n_{i}$ chromosomes), the probability distribution for the number $k_{i}$ of G6PDd females is binomial, with probability $q(o_{i})$:

$$k_{i}\sim Binomial(2n_{i}, h_{i}(2q\left( o_{i} \right)\left( 1-q\left( o_{i} \right) \right))+{q\left( o_{i} \right)}^{2})$$

Flexible link function and empirical Bayesian analysis

The link function $g$ for binomial data is usually taken to be the inverse logit function:

$$g\left( x \right)=logit^{-1}\left( x \right)=\frac{\exp x}{1+\exp x}$$

Piel et al. [[7](#_ENREF_7)] employed this model. Applying the change of variables formula, the induced prior for $q(x)$ is:

$$p\left( q\left( x \right) \right)=\frac{1}{q\left( x \right)\left( 1-q\left( x \right) \right)}Normal\left( logit\left( q\left( x \right) \right);m, \phi^{2}+ V \right)$$

Note that this is essentially a two-parameter family of probability distributions, since $\phi$ and $V$ appear only in the sum.

Under this model, in areas where data points are highly clustered the best fitting values of $m$ and $\phi^{2}+V$ result in long right-hand tails for the predictive distribution of prevalence in the next observation at $x$, thus skewing the summary statistics to give implausibly high predicted median values. To overcome this problem, we used an alternative flexible link function:

$$j\left( x \right)= \sum_{i=0}^{3} Ȼ_{i}x^{i}$$

$$g=logit^{-1} ^{\circ} j$$

We were unable to infer $Ȼ_{i}$ jointly with the other model parameters in a fully Bayesian manner due to poor MCMC mixing, so we adopted an empirical fitting approach inspired by data pre-processing steps employed in classical geostatistics. This improved the fitting of the model to the data and is described in more detail below.

Empirical Bayesian approach to fitting the polynomial coefficients

For each observation of $n_{i}$ males tested and $k_{i}$ deficient males identified, we first obtained the posterior expectation of the gene pool-wide prevalence of G6PDd with uniform prior density on [0,1]:

$$\hat{p}_{i}=\frac{k_{i}+1}{n_{i}+2}$$

We discarded values for which $n_{i}$ was below 25. Then, we inferred the parameters $\tilde{m}$ and $\tilde{V}$ of the non-spatial Bayesian model:

$$p\left( \hat{p} \right)=\prod_{i} \frac{1}{\hat{p_{i}}\left( 1-\hat{p_{i}} \right)}Normal\left( logit\left( \hat{p_{i}} \right);\tilde{m}, \tilde{V} \right)$$

We then plotted the posterior predictive cumulative distribution function (CDF) of $logit(\hat{p})$ against its empirical CDF, and fitted the coefficients *Ȼ* of the cubic polynomial function $j$ to the points using least squares, subject to the constraint that $j$ must be invertible (or, equivalently, monotone).

The polynomial coefficients for such a function are specific to the dataset. The set of coefficients used, corresponding to an invertible function and fitting the empirical CDF was:

$$y= 0.15200304x^{3}+ 1.12465599x^{2}+ 0.03658898x+ 0.00342442$$

In the Bayesian analysis of the full spatial model, the fitted values of Ȼ were taken as known and fixed. Although this empirical procedure is admittedly informal, the resulting non-standard link function did substantially improve the fit of the model to the data.

Prior predictive constraint

The G6PDd database was largely composed of opportunistic community surveys. As such, data points were unevenly distributed across the malaria endemic region (Figure 2.1A), and the model had to be adapted to this. Inclusion of the highly clustered datasets, particularly in the Philippines, did not appear to alter predictions across the rest of the map. In contrast, high and low latitude areas which were sparse in data (such as northern China and Argentina) were very hard for the model to predict, generating implausibly high population estimates of G6PDd prevalence. Based on our knowledge of the distribution of G6PDd from existing maps [[8-10](#_ENREF_8)], it seemed reasonable to assume that G6PDd was at low prevalence in these areas, supporting the principle that most surveys will have been conducted where G6PDd was expected to be found. To introduce this into the model, we decided to constrain $\phi$, $m$ and $V$ in such a way that the prior predictive distribution of $q(x)$, before the data are incorporated, puts probability mass of 1×10^-4^ or less on values in excess of 0.0001. In other words, we constrained 99.99% of the prior predictive probability mass to be between allele frequencies of 0% and 0.01%.This constraint arguably induces a lack of fit by forcing $f(x)$ to depart from its prior mean by many standard deviations in areas where G6PDd allele frequency is known to be high; but it does remedy the implausibly high predictive values in the data-sparse edges of the map, and does not seem to adversely affect the fit in areas of non-zero allele frequency. A detailed explanation of this process is given by Piel et al. [[2](#_ENREF_2)].

**S2.3 Model implementation**

As previously described [[11](#_ENREF_11)], implementation of the modelling and mapping procedure was divided into two computational tasks: (i) the Bayesian inference stage was implemented using the Markov chain Monte Carlo (MCMC) algorithm [[12](#_ENREF_12),[13](#_ENREF_13)] and was used to generate samples from the posterior predictive distribution (PPD) of the parameter set and the spatial random fields at the data locations; and (ii) a prediction stage in which samples were generated from the PPD of G6PDd frequencies at each pixel on the 5×5 km grid, as described below in Protocol 2.5.

The scalar parameters $\phi$, $m$, $\theta$ and $V$ were updated jointly using Haario, Saksman and Tamminen’s adaptive Metropolis algorithm [[14](#_ENREF_14)], as implemented by PyMC’s AdaptiveMetropolis step method. Each value of *ε(o_i_)* at observation location $o_{i}$ was updated separately using the standard one-at-a-time Metropolis algorithm. The distribution of the Gaussian random field at the observation locations, $\{f\left( o_{i} \right)\}$, is conjugate to the distribution of $\{\varepsilon\left( o_{i} \right)+f\left( o_{i} \right)\}$, so we updated $\left\{ f\left( o_{i} \right) \right\}$ by sampling from its full conditional distribution. MCMC output parameter values are summarised in Table S2.1.

Convergence of the MCMC tracefile was judged by visual inspection; one million MCMC iterations were run, with 10% recorded in the output tracefile, the first 50,000 iterations of which were excluded from the mapping stages. During the mapping process, the posterior distributions were thinned by 100, resulting in 500 mapping iterations. MCMC dynamic traces are available on request.

The model code was written in Python programming language (<http://www.python.org>), and is freely available from the MAP’s code repository (<https://github.com/malaria-atlas-project> and <https://github.com/RosalindH/g6pd>). The MCMC algorithm was used from the open-source Bayesian analysis package PyMC [[15](#_ENREF_15)] (<http://code.google.com/p/pymc>).

**S2.4 Overview of mapping procedure**

The output algorithm from the MCMC was used to generate PPDs for each model output at all pixels in the MEC 5×5 km grids. The PPDs can be used to determine the most probable prevalence estimate at each pixel [[5](#_ENREF_5)], these were summarised for each pixel as median values, together with the PPD’s interquartile range (IQR). The median was determined to be more representative than the mean value, due to the PPD’s right-hand skew, inflating the mean values of the predictions. Maps were generated using Python and Fortran code, available from the MAP’s code repository (<http://github.com/malaria-atlas-project/generic-mbg>) and subsequently displayed in GIS software (ArcMap 10.0, ESRI Inc., Redlands, CA, USA).

**S2.5 Uncertainty**

Uncertainty metrics.

The MCMC and derived map predictions are directly informed by the evidence-base of surveys. However, there is usually some level of disparity within this database (i.e. in clustered areas) and between the database and the map predictions. The relative influence of each point’s influence on the model predictions is moderated according to the data points’ sample size (through a binomial model previously described [[11](#_ENREF_11)]), their spatial distribution and the level of heterogeneity in the frequencies identified between neighbouring data points. These three factors influence the model’s ability to predict frequencies, which are then reflected in the spread of the PPD. The precision of the PPD is an indicator of the representativeness of the summary statistic used (in this case median values) [[5](#_ENREF_5)] and is quantified here by the interquartile range (IQR) of the PPD, thus corresponding to a 50% probability class. However, because the values of the IQR are affected by the underlying G6PDd prevalence levels, with IQR generally increasing with prediction values. The IQR was therefore also standardised against the median map to produce an uncertainty index less affected by the underlying prevalence levels and more illustrative of relative model performance driven by data densities in different locations ($\frac{IQR}{Median}$). Maps of both uncertainty metrics are presented for comparison (Figure S2.1).

Uncertainty maps.

Greatest absolute variation in the predictions (Figure S2.1B) was found from areas of relatively high predicted frequencies and low input data availability (Figure S2.1A), specifically southern Pakistan, the central Sahel region across Chad and Sudan, and southern central Africa (Democratic Republic of Congo (DRC), Zambia, Malawi, southern Tanzania and Mozambique) and Madagascar. Model predictions for these areas should be interpreted with caution; additional surveys from these areas would enable more robust predictions.

Adjusting the IQR values to the predicted median frequency values gives a representation of the relative variability in the PPD (Figure S2.2B). The greatest relative prediction uncertainty is in peripheral transition regions, such as the Horn of Africa and southern Africa – where prevalence drops relative to surrounding areas. The high IQR region across DRC and neighbouring areas is not so pronounced in the proportional map. Greater uncertainty is shown across the Americas, previously masked in the raw IQR map (Figure S2.2A). The large proportion of northern China which is uninformed by data has very high relative uncertainty, reflecting an uncertainty in the model about where exactly to bring down the prevalence predictions to the very low levels predicted across the north of the country. An equivalent increase in uncertainty and associated data absence is seen on Borneo.

These two uncertainty metrics complement each other, allowing interpretation of confidence in the map predictions and can be used to identify areas where additional data would be most informative towards improving our understanding of G6PDd prevalence.

| **Parameter** | **Symbol** | **Mean** | **Median** | **St. Dev.** | **IQR** | **95% BCI** |
| --- | --- | --- | --- | --- | --- | --- |
| Nugget variance | $V$ | 0.239 | 0.226 | 0.080 | 0.037 | 0.190 |
| Amplitude (or partial sill) | $\emptyset$ | 3.422 | 3.426 | 0.202 | 0.213 | 0.919 |
| Scale (or range) | $\theta$ | 0.498 | 0.499 | 0.003 | 0.002 | 0.007 |

**Table S2.1. MCMC output parameter values**. Summary values of the model parameters, as fully described in Protocol S2. Summary statistics of the MCMC output include mean, median, standard deviation (St. Dev.), interquartile range (IQR) and the 95% Bayesian credible interval (95% BCI). Scale is measured in units of Earth radii; other parameters are unitless.


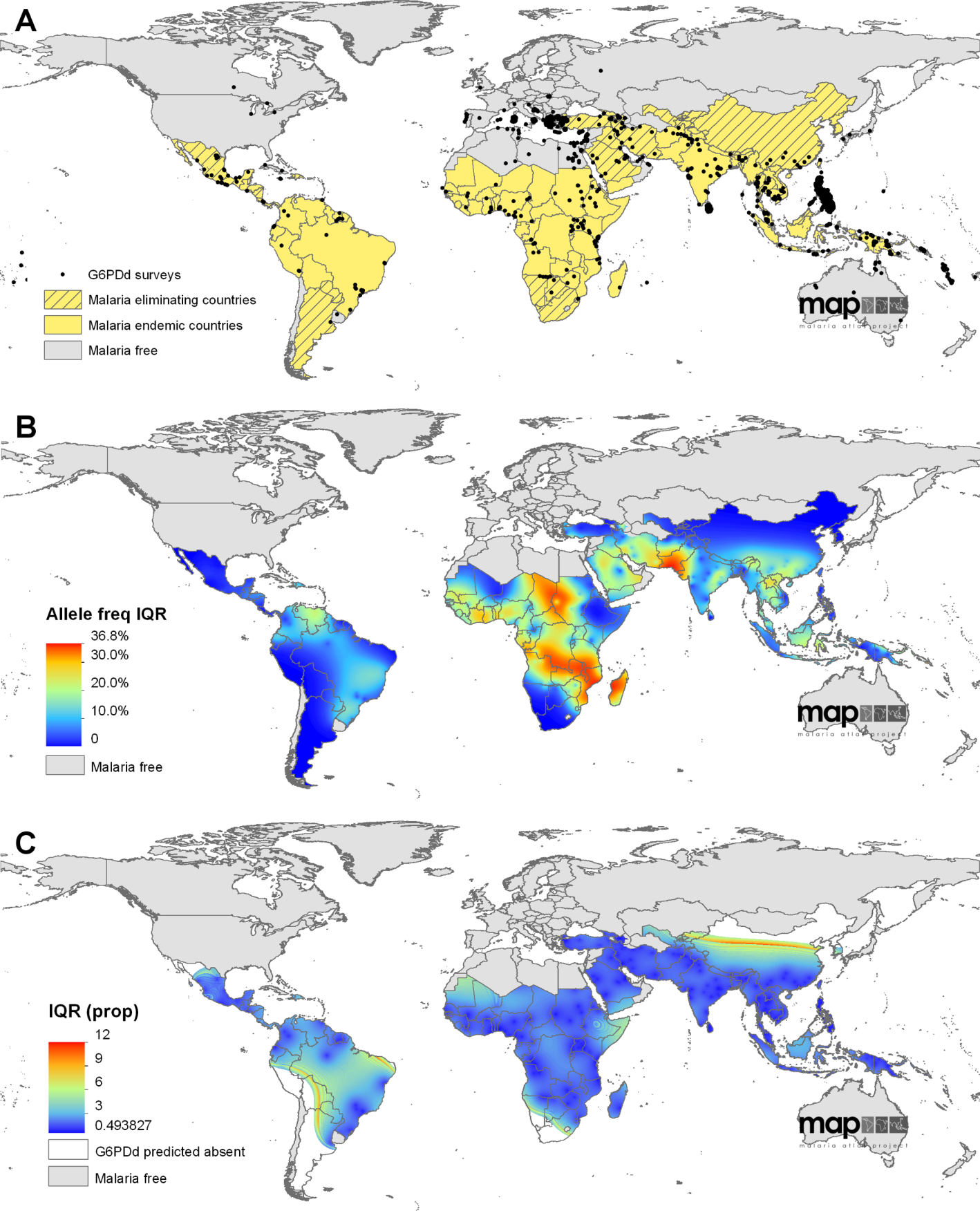
 **Figure S2.1. Prediction uncertainty metrics.** Panel A shows the input surveys database. Panel B is the interquartile-range (IQR) of the PPD of male G6PDd prevalence, representing absolute values of map uncertainty. The map in Panel C was derived directly from the IQR map, adjusted to the median values.

**References**

1. Diggle PJ, Ribeiro PJ, Jr. (2007) Model-based Geostatistics: Springer.

2. Piel FB, Patil AP, Howes RE, Nyangiri OA, Gething PW, et al. Global estimates of sickle haemoglobin in newborns. The Lancet: In press.

3. Hardy GH (1908) Mendelian Proportions in a Mixed Population. Science 28: 49-50.

4. Weinberg W (1908) Über den nachweis der vererbung beim menschen. Jahreshefte des Vereins für vaterländische Naturkunde in Württemberg 64: 368-382.

5. Patil AP, Gething PW, Piel FB, Hay SI (2011) Bayesian geostatistics in health cartography: the perspective of malaria. Trends Parasitol 27: 246-253.

6. Banerjee S, Carlin BP, Gelfand AE (2004) Hierachical modeling and analysis for spatial data. Monographs on Statistics and Applied Probability 101. Boca Randon, Florida, U.S.A.: Chapman & Hall / CRC Press LLC.

7. Piel FB, Patil AP, Howes RE, Nyangiri OA, Gething PW, et al. (2010) Global distribution of the sickle cell gene and geographical confirmation of the malaria hypothesis. Nat Commun 1: 104.

8. Cavalli-Sforza LL, Menozzi P, Piazza A (1994) The History and Geography of Human Genes. Princeton, New Jersey: Princeton University Press. 1088 p.

9. WHO Working Group (1989) Glucose-6-phosphate dehydrogenase deficiency. Bull World Health Organ 67: 601-611.

10. Nkhoma ET, Poole C, Vannappagari V, Hall SA, Beutler E (2009) The global prevalence of glucose-6-phosphate dehydrogenase deficiency: a systematic review and meta-analysis. Blood Cells Mol Dis 42: 267-278.

11. Hay SI, Guerra CA, Gething PW, Patil AP, Tatem AJ, et al. (2009) A world malaria map: *Plasmodium falciparum* endemicity in 2007. PLoS Med 6: e1000048.

12. Gilks WR, Richardson S, Spiegelhalter D (1995) Markov chain Monte Carlo in Practice: Chapmand & Hall/CRC.

13. Gelman A, Carlin JB, Stern HS (2003) Bayesian Data Analysis. Texts in Statistical Science. Boca Raton, Florida, U.S.A.: Chapman & Hall / CRC Press LLC.

14. Haario H, Saksman E, Tamminen J (2001) An Adaptive Metropolis Algorithm. Bernoulli 7: 223-242.

15. Patil A, Huard D, Fonnesbeck CJ (2010) PyMC: Bayesian Stochastic Modelling in Python. J Stat Softw 35: 1-81.
