# Supplementary material for: G6PD Deficiency Prevalence and Estimates of Affected Populations in Malaria Endemic Countries: A Geostatistical Model-Based Map
Source: PLoS Med. 2012 Nov 13;9(11):e1001339. doi: 10.1371/journal.pmed.1001339 (PMC3496665; doi:10.1371/journal.pmed.1001339)
Supplement: Protocol S3 — Model validation procedures and results. (S3.1) Creation of the validation datasets. (S3.2) Model validation methodology. (S3.3) Validation results. (DOCX) [file pmed.1001339.s004.docx]

**Protocol S3. Model validation procedures and results**

To assess the model’s predictive ability, a validation run was used to quantify the disparity between the model’s predictions and a hold-out subset of the data [[1](#_ENREF_1),[2](#_ENREF_2),[3](#_ENREF_3),[4](#_ENREF_4)]. The disparities were quantified to summarise general trends in the model’s predictions.

**S3.1 Creation of the validation datasets**

To ensure that the validation metrics were representative of the overall model performance, it was necessary to ensure that the validation dataset was also representative of the full predicted surface. However, the dataset included some strong spatial clustering of surveys where small geographic areas had been heavily surveyed (e.g. the Philippines, Sri Lanka and the Kenyan coast). To ensure that data points from these areas (which are inherently more likely to be easier to predict, even with expected heterogeneity in allele frequency) were not over-represented in the validation procedure, a spatial-declustering sampling procedure was applied to the dataset. The details of this procedure have been previously described by Hay et al. [[5](#_ENREF_5)]. This increased the probability that isolated samples will be included in the hold-out dataset, thus corresponding to areas which will be harder for the model to predict. A spatially-declustered 5% subset of the data (n=86) was therefore withheld from the validation MCMC, which was run with the remaining 95% (n=1,648) of the dataset (Figure S3.1A). It was not possible to use a hold-out sample larger than 5% with the declustering algorithm as the remaining dataset would have been too sparse to allow plausible mapping, thus making the validation process unrepresentative.

A second validation procedure was run with a randomly selected 10% subset of the data (n=173). Selecting the dataset at random meant that the geographical distribution of the hold-out dataset was more likely to be representative of the distribution of the overall dataset (Figure S3.1B).

**S3.2 Model validation methodology**

The Bayesian geostatistical G6PDd model was implemented in full with the thinned datasets (n=1,648 and n=1,561). This generated full PPDs, predictions from which could be compared with those in the hold-out datasets (from n=86 and n=173 locations, respectively), with differences summarised with simple statistical measures. Mean error was used to assess the model’s overall bias, and the mean absolute error quantified the overall prediction accuracy as the average magnitude of errors in the predictions. These are fully described by Hay et al. [[5](#_ENREF_5)]. A scatter plot was also generated as a visualisation of the correspondence between the predicted and actual values.

As well as considering the model’s ability to predict point estimates, it was also important to assess the extent to which the model PPDs provided a suitable measure of uncertainty. A previously developed process [[3](#_ENREF_3),[6](#_ENREF_6),[7](#_ENREF_7)] was used to test how well the validation sets of n=86 and n=173 PPDs captured the true uncertainty in the model output. Credible intervals (CIs) define a range of candidate values associated with a specified predicted probability of occurrence. Working through 100 progressively narrower CIs, from the 99% CI to the 1% CI, each was tested by computing the actual proportion of the hold-out prevalence observations that fell within the predicted CI. Plotting these actual proportions against each predicted CI level allowed the overall fidelity of the PPDs at the hold-out data locations to be assessed.

The bespoke dataset spatial declustering code and validation code are freely available from the MAP online code repository (<https://github.com/malaria-atlas-project/generic-mbg>).

**S3.3 Validation results**

The declustered validation data subset was a more stringent validation test than the randomly selected dataset, as the model’s predictive ability was being preferentially tested in areas where fewer data were available to inform the predictions (Figure S3.2 and Table S3.1). The mean error values reveal a slight tendency to overestimate G6PDd frequency by 1.45% and 0.17% in the declustered and randomly selected validation runs respectively. So although the model has relatively low overall prediction bias, the magnitude of the variance between the predicted and observed prevalence can be more substantial, as indicated by mean absolute errors of 4.07 and 3.48%. These larger discrepancies may be due to the absence of nearby data points and to local heterogeneity in G6PDd values.

The probability-probability plots comparing predicted quantiles with observed coverage fractions (Figures 3.2C-D) show the fraction of the observations that were actually contained within each predicted CI. These plots show a good degree of fidelity in the predicted quantiles, indicating that the IQR measures used to represent uncertainty are a good representation of the model predictions.

| **Validation metric** | **Declustered 5%** | **Random 10%** |
| --- | --- | --- |
| Hold-out dataset | n=86 | n=173 |
| Thinned dataset | n=1,648 | n=1,561 |
| Mean error | 1.45% | 0.17% |
| Mean absolute error | 4.07% | 3.48% |

**Table S3.1. Summary of the validation statistics**. Values are given in % frequency of G6PDd. Mean error statistics summarise the model’s overall predictive bias (over/under-estimating); mean absolute errors indicate the magnitude of those errors.


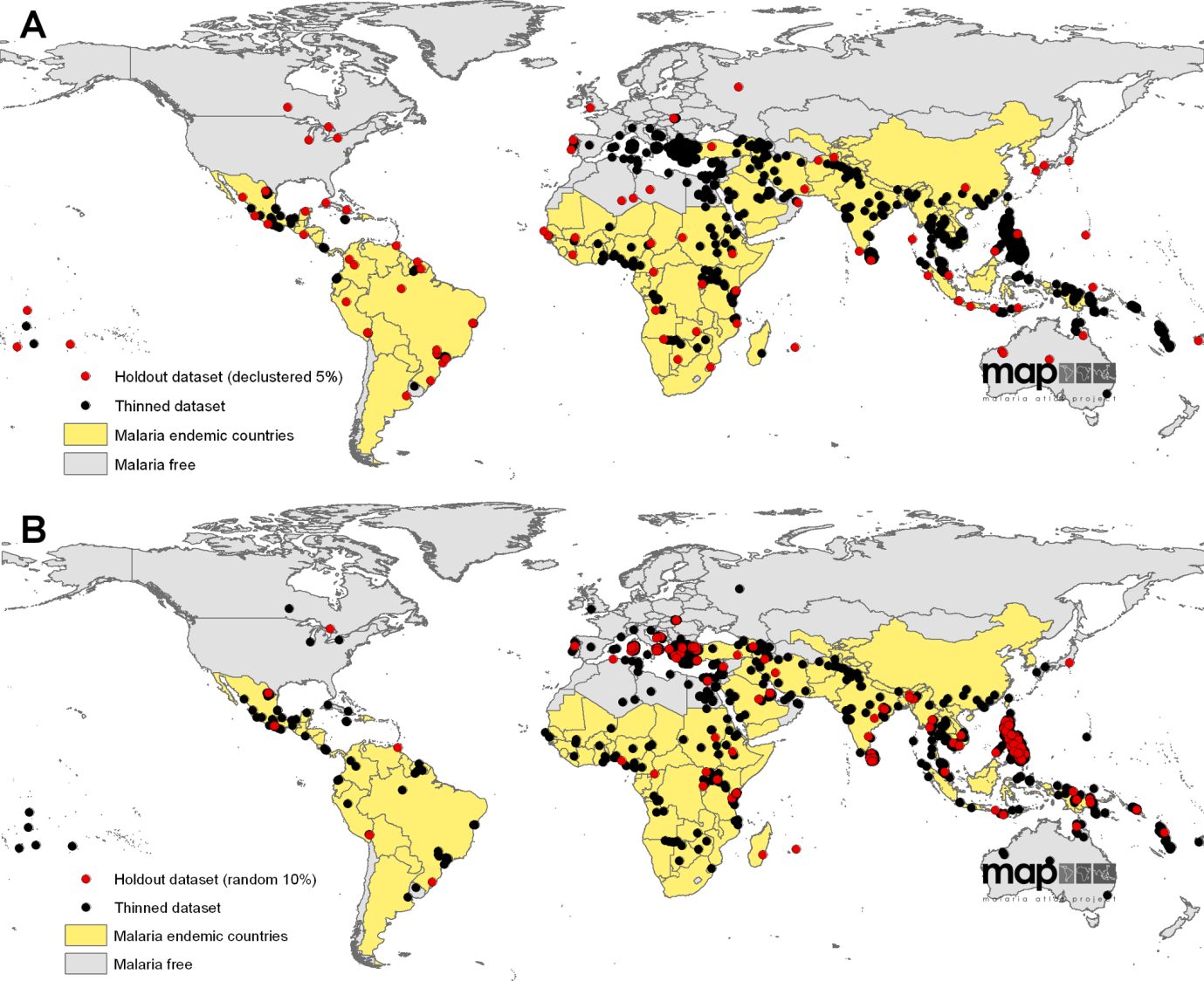


**Figure S3.1. Distribution of the hold-out data subsets used for model validation.** Red points are those held-out from the model run and used for validation of the model’s predictions based on the remaining thinned dataset (black data points). Panel A shows the spatially declustered 5% hold-out dataset; Panel B shows the randomly selected 10% hold-out dataset.


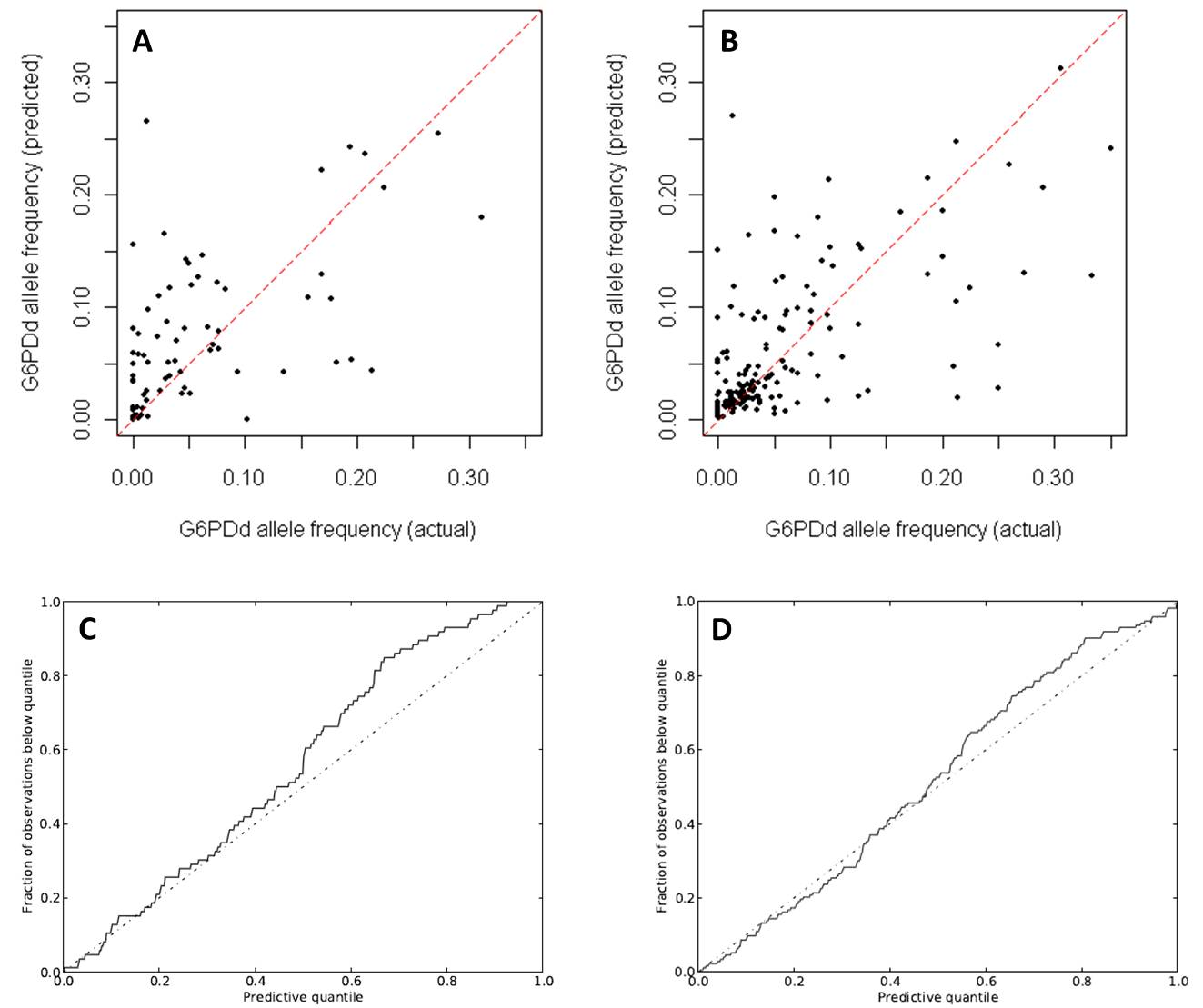


**Figure S3.2. Model validation plots for both validation processes.** Panels A and C correspond to the declustered 5% hold-out (n=86), B and D are the randomly selected 10% hold-out (n=173). Panels A and B are scatter plots of actual versus predicted point-values of G6PDd. Panels C and D show the probability-probability plots comparing predicted credible intervals with the actual percentage of true values lying in those intervals. The 1:1 line is also shown for reference.

**References**

1. Gething PW, Elyazar IR, Moyes CL, Smith DL, Battle KE, et al. A long neglected world malaria map: *Plasmodium vivax* endemicity in 2010. Public Library of Science NTD: In press.

2. Gething PW, Patil AP, Smith DL, Guerra CA, Elyazar IR, et al. (2011) A new world malaria map: *Plasmodium falciparum* endemicity in 2010. Malar J 10: 378.

3. Piel FB, Patil AP, Howes RE, Nyangiri OA, Gething PW, et al. Global estimates of sickle haemoglobin in newborns. The Lancet: In press.

4. Howes RE, Patil AP, Piel FB, Nyangiri OA, Kabaria CW, et al. (2011) The global distribution of the Duffy blood group. Nat Commun 2: 266.

5. Hay SI, Guerra CA, Gething PW, Patil AP, Tatem AJ, et al. (2009) A world malaria map: *Plasmodium falciparum* endemicity in 2007. PLoS Med 6: e1000048.

6. Gething PW, Noor AM, Gikandi PW, Hay SI, Nixon MS, et al. (2008) Developing geostatistical space-time models to predict outpatient treatment burdens from incomplete national data. Geogr Anal 40: 167-188.

7. Moyeed RA, Papritz A (2002) An empirical comparison of kriging methods for nonlinear spatial point prediction. Mathematical Geology 34: 365-386.
