# Supplementary material for: G6PD Deficiency Prevalence and Estimates of Affected Populations in Malaria Endemic Countries: A Geostatistical Model-Based Map
Source: PLoS Med. 2012 Nov 13;9(11):e1001339. doi: 10.1371/journal.pmed.1001339 (PMC3496665; doi:10.1371/journal.pmed.1001339)
Supplement: Protocol S4 — Demographic database and population estimate procedures. (S4.1) GRUMP-beta human population surface. (S4.2) Areal prediction procedures. (DOCX) [file pmed.1001339.s005.docx]

**Protocol S4. Demographic database and population estimate procedures**

Both G6PDd prevalence and population density are heterogeneous in their distributions. To reflect both these sources of spatial variation in our estimates of G6PDd populations, we used high resolution population density grids to weight the G6PDd predictions and generate a single representative predictive posterior distribution (PPD) and summary prevalence estimates for each national and regional area of interest.

**S4.1 GRUMP-beta human population surface**

We used the Global Rural Urban Mapping Project (GRUMP) *beta* grids of population counts rescaled to a 5×5 km spatial resolution (from the original 1×1 km resolution), adjusted to UN national population total estimates for 2010 [[1](#_ENREF_1),[2](#_ENREF_2),[3](#_ENREF_3)]. National-level sex-ratio population data were also taken from the UN World Population Prospects [[4](#_ENREF_4)].

**S4.2 Areal prediction procedures**

To account for model uncertainty, the full MCMC tracefile, and not simply the mapped median values, was used to estimate aggregated population numbers affected by G6PDd [[5](#_ENREF_5)]. The areal prediction model is fully described by Piel et al. [[6](#_ENREF_6)], but a brief conceptual overview is given here. The model aimed to generate a summary description of G6PDd prevalence across each country and regional MEC aggregation of interest. The model sampled the MCMC tracefile repeatedly from the population-weighted selected sites across the areal region of interest (30,000 and 1,000 spatial points from the MEC regional and each national area, respectively). The model weighted the G6PDd estimate according to population density (at a 5×5 km grid resolution), thus generating a single summary PPD for the overall region. Each summary prevalence estimate was then related to the total population estimate for 2010 from that region, accounting for the national sex-ratio [[4](#_ENREF_4)]. The PPDs allowed us to quantify the predictions’ uncertainty as done with the summary maps.

As with the summary allele frequency map, summaries of the areal-prediction PPDs are given as median and IQR values (Supplementary Table S1 and S2). However, it is important to note that the sum of median values is not equivalent to the median of sums [[5](#_ENREF_5)]. As a result, differences can be observed between the regional and the sums of national G6PDd population median estimates in the region.

Median values were also used to derive the homozygous frequencies directly from the areal allele frequency estimates (as opposed to from the full MCMC, as in for the male & all female estimates) as these corresponded to the estimates which would have been generated if we had used the full pixel-level method of estimation of homozygosity rates. Additionally, to generate Monte Carlo standard error estimates of the areal prediction model, these were repeated ten times for each national spatial aggregate and five times for the regional aggregates. The variation around the mean of each set of repeated summary statistics (mean, median, 25% and 75% quartiles) is given as standard errors (SE) in Supplementary Table S2.

The code used to implement this analysis is freely available at https://github.com/malaria-atlas-project/.

**References**

1. Balk DL, Deichmann U, Yetman G, Pozzi F, Hay SI, et al. (2006) Determining global population distribution: methods, applications and data. Adv Parasitol 62: 119-156.

2. United Nations Department of Economics and Social Affairs (2008) World Population Prospects: the 2008 revision population database. New York: United Nationals Population Division (U.N.D.P.).

3. Hay SI, Guerra CA, Gething PW, Patil AP, Tatem AJ, et al. (2009) A world malaria map: *Plasmodium falciparum* endemicity in 2007. PLoS Med 6: e1000048.

4. United Nations Department of Economics and Social Affairs (2011) World Population Prospects, the 2010 Revision. New York: United Nationals Population Division.

5. Patil AP, Gething PW, Piel FB, Hay SI (2011) Bayesian geostatistics in health cartography: the perspective of malaria. Trends Parasitol 27: 246-253.

6. Piel FB, Patil AP, Howes RE, Nyangiri OA, Gething PW, et al. Global estimates of sickle haemoglobin in newborns. The Lancet: In press.
