# Supplementary material for: G6PD Deficiency Prevalence and Estimates of Affected Populations in Malaria Endemic Countries: A Geostatistical Model-Based Map
Source: PLoS Med. 2012 Nov 13;9(11):e1001339. doi: 10.1371/journal.pmed.1001339 (PMC3496665; doi:10.1371/journal.pmed.1001339)
Supplement: Protocol S5 — Mapping the prevalence of G6PDd in females. (S5.1) Overview of G6PDd in females. (S5.2) Heterozygous G6PDd expression and diagnosis. (S5.3) Overview of female data in the G6PD database. (S5.4) Modelling phenotypic G6PDd prevalence in females. (S5.5) Maps of G6PDd in females and population estimates. (S5.6) Improving the map of G6PDd in females. (DOCX) [file pmed.1001339.s006.docx]

**Protocol S5. Predicting the prevalence of G6PDd in females**

**S5.1 Overview of G6PDd in females**

The implications of the G6PD gene’s position on the X-chromosome on its genetics and inheritance mechanisms are particularly pertinent to estimates of the prevalence of G6PDd in heterozygous females. Reasons for these complexities have been previously reviewed [[1-3](#_ENREF_1)], and are briefly discussed here.

**S5.2 Heterozygous G6PDd expression and diagnosis**

In a population at Hardy-Weinberg equilibrium where G6PDd allele frequency is <0.5, prevalence of heterozygous females will be higher than that of affected males [[4](#_ENREF_4)] (frequencies >0.5 were not identified during our data searches apart from one instance where individuals tested was n<10). However, not all heterozygous females express a phenotypically significant deficiency; the difficulty of diagnosing deficiency in females, and extrapolating these figures to national levels has been previously discussed [[5](#_ENREF_5)].

The gene’s X-chromosome genetics means that heterozygotes have a mosaic-effect of G6PD expression, with two populations of red blood cells – those expressing the wild-type G6PD gene, and the rest bearing the deficiency [[6](#_ENREF_6)]. This is due to the phenomenon of Lyonization whereby only one X-chromosome is actively expressed in each cell [[7](#_ENREF_7)]. Lyonization is a random process and the resulting proportions of normal and deficient cells may deviate significantly away from the expected 50:50 ratio [[3](#_ENREF_3)], leading some heterozygotes to have virtually normal expression, and others with expression levels comparable to female homozygotes (i.e. entirely deficient). Enzyme expression is therefore mixed, and inherently hard to predict based on genotype. Depending upon the ratio of the two cell populations, only a minority of heterozygotes will be clinically deficient and thus identified by the standard enzyme deficiency tests which usually detect deficiencies below about 20% of normal wild-type expression levels. Therefore, while deficiency may be readily diagnosed in homozygous females, heterozygote expression is variable and harder to determine.

As well as mosaic-expression of G6PD, the variability of expression among heterozygotes is partly attributable to the diversity of G6PD mutations, as many as 160 genetic variants have been described [[1](#_ENREF_1)]. These cause a spectrum of deficiency levels, from <1% normal activity levels to having no discernible effect on enzyme activity [[1](#_ENREF_1)]. As a result, the residual enzyme activity levels in females will reflect this range; the proportion of enzymatically deficient heterozygotes will therefore be spatially variable, determined by the local molecular variants [[8](#_ENREF_8)].

Illustrating these issues, the diagnostic sensitivity of the WHO-recommended fluorescent spot test was found to be only 32% (with 99% specificity) for heterozygote detection [[2](#_ENREF_2)]. Although a few biochemical tests have been described which are better suited to detecting heterozygosity, such as G6PD/6PDG and G6PD/PK ratio analysis, and the cytochemical G6PD staining assay [[2](#_ENREF_2),[9](#_ENREF_9)], these are impractical for large-scale, field-based population surveys, and rarely used.

Whilst acknowledging that the common phenotypic tests have low sensitivity in detecting heterozygotes, and therefore that a large proportion of genotypically deficient individuals will escape identification, the aim of the population estimates presented in this paper is to represent the prevalence of individuals with clinically significant deficiency, as determined by the common phenotypic diagnostic tests.

**S5.3 Overview of female data in the G6PD database**

Of the 1,734 locations reporting rates of G6PDd in the database, 1,067 (62%) reported rates in females, with only 14 giving rates in females exclusively. The map in Figure 2A in the main paper shows their geographic distribution, and that of the male-only data. The prevalence values reported for females ranged from 0% (in 301 of the 1,067 female surveys) to 51% (from a survey of 100 females in the Solomon Islands [[10](#_ENREF_10)]). Prevalence estimates in females are compared to those in males in Figure S5.1. As would be expected, there is a general correlation between prevalence in males and females in the same population (R^2^ = 74.9%). Female estimates tend to be lower than those in corresponding male samples, though these patterns are heterogeneous.


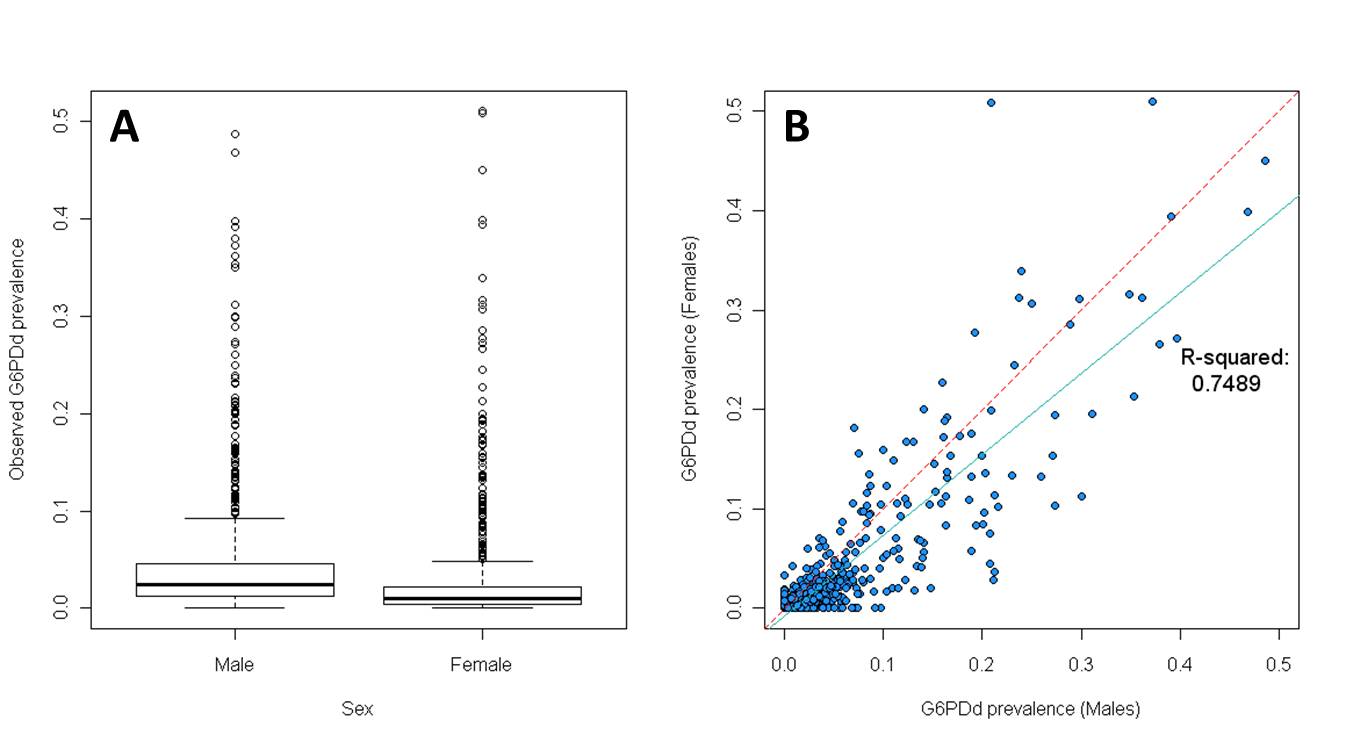
**Figure S5.1. Observed G6PDd prevalence in males and females.** Data points are from database raw data. Only surveys sampling both sexes and with sample sizes ≥50 are included in these plots (n = 725). Panel A summarises the overall deficiency prevalence estimates; Panel B plots the observed co-occurring male and female prevalence estimates. Panel B also shows the 1:1 line (dashed red line) and the line of best fit (continuous blue line).

For reasons previously discussed (Protocol S2), estimates of the prevalence of homozygosity can be calculated using simple rules of inheritance ($q^{2}$). It is therefore possible to subtract this predicted number of homozygous females, who are highly likely to be phenotypically deficient, from the overall observed number of deficient females, and compare prevalence of observed and expected genetically heterozygous females ($2pq$) (Figure S5.2). This provides an estimate of the observed deviance in heterozygous expression away from expected Hardy-Weinberg proportions. This deviance corresponds to $h$ – an estimate of the proportion of heterozygous females who will not be diagnosed as phenotypically deficient (Protocol S2).


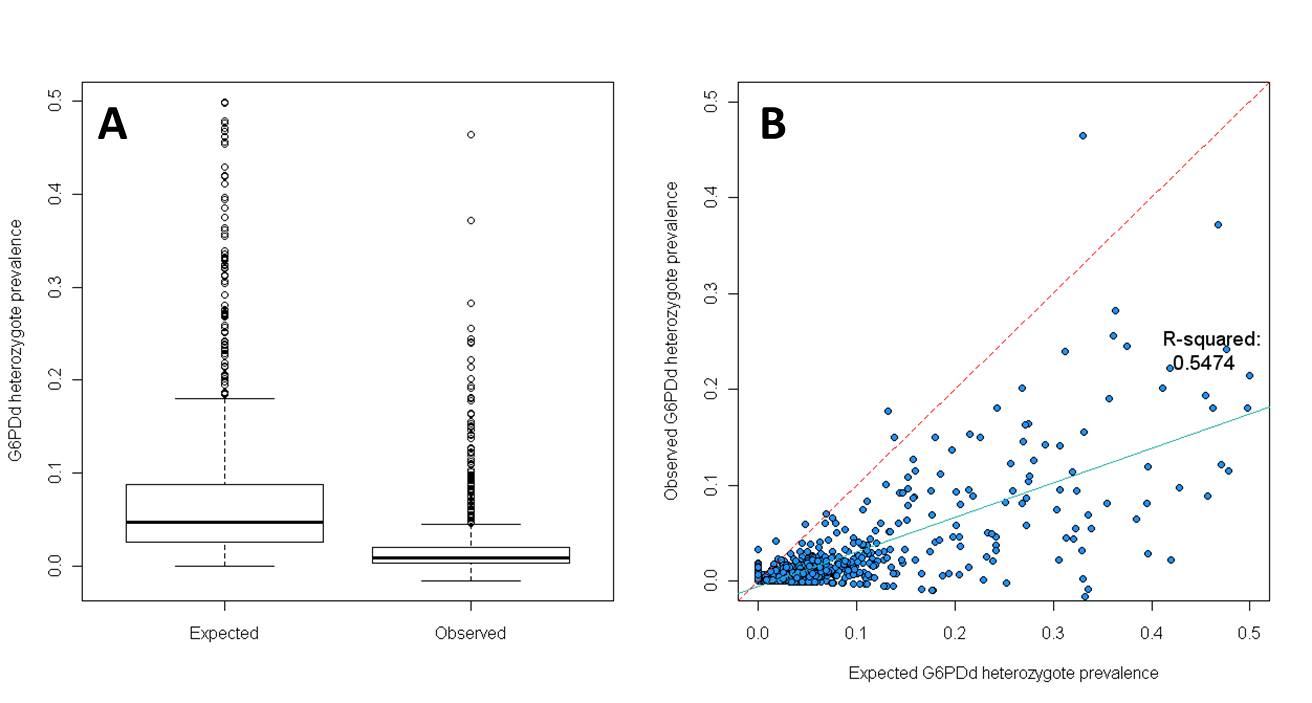
 **Figure S5.2. Comparison of expected and observed heterozygous rates.** Expected heterozygous estimates are the calculated directly from the G6PDd frequency in males: $Expected=2\times q\times(1-q)$. The observed heterozygous prevalence is estimated as the difference between the total observed G6PDd females and the expected proportion of homozygotes based on male G6PDd frequency: [$Observed=Female G6PDd prevalence-q^{2}$]. Only surveys sampling both sexes and with sample sizes ≥50 are included in these plots (n = 725). Panel B shows the 1:1 line (dashed red line) and the line of best fit (continuous blue line).

**S5.4 Modelling phenotypic G6PDd prevalence in females.**

When modelling the prevalence of G6PDd in females, the female population affected was considered as two distinct populations: homozygotes (whose prevalence was estimated directly from the population allele frequency ($q^{2}$), with the assumption that gene inheritance is in Hardy-Weinberg equilibrium) and heterozygotes (a proportion of whom will be phenotypically deficient). The discordance between genetic heterozygosity and phenotypic deficiency complicates the modelling of heterozygous prevalence significantly [[2](#_ENREF_2),[11](#_ENREF_11)]. In their most recent estimate of affected heterozygotes [[5](#_ENREF_5)], the WHO imposed a fixed threshold of 10% of heterozygotes being phenotypically deficient, as did the more recent study by Nkhoma et al. [[12](#_ENREF_12)]. However, the flexibility of the Bayesian model developed in this study meant that we could give the model the freedom to determine this threshold based directly on the input dataset. The expected variability in this threshold (Figure S5.3) indicated that imposing a single threshold was not the most appropriate method; instead, the observed variability supported the use of the spatially-variable deviance term, $h$, to moderate the number of heterozygotes likely to be deficient, as determined by the input dataset. A detailed description of these aspects of the model is given in Protocol S2.


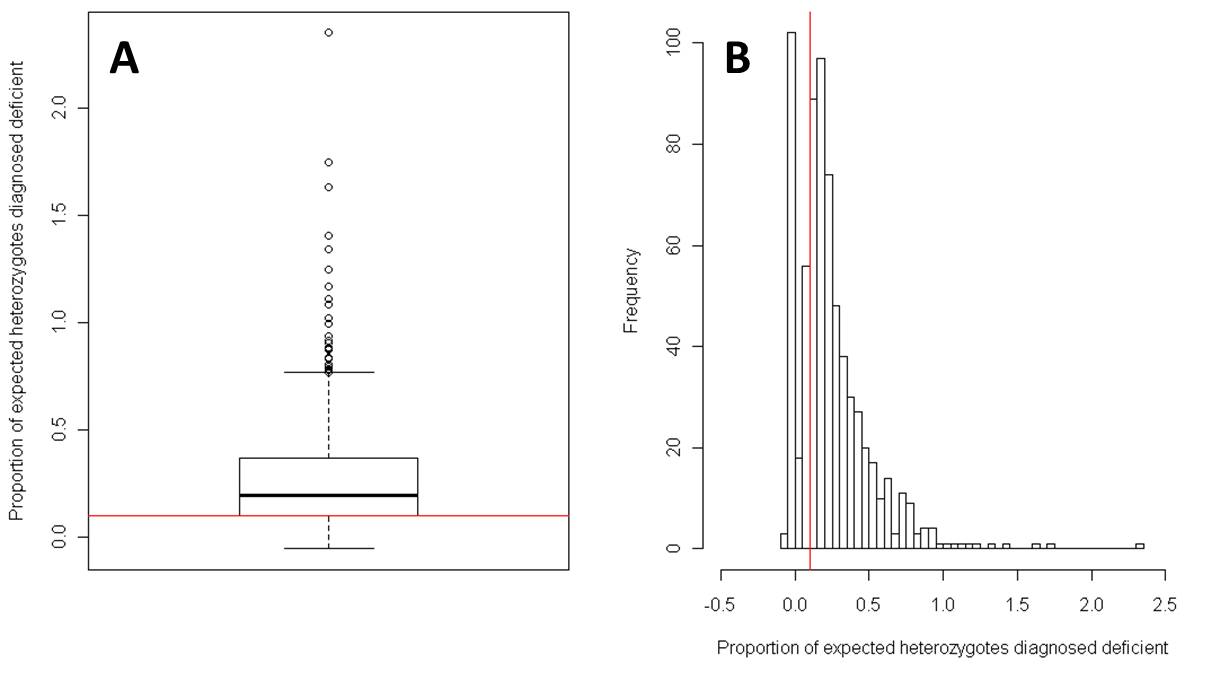
**Figure S5.3. Proportion of expected heterozygote females diagnosed as phenotypically deficient.** Expected heterozygotes are calculated from the corresponding prevalence of G6PDd males at each locality [$q$]. Plots show the proportion of genetic heterozygotes [$Expected=2\times q\times(1-q)$] diagnosed deficient [$Observed=Female G6PDd prevalence-q^{2}$], in boxplot (Panel A) and histogram (Panel B) plots. The red lines show the fixed 10% threshold used by the WHO in their calculations of heterozygote diagnosis [[5](#_ENREF_5)]. Summary statistics for the distribution shown: median 19.5% (IQR: 10.0% – 36.6%). Only surveys sampling both sexes and with sample sizes ≥50 are included in these plots (n = 725).

**S5.5 Maps of G6PDd in females and population estimates**

The spatial patterns of female G6PDd prevalence correspond to that for the allele frequency map (Figure 2B), and female population estimates, aggregated to national and regional areas, are given in Table 1 and Supplementary Table S1. The estimates of female homozygotes may be considered a highly conservative estimate of the overall number of females affected; in reality a proportion of heterozygotes will also share the G6PDd phenotype. Although G6PDd is frequently considered to be rare in females, it is clear from the assembled database and derived modelled population estimates that in many areas an important proportion of females will also be affected.

Figure S5.4 displays the estimated proportion of expected G6PDd heterozygotes (applying the Hardy-Weinberg equations to the median national summary estimates); while the WHO imposed a 10% threshold, the model developed here derived this threshold directly from the input data, with the flexibility for spatial variation. Across the predicted national predictions, a median proportion of 26.4% (IQR: 25.2-27.6) expected heterozygotes were predicted to be phenotypically deficient (Fig S5.4).

**
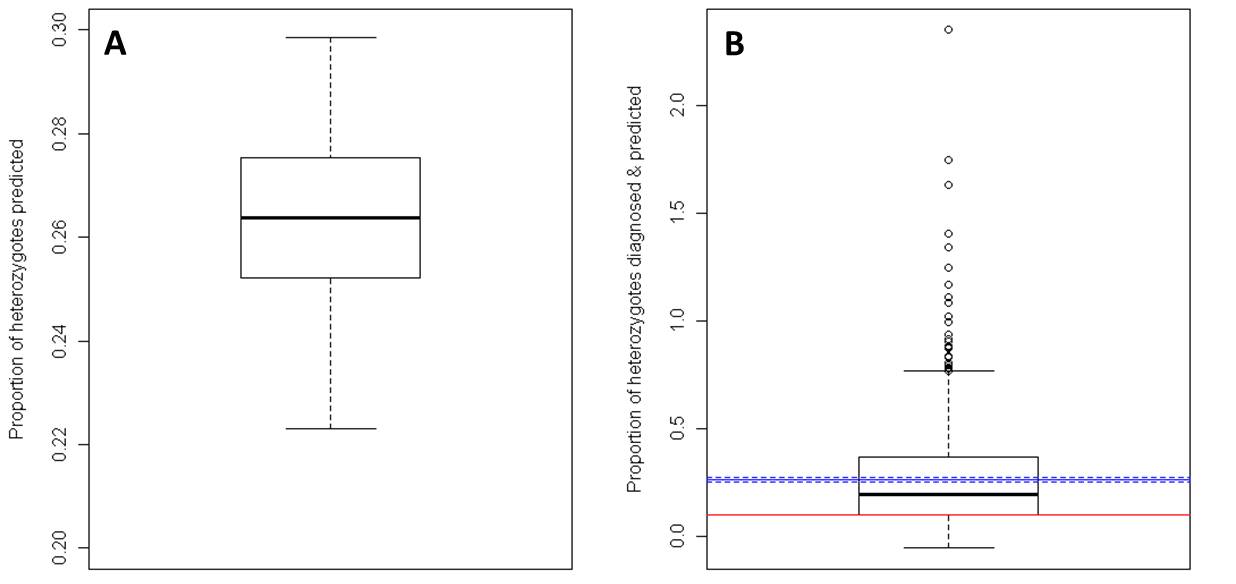
**

**Figure S5.4. Proportion of expected heterozygote females predicted by the model national population estimates.** Panel A plots the proportion of expected heterozygotes (derived from the model national estimates of allele frequency) predicted to be deficient; Panel B summarises the various heterozygote diagnostic cut-off limits: the boxplot data are of the input data points (n=725) [median value: 19.5% (IQR: 10.0-36.6)]; the red line represents the WHO [[5](#_ENREF_5)] and Nkhoma et al. [[12](#_ENREF_12)] 10% cut-off threshold, and the blue lines summarise the model’s national heterozygote population predictions [Panel A: median value: 26.4% (solid line) (IQR: 25.2-27.6; dashed lines)]

**S5.6 Improving the map of G6PDd in females**

Although we present population estimates of affected females, these estimates are largely dependent upon the input dataset of surveys, and are therefore vulnerable to the same limitations as those original population survey diagnoses. While most methods are considered broadly equivalent for determining male hemizygote and female homozygote expression (Protocol S1), skewed X-allele inactivation [[13](#_ENREF_13)] leaves the female heterozygous phenotype more ambiguous to diagnose and therefore harder to model [[2](#_ENREF_2),[14](#_ENREF_14)]. This relationship is spatially variable, and ill-defined; presumably dependent upon the severity of the genetic variant, the Lyonization skew (which determines the relative proportions of deficient and normal genes in the mosaic of G6PD erythrocytic expression [[6](#_ENREF_6)]), and, importantly, the sensitivity of the enzyme activity test used. It is well established that the proportion of heterozygotes identified from phenotypic tests is variable [[15](#_ENREF_15),[16](#_ENREF_16)]. However, in terms of primaquine use, we make the assumption here that the proportion of deficient heterozygotes who are at risk of significant clinical side-effects will all be identified by the majority of tests; and that the border-line cases who may or may not be diagnosed, depending on the tests, will be at lower risk of severe reactions and thus not of main concern to policy-makers. However, this assumes that enzyme activity correlates with clinical severity, which has not yet been demonstrated [[17](#_ENREF_17)].

The evidence-based, modelled estimates presented here for affected females are the first to be published, previous efforts having relied on a single pre-determined cut-off threshold [[5](#_ENREF_5),[12](#_ENREF_12)]. However, these estimates constitute only a first attempt; standardising the diagnostic methods used, and developing better datasets and models for predicting phenotypic heterozygote expression from allele frequency data are necessary to improve the estimates for affected females.

Finally, further clinical data are required to understand the association between local enzyme activity level and risk of haemolysis, as well as relating this information to relative risk associated with different G6PDd allele frequencies. The model here relies on the diagnostic tests having appropriate cut-off limits, but the model could be refined if a better understanding of these relationships were available.

**References**

1. Mason PJ, Bautista JM, Gilsanz F (2007) G6PD deficiency: the genotype-phenotype association. Blood Reviews 21: 267-283.

2. Peters AL, Van Noorden CJ (2009) Glucose-6-phosphate dehydrogenase deficiency and malaria: cytochemical detection of heterozygous G6PD deficiency in women. J Histochem Cytochem 57: 1003-1011.

3. Beutler E (1994) G6PD deficiency. Blood 84: 3613-3636.

4. Luzzatto L (2010) Glucose-6-phosphate dehydrogenase (G6PD) deficiency. In: Warrell DA, Cox TM, Firth JD, editors. Oxford Textbook of Medicine. Oxford: Oxford University Press.

5. WHO Working Group (1989) Glucose-6-phosphate dehydrogenase deficiency. Bull World Health Organ 67: 601-611.

6. Beutler E, Yeh M, Fairbanks VF (1962) The normal human female as a mosaic of X-chromosome activity: studies using the gene for G-6-PD-deficiency as a marker. Proc Natl Acad Sci U S A 48: 9-16.

7. Lyon MF (1961) Gene action in the X-chromosome of the mouse (*Mus musculus* L.). Nature 190: 372-373.

8. Luzzatto L, Notaro R (2001) Malaria. Protecting against bad air. Science 293: 442-443.

9. Minucci A, Giardina B, Zuppi C, Capoluongo E (2009) Glucose-6-phosphate dehydrogenase laboratory assay: How, when, and why? IUBMB Life 61: 27-34.

10. Kuwahata M, Wijesinghe R, Ho MF, Pelecanos A, Bobogare A, et al. (2010) Population screening for glucose-6-phosphate dehydrogenase deficiencies in Isabel Province, Solomon Islands, using a modified enzyme assay on filter paper dried bloodspots. Malar J 9: 223.

11. Cappellini MD, Fiorelli G (2008) Glucose-6-phosphate dehydrogenase deficiency. Lancet 371: 64-74.

12. Nkhoma ET, Poole C, Vannappagari V, Hall SA, Beutler E (2009) The global prevalence of glucose-6-phosphate dehydrogenase deficiency: a systematic review and meta-analysis. Blood Cells Mol Dis 42: 267-278.

13. Dobyns WB, Filauro A, Tomson BN, Chan AS, Ho AW, et al. (2004) Inheritance of most X-linked traits is not dominant or recessive, just X-linked. Am J Med Genet A 129A: 136-143.

14. Abdulrazzaq YM, Micallef R, Qureshi M, Dawodu A, Ahmed I, et al. (1999) Diversity in expression of glucose-6-phosphate dehydrogenase deficiency in females. Clin Genet 55: 13-19.

15. Ainoon O, Alawiyah A, Yu YH, Cheong SK, Hamidah NH, et al. (2003) Semiquantitative screening test for G6PD deficiency detects severe deficiency but misses a substantial proportion of partially-deficient females. Southeast Asian J Trop Med Public Health 34: 405-414.

16. Zaffanello M, Rugolotto S, Zamboni G, Gaudino R, Tato L (2004) Neonatal screening for glucose-6-phosphate dehydrogenase deficiency fails to detect heterozygote females. Eur J Epidemiol 19: 255-257.

17. Baird JK, Surjadjaja C (2011) Consideration of ethics in primaquine therapy against malaria transmission. Trends Parasitol 27: 11-16.
