# Supplementary material for: G6PD Deficiency Prevalence and Estimates of Affected Populations in Malaria Endemic Countries: A Geostatistical Model-Based Map
Source: PLoS Med. 2012 Nov 13;9(11):e1001339. doi: 10.1371/journal.pmed.1001339 (PMC3496665; doi:10.1371/journal.pmed.1001339)
Supplement: Protocol S6 — Developing an index of overall national-level risk from G6PD deficiency. (S6.1) G6PDd variants database. (S6.2) Generating an index of national-level risk from G6PDd. (S6.3) Generating an uncertainty index of the national-level risk index categories. (DOCX) [file pmed.1001339.s007.docx]

**Protocol S6. Developing an index of national-level risk from G6PD deficiency**

G6PD enzyme activity levels are variable due to heterogeneous biochemical and clinical characteristics, with over 186 underlying genetic variants [[1](#_ENREF_1)]. The relative enzyme activity of these variants ranges from virtual absence to higher than normal levels. We describe here the development of a coarse index for comparing the relative risk of G6PDd among countries, together with associated uncertainty measures. The primaquine sensitivity phenotype of many of these variants has yet to be determined [[2](#_ENREF_2)] so we have assumed that enzyme activity is inversely correlated with the severity of primaquine sensitivity. The index developed is a simple indicator of stratified risk developed for national planning. A more sophisticated approach would require more knowledge than is currently available on the relationships between genetic variants, biochemical variants and primaquine sensitivity.

**S6.1 G6PDd variants database**

We have assembled a database of reported occurrences of each G6PD variant in order to ascertain the combination of variants most commonly reported from each country. Following the same library assembly steps previously described (Protocol S1), bibliographic searches identified 2,237 published articles considered likely to contain relevant data and these were reviewed in detail. All reports of specific variants from named malaria endemic countries were recorded, generating a total of 1,468 positive occurrence reports of known variants, diagnosed with biochemical and molecular methods. Some biochemical variants are known to be genetic duplicates [[3](#_ENREF_3)]. To reconcile these with the unique, molecularly defined genetic variants, duplicated biochemical variants were aggregated based on the tables assembled by Mason et al. in 2007 [[4](#_ENREF_4)] and recently updated by Minucci et al. [[1](#_ENREF_1)].

A simple classification system of genetic variants [[5](#_ENREF_5)], endorsed by an expert WHO committee [[6](#_ENREF_6)], groups variants into five classes according to their residual enzyme activity levels, their clinical characteristics and their frequency within populations (polymorphic/sporadic) [[7](#_ENREF_7)] (Table S6.1). As rigorous primaquine sensitivity phenotypes are lacking for all but three variants [[2](#_ENREF_2)], we have used this classification system to separate ‘mild’ and ‘severe’ variants. Class I variants are associated with the most severe clinical symptoms of chronic haemolytic anaemia, however these variants are never polymorphic (≥1% prevalence) [[7](#_ENREF_7)]; as such, they are not of public health concern and were excluded from this analysis. Class IV and V do not express an enzyme deficiency, so are also irrelevant to this study.

| **Class** | **Degree of deficiency** | **Residual enzyme activity** | **Clinical characteristics** |
| --- | --- | --- | --- |
| I | Severe | Virtually none | Chronic non-spherocytic haemolytic anaemia (CNSHA) |
| II | Severe | <10% | Acute haemolytic anaemia |
| III | Moderate | 10 – 60% | Occasional haemolysis |
| IV | Normal | 60 – 150% | None |
| V | Increased activity | >150% | None |

**Table S6.1. Variant classifications**, based on residual enzyme activity levels. Table adapted from [[6](#_ENREF_6)] and [[8](#_ENREF_8)].

Class II and Class III variants express an enzymatic deficiency. The classifications are based on the general characteristics of the variants, with the reduction in enzyme activity of Class II variants tending to be more severe than with Class III variants, with the relative severity of clinical symptoms matching these. A series of databases [[1](#_ENREF_1),[5](#_ENREF_5),[7](#_ENREF_7),[9-12](#_ENREF_9)] report classifications of variants into these classes. Where several classifications were assigned to a single variant, these were reviewed on a case-by-case basis from the original publications and categorised according to either the most frequently reported classification of the variant, or as the most severe type when several classes were common. Of the genetically identified variants, classifications did not exist for a small number (n = 5 variants in our database: Dagua, Gond, Laibin, Yunan) of rarely reported variants (n = 9 total occurrences) which had to be excluded. A much more significant number of rarely reported variant occurrences (n = 152 variants) had to be excluded due to their poorly defined biochemical characteristics which precluded them from being reliably assigned to severity classes; these included diagnoses such as ‘Gd-1’, ‘B-slow’ and, for example, 21 different variants each reported only up to 3 times from populations in Papua New Guinea in the 1970-80s.

The final dataset included 932 positive occurrence data points, 527 of Class II variants and 405 of Class III variants, from 54 MECs. The variants reported from each country are listed in Table S3.

**Table S3. Reported observations of Class II and III G6PD variants from malaria endemic countries.** Only MECs for which data were available are listed (n = 54 out of 90 MECs). *The table is provided as a separate file.*

**S6.2 Generating an index of national-level risk from G6PDd**

The risk index was devised to account for both the national prevalence of G6PDd as estimated by our models and the relative severity of the variants from each country reported in our database, based on existing classifications [[1](#_ENREF_1),[5](#_ENREF_5),[7](#_ENREF_7),[9-12](#_ENREF_9)]. The prevalence score was based on national prevalence: ≤1% (score = 1); >1 - <10% (score = 2); ≥10% (score = 3). The variant severity score reflected the relative proportion of Class II and Class III variant reports. Score 1 (mildest severity) was given to countries from which only Class III variants were reported; score 2 (moderate severity) to countries where the majority of data points were Class III (≥⅔), but Class II variants were nevertheless reported; countries where Class II variants made up ≤⅓ of occurrences were scored 3 (severe). If no data were available from a country, a conservative approach was followed which took the highest score from any neighbouring country. Four island nations were lacking data: Haiti and Dominican Republic were scored 2 (moderate) based on the score of the large majority of surrounding mainland countries; and Madagascar was scored 1 (mild) based on Mozambique and all other central African countries; Mayotte was assigned score 3 (severe) in accordance with data from the nearby Comoros islands.

The index of overall risk was calculated by multiplying the prevalence and severity scores, resulting in six categories across a spectrum of risk from mild and rare G6PDd to common and severe G6PDd (Table S6.3). The resulting risk maps are shown in Figure S6.1 and Figure 4.

**­­­­­­­**

| **Severity index** | | **Variant severity** | | |
| --- | --- | --- | --- | --- |
|  |  | **Class III only** | **Class II uncommon** | **Class II common** |
| **National G6PDd prevalence** | **Rare**: ≤1% | Level 1 (n = 1) | Level 2 (n = 7) | Level 3 (n = 7) |
|  | **Common:** >1 - 10% | Level 2 (n = 13) | Level 4 (n = 15) | Level 5 (n = 20) |
|  | **High**: >10% | Level 3 (n = 20) | Level 5 (n = 5) | Level 6 (n = 11) |

**­­­­­­­**

**Table S6.3. Scoring table for determining an index of overall national-level risk from G6PDd,** accounting for the severity of the commonly reported mutations and the overall prevalence of deficiency.

**S6.3 Generating an uncertainty index of the national-level risk index categories**

As previously stated, interpretation of the G6PDd severity index as a predictor of the level of haemolytic risk among countries is contingent on the assumption of enzyme activity levels being inversely correlated with haemolytic risk. While this is generally assumed to be the case, exceptions have been reported, for example an Iranian boy with 19.5% enzyme activity required transfusion following a single 45mg dose of primaquine [[13](#_ENREF_13)]; similarly, a five year old heterozygous A-variant female in Tanzania, considered a very mild genotype, suffered severe anaemia (Hb level <5 g/dL) following a single 15mg dose of primaquine [[14](#_ENREF_14)]. While these cases are exceptions, they do emphasise the universal need for careful patient monitoring following primaquine ingestion. Making predictions about haemolytic risk is therefore inherently difficult, but indicators of relative uncertainty based on use of the
**
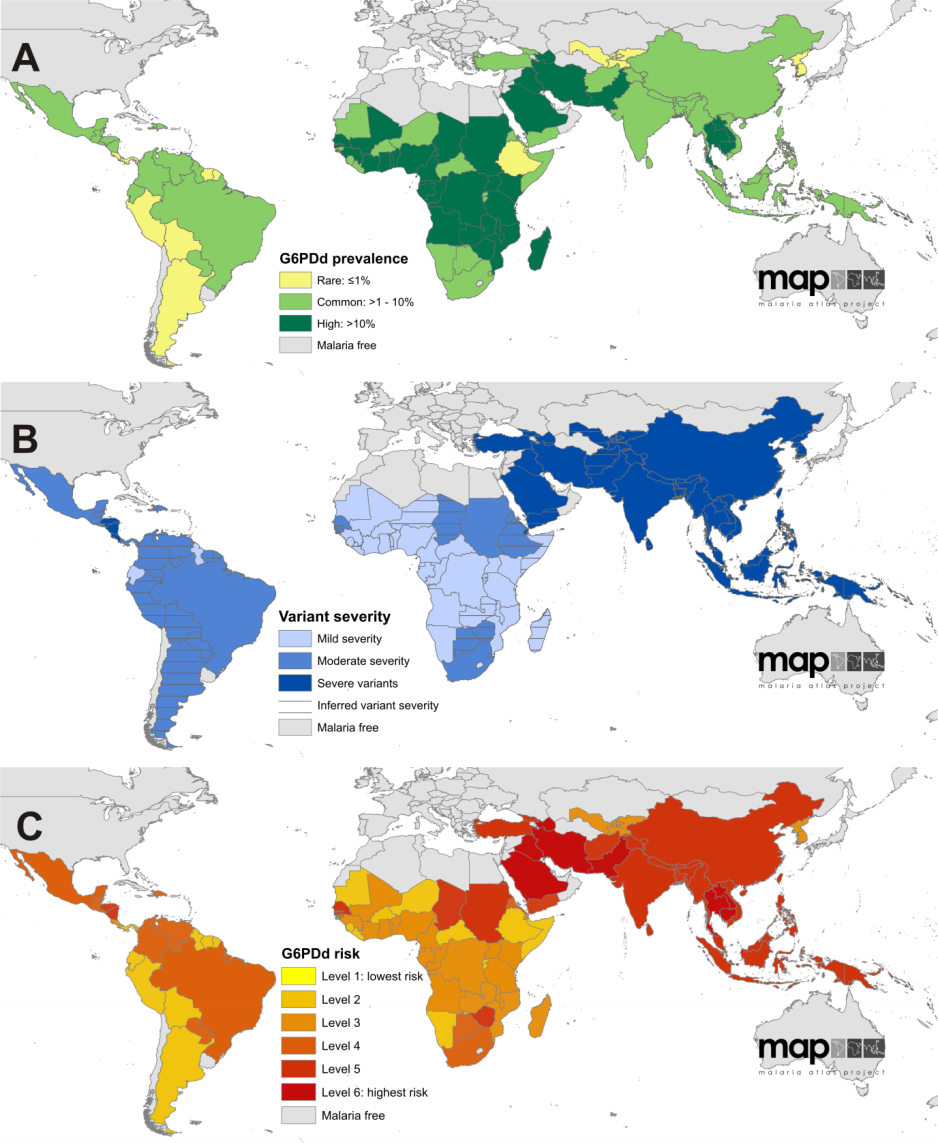
Figure S6.1. National-level prevalence scores, variant severity scores and the culminating index of overall national-level risk from G6PDd.** Panel A shows the scored prevalence estimates (score = 1: if national prevalence is estimated as ≤1%; score = 2 if national prevalence is estimated as >1- 10%; and score = 3 if the national prevalence is >10%); Panel B gives the three variant severity scores: lowest severity (score = 1) for countries with only Class III G6PD variants, moderate variant severity (score = 2) for countries where a minority (≤ ⅓) of Class II prevailed among records of Class III variants; and the most severe (score = 3) for countries where Class II G6PD variants were common (>⅓ records). Panel C shows the final six indices of overall national-level risk from G6PDd: the scores in Panels A and B were multiplied.

**
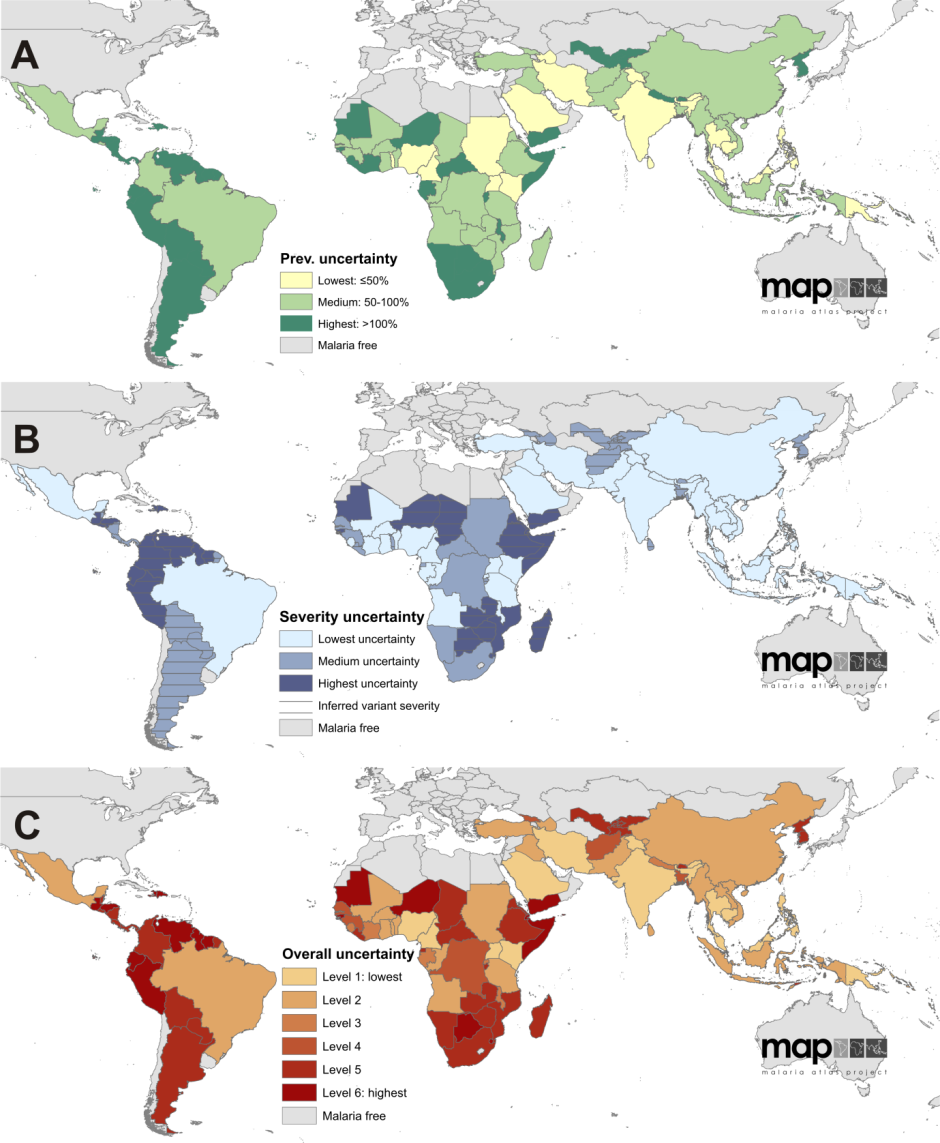
 Figure S6.2. National-level scores of prevalence uncertainty, variant severity score uncertainty and overall uncertainty in national-level risk from G6PD.** Panel A shows the stratified prevalence uncertainty based on the proportion of IQR relative to median predictions (score = 1: if the IQR of the prevalence prediction was ≤50% of median prediction; score = 2: if the IQR was 50-100% of median prevalence prediction; score = 3 if the IQR was >100% of the median prevalence prediction for that country); Panel B gives the estimated variant severity uncertainty: scores were determined by both the number of data points in each country and the local heterogeneity in variant severity scores (fully described in Section S6.3); and Panel C maps the final scores from multiplying Panels A and B into an index of overall uncertainty in the national-level classifications (Table S6.5).

available data can be derived. This uncertainty around clinical outcomes is of course
additional to the uncertainty in the data used to derive the index; an attempt is made here to quantify the uncertainty from these latter two data sources to support regional-level comparisons of relative risks from G6PDd.

Using the same framework as for the risk index, scores were devised to account for the level of uncertainty with which the risk classifications were made. These accounted for uncertainty in the national prevalence prediction as well as uncertainty in the estimate of local variant severity. Uncertainty in the prevalence estimate was scored as the size of the IQR around the prevalence prediction relative to the median estimate. Countries where the prediction was most certain (IQR ≤50% median estimate) were given score 1; if the IQR was 50-100% the size of the median estimate, countries were scored 2; finally a score of 3 was given when the IQR was >100% the size of the median estimate. IQR values for all national-level prevalence estimates are given in Table S1.

To stratify uncertainty in the variant severity score we used two factors; the number of occurrence points within each country, and the local heterogeneity in variant scores. If all neighbouring countries had the same severity scores, then uncertainty was decreased. Conversely, nearby heterogeneity increased uncertainty. The categories for numbers of data points per country were defined as: ≥3 data points (score = 1), 1-2 data points (score = 2), and 0 data points (score = 3). Heterogeneity was scored as Low (score = 1) if a country’s variant severity score was the same as all neighbouring countries, Medium (score = 2) if a country’s variant severity score was the same as more neighbours than not, and High (score = 3) if most neighbours had a different variant severity score. Based on these two scores, each country was allocated an overall score from 1 to 3 for variant severity uncertainty using Table S6.4.

| **Variant severity scoring uncertainty** | | **Number of data points per country** | | |
| --- | --- | --- | --- | --- |
|  |  | **3+**  (score = 1) | **1 – 2**  (score = 2) | **0**  (score = 3) |
| **Nearby heterogeneity** | **Low**  (score = 1) | Low uncertainty  (n = 22) | Low uncertainty  (n = 10) | Medium uncertainty (n=22) |
|  | **Medium**  (score = 2) | Low uncertainty  (n = 7) | Medium uncertainty  (n = 4) | High uncertainty  (n = 10) |
|  | **High**  (score = 3) | Medium uncertainty  (n = 5) | High uncertainty  (n = 6) | High uncertainty  (n = 13) |

**­­­­­**

**Table S6.4. Scoring table for determining the uncertainty of variant severity scores**, based on numbers of data points per country, and regional heterogeneity in variant severity scores. These uncertainty classes in the variant severity scores are mapped in Figure S6.2B.

The variant severity uncertainty scores were combined with the prevalence uncertainty scores for each country in a multiplicative table (Table S6.5) to generate a final uncertainty score for each country, as mapped in Figure S6.2.

| **Overall uncertainty index** | | **Reliability of severity score** | | |
| --- | --- | --- | --- | --- |
|  |  | **Low uncertainty** | **Medium uncertainty** | **High uncertainty** |
| **Prevalence uncertainty**  (IQR/Median) | **Low**: 0 - 50% | Level 1 (n = 13) | Level 2 (n = 4) | Level 3 (n = 0) |
|  | **Medium**: 50 - 100% | Level 2 (n = 19) | Level 4 (n =6) | Level 5 (n = 9) |
|  | **High**: >100% | Level 3 (n = 7) | Level 5 (n = 21) | Level 6 (n = 20) |

**Table S6.5. Scoring table for determining the index of overall uncertainty in the national-level risk classifications.** Final categories of the risk scores are shown, with total number of MECs belonging to each category. These are mapped in Figure S6.2C.

**References**

1. Minucci A, Moradkhani K, Hwang MJ, Zuppi C, Giardina B, et al. (2012) Glucose-6-phosphate dehydrogenase (G6PD) mutations database: review of the "old" and update of the new mutations. Blood Cells Mol Dis 48: 154-165.

2. Baird JK, Surjadjaja C (2011) Consideration of ethics in primaquine therapy against malaria transmission. Trends Parasitol 27: 11-16.

3. Cappellini MD, Martinez di Montemuros F, De Bellis G, Debernardi S, Dotti C, et al. (1996) Multiple G6PD mutations are associated with a clinical and biochemical phenotype similar to that of G6PD Mediterranean. Blood 87: 3953-3958.

4. Mason PJ, Bautista JM, Gilsanz F (2007) G6PD deficiency: the genotype-phenotype association. Blood Reviews 21: 267-283.

5. Yoshida A, Beutler E, Motulsky AG (1971) Human glucose-6-phosphate dehydrogenase variants. Bull World Health Organ 45: 243-253.

6. WHO Working Group (1989) Glucose-6-phosphate dehydrogenase deficiency. Bull World Health Organ 67: 601-611.

7. Luzzatto L, Mehta A, Vulliamy TJ (2001) Glucose-6-phosphate dehydrogenase deficiency. In: Scriver CR, Beaudet AL, Sly WS, Valle D, editors. The metabolic and molecular bases of inherited disease, 8th ed. New York: McGraw-Hill Inc. pp. 4517-4553.

8. Cappellini MD, Fiorelli G (2008) Glucose-6-phosphate dehydrogenase deficiency. Lancet 371: 64-74.

9. Dr Andrew C. R. Martin's Group Andrew C. R. Martin's Bioinformatics Group at UCL.

10. Kwok CJ, Martin AC, Au SW, Lam VM (2002) G6PDdb, an integrated database of glucose-6-phosphate dehydrogenase (G6PD) mutations. Hum Mutat 19: 217-224.

11. Vulliamy T, Luzzatto L, Hirono A, Beutler E (1997) Hematologically Important Mutations: Glucose-6-Phosphate Dehydrogenase. Blood Cells Mol Dis 23: 302-313.

12. Beutler E (1993) Study of glucose-6-phosphate dehydrogenase: history and molecular biology. Am J Hematol 42: 53-58.

13. Ziai M, Amirhakimi GH, Reinhold JG, Tabatabee M, Gettner ME, et al. (1967) Malaria prophylaxis and treatment in G-6-PD deficiency. An observation on the toxicity of primaquine and chloroquine. Clin Pediatr (Phila) 6: 242-243.

14. Shekalaghe SA, ter Braak R, Daou M, Kavishe R, van den Bijllaardt W, et al. (2010) In Tanzania, hemolysis after a single dose of primaquine coadministered with an artemisinin is not restricted to glucose-6-phosphate dehydrogenase-deficient (G6PD A-) individuals. Antimicrob Agents Chemother 54: 1762-1768.
