# Supplementary material for: G6PD Deficiency Prevalence and Estimates of Affected Populations in Malaria Endemic Countries: A Geostatistical Model-Based Map
Source: PLoS Med. 2012 Nov 13;9(11):e1001339. doi: 10.1371/journal.pmed.1001339 (PMC3496665; doi:10.1371/journal.pmed.1001339)
Supplement: Table S1 — National-level demographic metrics and G6PDd allele frequency and population estimates. (PDF) [file pmed.1001339.s008.pdf]

Supplementary Tables

Supplementary Table S1: National-level demographic metrics and G6PDd allele frequency and population estimates (in 1,000s)

Country	Total pop[1,2]	Sex-ratio[3]	Surveys	G6PDd allele freq (IQR)[4]		G6PDd males[1] (IQR)[4]		Homozygotes[1,5]	G6PDd females[1] (IQR)[4]	
**African MECs[6]**										
Angola	18,994	98.1	2	15.3%	(10.4 - 22.2)	1,435	(980 - 2,091)	223	947	(621 - 1,468)
Benin	9,219	97.3	0	23.0%	(17.0 - 30.1)	1,044	(772 - 1,366)	246	706	(490 - 984)
Botswana†	1,977	101.7	2	3.6%	(2.0 - 6.5)	36	(20 - 65)	1	19	(10 - 37)
Burkina Faso	16,250	98.5	0	9.4%	(5.6 - 15.0)	757	(455 - 1,206)	72	452	(256 - 772)
Burundi	8,519	96.3	0	7.2%	(3.3 - 15.2)	301	(137 - 635)	23	169	(73 - 394)
Cameroon	19,957	99.7	8	12.5%	(9.9 - 15.5)	1,248	(990 - 1,543)	157	750	(568 - 967)
Cape Verde†	513	98.0	0	0.1%	(0.0 - 0.5)	0	(0 - 1)	0	0	(0 - 1)
Central African Republic	4,506	97.1	0	9.2%	(4.7 - 17.3)	203	(103 - 383)	19	126	(59 - 258)
Chad	11,509	98.9	0	13.4%	(8.5 - 20.2)	767	(488 - 1,155)	104	496	(294 - 799)
Comoros	691	101.4	0	14.0%	(5.8 - 30.4)	49	(20 - 106)	7	29	(10 - 71)
Congo	3,760	100.2	1	22.5%	(17.3 - 29.6)	424	(326 - 557)	95	277	(205 - 387)
Cote d'Ivoire	21,571	103.9	0	15.0%	(8.5 - 25.5)	1,654	(931 - 2,800)	240	991	(514 - 1,843)
Democratic Republic of the Congo	67,829	98.9	6	19.2%	(14.7 - 25.1)	6,488	(4,974 - 8,459)	1,261	4,425	(3,270 - 6,066)
Djibouti	879	100.1	0	0.8%	(0.3 - 2.7)	4	(1 - 12)	0	2	(1 - 6)
Equatorial Guinea	693	105.2	0	11.4%	(6.1 - 20.1)	40	(22 - 72)	4	22	(11 - 43)
Eritrea	5,204	97.1	1	4.0%	(2.7 - 6.1)	103	(68 - 157)	4	56	(36 - 88)
Ethiopia	84,996	99.1	6	1.0%	(0.7 - 1.5)	422	(281 - 642)	4	218	(142 - 338)
Gabon	1,501	100.6	0	12.3%	(6.0 - 23.4)	92	(45 - 176)	11	56	(25 - 117)
Ghana	24,339	103.6	2	19.6%	(14.2 - 27)	2,429	(1,764 - 3,341)	460	1,498	(1,031 - 2,194)
Guinea	10,323	102.1	0	11.7%	(7.4 - 18.8)	611	(385 - 982)	70	357	(214 - 621)
Guinea-Bissau	1,647	98.3	0	8.4%	(4.4 - 15.3)	68	(36 - 124)	6	39	(19 - 76)
Kenya	40,847	99.8	45	11.3%	(9.2 - 13.7)	2,310	(1,880 - 2,805)	262	1,377	(1,092 - 1,725)
Liberia	4,102	101.0	1	9.5%	(5.2 - 16.9)	196	(107 - 348)	19	112	(58 - 216)
Madagascar	20,146	99.4	1	19.4%	(11.5 - 30.3)	1,952	(1,154 - 3,046)	382	1,301	(711 - 2,212)
Malawi	15,690	100.1	0	20.8%	(10.2 - 36.4)	1,629	(799 - 2,858)	338	1,067	(468 - 2,105)
Mali	13,362	99.8	3	12.2%	(8.6 - 17.3)	813	(574 - 1,156)	99	499	(335 - 751)
Mauritania	3,359	101.0	0	9.6%	(4.6 - 18.5)	162	(78 - 312)	15	95	(43 - 205)
Mayotte	199	99.5	0	12.4%	(3.9 - 32.7)	12	(4 - 32)	2	7	(2 - 22)

Country	Total pop[1,2]	Sex-ratio[3]	Surveys	G6PDd allele freq (IQR)[4]		G6PDd males[1] (IQR)[4]		Homozygotes[1,5]	G6PDd females[1] (IQR)[4]	
Mozambique	23,418	94.8	4	21.1%	(14.7 - 29.8)	2,404	(1,670 - 3,394)	535	1,703	(1,112 - 2,587)
Namibia†	2,212	98.7	5	2.8%	(1.8 - 4.6)	31	(20 - 51)	1	17	(11 - 29)
Niger	15,885	101.2	0	5.3%	(2.6 - 10.3)	426	(211 - 819)	22	236	(111 - 497)
Nigeria	158,255	102.5	6	16.9%	(14.1 - 20.2)	13,515	(11,317 - 16,185)	2,224	8,464	(6,898 - 10,477)
Rwanda	10,277	96.4	0	5.8%	(3.3 - 10.1)	294	(169 - 509)	18	163	(90 - 298)
Sao Tome and Principe†	165	98.1	0	7.4%	(2.3 - 20.8)	6	(2 - 17)	0	3	(1 - 11)
Senegal	12,866	98.4	1	15.1%	(11.3 - 20.3)	966	(720 - 1,295)	148	598	(424 - 847)
Sierra Leone	5,837	95.5	0	7.9%	(3.4 - 17.0)	226	(98 - 485)	19	132	(53 - 313)
Somalia	9,359	98.4	0	3.1%	(1.2 - 7.7)	145	(56 - 356)	5	80	(29 - 215)
South Africa†	50,523	98.1	0	3.3%	(1.8 - 6.2)	830	(438 - 1,563)	28	482	(242 - 960)
Sudan	43,204	101.5	8	15.3%	(12.7 - 18.2)	3,322	(2,763 - 3,964)	499	2,118	(1,691 - 2,623)
Swaziland†	1,195	96.7	0	8.7%	(4.6 - 15.5)	51	(27 - 91)	5	29	(15 - 57)
The Gambia	1,751	97.6	2	11.5%	8.1 - 15.9)	99	(70 - 138)	12	58	(39 - 85)
Togo	6,774	98.1	3	21.2%	(16.7 - 26.6)	712	(560 - 893)	154	463	(346 - 616)
Uganda	33,798	99.9	12	14.5%	(12.8 - 16.5)	2,457	(2,162 - 2,785)	358	1,468	(1,263 - 1,706)
United Republic of Tanzania	45,028	99.8	10	16.4%	(11.9 - 22.3)	3,685	(2,671 - 5,019)	605	2,372	(1,643 - 3,431)
Zambia	13,254	100.5	1	21.0%	(14.6 - 29.4)	1,393	(971 - 1,950)	291	923	(606 - 1,393)
Zimbabwe	12,645	97.2	2	14.8%	(11.2 - 19.4)	924	(698 - 1,212)	141	586	(421 - 808)
**American MECs[6]**										
Argentina†	40,668	95.8	1	0.9%	(0.5 - 1.6)	169	(98 - 313)	2	92	(51 - 181)
Belize†	313	97.3	0	2.2%	(0.9 - 5.1)	3	(1 - 8)	0	2	(1 - 4)
Bolivia	9,995	99.5	0	0.2%	(0.1 - 0.8)	11	(3 - 41)	0	6	(2 - 21)
Brazil	195,453	96.9	14	4.8%	(3.6 - 6.5)	4,647	(3,501 - 6,213)	232	2,758	(1,994 - 3,897)
Colombia	46,305	96.8	2	4.9%	(3.4 - 7.3)	1,118	(764 - 1,667)	57	638	(419 - 1,008)
Costa Rica†	4,640	103.1	3	0.4%	(0.2 - 1.0)	9	(4 - 23)	0	4	(2 - 11)
Dominican Republic†	10,225	100.7	0	3.0%	(0.9 - 10.0)	154	(44 - 511)	5	79	(22 - 288)
Ecuador	13,775	100.3	2	4.2%	(2.4 - 7.5)	292	(166 - 519)	12	157	(87 - 294)
El Salvador†	6,201	90.5	1	3.3%	(2.4 - 4.8)	98	(69 - 140)	4	56	(39 - 81)
French Guiana	231	100.3	0	0.7%	(0.3 - 1.6)	1	(0 - 2)	0	0	(0 - 1)
Guatemala	14,378	95.1	0	2.7%	(1.5 - 5.1)	189	(103 - 355)	5	102	(54 - 199)
Guyana	761	100.9	0	3.0%	(1.4 - 6.4)	11	(5 - 25)	0	6	(3 - 13)
Haiti	10,188	98.4	0	5.2%	(1.9 - 13.2)	261	(94 - 665)	14	141	(48 - 395)
Honduras	7,609	99.9	0	2.9%	(1.5 - 5.8)	111	(55 - 219)	3	58	(28 - 118)

Country	Total pop[1],[2]	Sex-ratio[3]	Surveys	G6PDd allele freq (IQR)[4]		G6PDd males[1] (IQR)[4]		Homozygotes[1],[5]	G6PDd females[1] (IQR)[4]	
Mexico†	110,568	97.3	25	1.0%	(0.8 - 1.3)	555	(430 - 733)	6	291	(222 - 387)
Nicaragua†	5,822	97.9	0	1.5%	(0.6 - 3.6)	43	(18 - 103)	1	22	(9 - 56)
Panama†	3,508	101.5	0	0.9%	(0.4 - 2.5)	16	(6 - 44)	0	8	(3 - 22)
Paraguay†	6,462	101.8	0	3.2%	(1.1 - 8.8)	105	(34 - 288)	3	54	(17 - 163)
Peru	29,493	100.4	3	0.2%	(0.1 - 0.6)	33	(13 - 84)	0	17	(6 - 43)
Suriname	524	100.6	5	0.7%	(0.4 - 1.3)	2	(1 - 3)	0	1	(1 - 2)
Venezuela	29,044	100.7	0	8.6%	(4.0 - 18.0)	1,251	(583 - 2,617)	107	732	(316 - 1,701)
**Eurasian MECs[6]**										
Afghanistan	29,117	107.2	5	7.4%	(5.6 - 9.8)	1,115	(845 - 1,470)	77	599	(436 - 833)
Azerbaijan†	8,932	97.8	17	10.2%	(8.9 - 11.7)	452	(393 - 518)	47	267	(228 - 314)
Bangladesh	164,424	102.6	0	3.8%	(2.4 - 5.9)	3,168	(2,002 - 4,942)	117	1,624	(1,007 - 2,636)
Bhutan†	723	112.5	0	5.9%	(3.6 - 9.6)	23	(14 - 37)	1	11	(6 - 18)
Cambodia	15,056	95.8	11	14.3%	(11.8 - 17.2)	1,055	(871 - 1,268)	158	653	(522 - 811)
China†	1,381,796	108.0	11	4.7%	(3.5 - 6.8)	33,675	(25,014 - 48,717)	1,464	18,555	(13,427 - 27,859)
Dem People's Rep of Korea†	23,963	96.3	0	0.1%	(0.0 - 0.4)	10	(2 - 42)	0	5	(1 - 22)
Georgia†	4,221	89.0	2	1.1%	(0.7 - 1.7)	21	(14 - 33)	0	12	(8 - 19)
India	1,209,105	106.8	45	8.0%	(6.9 - 9.3)	50,009	(43,246 - 57,985)	3,748	27,708	(23,452 - 32,947)
Indonesia	232,544	99.5	33	7.1%	(5.3 - 9.4)	8,204	(6,180 - 10,901)	583	4,856	(3,484 - 6,756)
Iran (Islamic Republic of)†	75,084	103.0	12	11.8%	(9.9 - 14.1)	4,510	(3,788 - 5,356)	518	2,661	(2,186 - 3,256)
Iraq†	31,443	100.6	1	10.6%	(8.1 - 13.5)	1,669	(1,279 - 2,130)	176	970	(720 - 1,297)
Korea, Rep of†	48,517	99.4	0	0.2%	(0.1 - 0.6)	50	(17 - 145)	0	24	(8 - 72)
Kyrgyzstan†	5,545	97.4	0	0.3%	(0.1 - 1.2)	9	(3 - 33)	0	5	(1 - 17)
Lao People's Democratic Republic	6,434	99.6	1	15.6%	(11.6 - 20.5)	500	(372 - 657)	78	315	(224 - 437)
Malaysia†	27,949	103.0	12	8.0%	(6.6 - 9.6)	1,129	(942 - 1,359)	87	627	(506 - 782)
Myanmar	50,503	97.2	7	6.1%	(4.1 - 9.3)	1,523	(1,028 - 2,309)	96	880	(577 - 1,384)
Nepal	29,950	98.4	0	5.3%	(2.9 - 9.4)	786	(436 - 1,390)	42	434	(230 - 814)
Pakistan	189,875	103.4	8	15.0%	(10.8 - 20.4)	14,495	(10,393 - 19,648)	2,106	8,900	(5,984 - 13,089)
Papua New Guinea	6,887	104.1	34	7.4%	(6.0 - 9.3)	261	(212 - 325)	19	143	(114 - 185)
Philippines†	93,617	100.7	636	2.5%	(2.4 - 2.5)	1,151	(1,117 - 1,187)	28	580	(556 - 604)
Saudi Arabia†	26,207	124.0	19	12.4%	(10.4 - 14.9)	1,794	(1,511 - 2,161)	179	899	(741 - 1,113)
Solomon Islands†	536	107.1	41	22.3%	(15.7 - 30.9)	62	(43 - 86)	13	38	(25 - 56)
Sri Lanka†	20,410	97.5	84	2.9%	(2.6 - 3.3)	291	(258 - 331)	9	161	(140 - 188)
Tajikistan†	7,078	96.9	0	0.8%	(0.4 - 1.9)	29	(12 - 66)	0	15	(6 - 34)

Country	Total pop[1,2]	Sex-ratio[3]	Surveys	G6PDd allele freq (IQR)[4]		G6PDd males[1] (IQR)[4]		Homozygotes[1,5]	G6PDd females[1] (IQR)[4]	
Thailand†	68,141	96.7	20	13.6%	(11.9 - 15.5)	4,544	(3,981 - 5,188)	638	2,830	(2,428 - 3,312)
Timor-Leste	1,168	104.0	0	5.0%	(2.5 - 9.7)	29	(15 - 58)	1	15	(7 - 31)
Turkey†	75,699	99.5	79	3.8%	(3.0 - 4.9)	1,437	(1,128 - 1,850)	55	783	(601 - 1,034)
Uzbekistan†	27,790	98.8	0	1.0%	(0.4 - 2.4)	132	(53 - 330)	1	68	(27 - 179)
Vanuatu†	246	103.8	17	8.0%	(6.9 - 9.3)	10	(9 - 12)	1	5	(4 - 6)
Viet Nam†	89,016	97.7	6	8.9%	(6.0 - 13.9)	3,902	(2,625 - 6,102)	354	2,310	(1,488 - 3,922)
Yemen	24,324	101.3	0	4.6%	(1.9 - 10.9)	565	(238 - 1,329)	26	308	(121 - 781)

[1] All population estimates are in 1,000s

[2] Source: GRUMP-adjusted UN population projected population estimates for 2010; World Population Prospects, the 2008 Revision.

[3] Number of males per 100 females in 2010. Source: United Nations Department of Economics and Social Affairs (2011) World Population Prospects, the 2010 Revision. http://esa.un.org/unpd/wpp/Excel-Data/population.htm.

[4] Interquartile range of the posterior predictive distribution for each G6PDd population estimate

[5] Homozygous female estimates are derived directly from the median allele frequency estimates, so are not given with modelled IQRs

[6] Malaria Endemic Countries (Protocol S1.6)

† Countries targetting malaria elimination
