# Supplementary material for: G6PD Deficiency Prevalence and Estimates of Affected Populations in Malaria Endemic Countries: A Geostatistical Model-Based Map
Source: PLoS Med. 2012 Nov 13;9(11):e1001339. doi: 10.1371/journal.pmed.1001339 (PMC3496665; doi:10.1371/journal.pmed.1001339)
Supplement: Table S2 — National areal prediction summary statistics and Monte Carlo standard error (SE) for each model output. (PDF) [file pmed.1001339.s009.pdf]

Supplementary Table S2: National areal prediction summary statistics and Monte Carlo standard errors (SE) for each model output

	Allele frequency (SE)				G6PDd male population (SE)				G6PDd female population (SE)			
**Country**	**Mean**	**Q25%**	**Median**	**Q75%**	**Mean**	**Q25%**	**Median**	**Q75%**	**Mean**	**Q25%**	**Median**	**Q75%**
**African MECs**												
Angola	17.4 (0.06) %	10.4 (0.10) %	15.3 (0.10) %	22.2 (0.10) %	1,635 (5.9)	980 (9.6)	1,435 (9.6)	2,091 (2.1)	1,143 (4.6)	621 (7.1)	947 (6.6)	1,468 (11.0)
Benin	24.0 (0.08) %	17.0 (0.11) %	23.0 (0.07) %	30.1 (0.13) %	1,091 (3.5)	772 (5.0)	1,044 (3.3)	1,366 (1.4)	769 (3.0)	490 (3.1)	706 (3.2)	984 (6.4)
Botswana	5.2 (0.04) %	2.0 (0.02) %	3.6 (0.04) %	6.5 (0.07) %	52 (0.3)	20 (0.2)	36 (0.4)	65 (0.1)	30 (0.2)	10 (0.1)	19 (0.2)	37 (0.4)
Burkina Faso	11.4 (0.08) %	5.6 (0.04) %	9.4 (0.06) %	15.0 (0.21) %	920 (6.7)	455 (3.3)	757 (4.9)	1,206 (1.2)	592 (5.0)	256 (2.4)	452 (3.1)	772 (13.0)
Burundi	11.3 (0.12) %	3.3 (0.07) %	7.2 (0.09) %	15.2 (0.22) %	473 (5.1)	137 (2.8)	301 (3.9)	635 (0.6)	314 (3.9)	73 (1.4)	169 (2.5)	394 (6.2)
Cameroon	13.0 (0.04) %	9.9 (0.05) %	12.5 (0.05) %	15.5 (0.06) %	1,297 (4.3)	990 (5.2)	1,248 (4.8)	1,543 (1.5)	798 (3.8)	568 (3.0)	750 (3.8)	967 (5.6)
Cape Verde	1.0 (0.03) %	0.0 (0.00) %	0.1 (0.00) %	0.5 (0.01) %	3 (0.1)	0 (0.0)	0 (0.0)	1 (0.0)	2 (0.1)	0 (0.0)	0 (0.0)	1 (0.0)
Central African Rep	12.5 (0.13) %	4.7 (0.09) %	9.2 (0.12) %	17.3 (0.22) %	277 (2.8)	103 (2.0)	203 (2.6)	383 (0.4)	192 (2.2)	59 (1.2)	126 (1.9)	258 (4.2)
Chad	15.4 (0.13) %	8.5 (0.09) %	13.4 (0.15) %	20.2 (0.25) %	882 (7.2)	488 (5.3)	767 (8.3)	1,155 (1.2)	605 (6.1)	294 (3.8)	496 (6.5)	799 (12.6)
Comoros	20.6 (0.15) %	5.8 (0.14) %	14.0 (0.17) %	30.4 (0.24) %	72 (0.5)	20 (0.5)	49 (0.6)	106 (0.1)	50 (0.4)	10 (0.2)	29 (0.4)	71 (0.8)
Congo	24.2 (0.12) %	17.3 (0.14) %	22.5 (0.12) %	29.6 (0.17) %	456 (2.2)	326 (2.6)	424 (2.3)	557 (0.6)	312 (1.7)	205 (1.7)	277 (1.9)	387 (2.7)
Cote d'Ivoire	18.6 (0.19) %	8.5 (0.10) %	15.0 (0.23) %	25.5 (0.31) %	2,045 (21.3)	931 (11.3)	1,654 (24.8)	2,800 (2.8)	1,358 (16.5)	514 (7.9)	991 (16.9)	1,843 (26.3)
Dem Rep of the Congo	20.5 (0.11) %	14.7 (0.12) %	19.2 (0.11) %	25.1 (0.15) %	6,928 (35.8)	4,974 (41.5)	6,488 (37.0)	8,459 (8.5)	4,891 (28.5)	3,270 (30.6)	4,425 (33.1)	6,066 (37.1)
Djibouti	2.9 (0.04) %	0.3 (0.01) %	0.8 (0.02) %	2.7 (0.06) %	13 (0.2)	1 (0.0)	4 (0.1)	12 (0.0)	7 (0.1)	1 (0.0)	2 (0.0)	6 (0.1)
Equatorial Guinea	14.8 (0.09) %	6.1 (0.09) %	11.4 (0.12) %	20.1 (0.09) %	53 (0.3)	22 (0.3)	40 (0.4)	72 (0.1)	33 (0.2)	11 (0.2)	22 (0.2)	43 (0.4)
Eritrea	4.8 (0.04) %	2.7 (0.03) %	4.0 (0.04) %	6.1 (0.07) %	124 (1.1)	68 (0.8)	103 (1.1)	157 (0.2)	70 (0.7)	36 (0.4)	56 (0.6)	88 (1.1)
Ethiopia	1.2 (0.01) %	0.7 (0.01) %	1.0 (0.01) %	1.5 (0.01) %	515 (4.9)	281 (2.4)	422 (3.0)	642 (0.6)	275 (3.0)	142 (1.3)	218 (1.6)	338 (3.2)
Gabon	16.8 (0.15) %	6.0 (0.06) %	12.3 (0.18) %	23.4 (0.31) %	126 (1.2)	45 (0.4)	92 (1.3)	176 (0.2)	87 (1.0)	25 (0.2)	56 (0.9)	117 (1.6)
Ghana	21.5 (0.08) %	14.2 (0.11) %	19.6 (0.09) %	27.0 (0.10) %	2,664 (10.4)	1,764 (13.4)	2,429 (11.0)	3,341 (3.3)	1,727 (8.1)	1,031 (7.5)	1,498 (7.0)	2,194 (9.3)
Guinea	14.4 (0.08) %	7.4 (0.06) %	11.7 (0.09) %	18.8 (0.15) %	749 (3.9)	385 (3.3)	611 (4.7)	982 (1.0)	479 (3.0)	214 (1.7)	357 (3.6)	621 (6.8)
Guinea-Bissau	11.4 (0.08) %	4.4 (0.06) %	8.4 (0.07) %	15.3 (0.21) %	93 (0.7)	36 (0.5)	68 (0.6)	124 (0.1)	59 (0.5)	19 (0.3)	39 (0.5)	76 (1.0)
Kenya	11.7 (0.05) %	9.2 (0.06) %	11.3 (0.05) %	13.7 (0.08) %	2,394 (10.0)	1,880 (12.3)	2,310 (9.6)	2,805 (2.8)	1,454 (6.4)	1,092 (7.7)	1,377 (6.1)	1,725 (9.7)
Liberia	12.7 (0.12) %	5.2 (0.05) %	9.5 (0.09) %	16.9 (0.20) %	261 (2.5)	107 (1.1)	196 (1.8)	348 (0.3)	166 (1.9)	58 (0.6)	112 (1.3)	216 (3.4)
Madagascar	22.3 (0.11) %	11.5 (0.12) %	19.4 (0.16) %	30.3 (0.20) %	2,241 (11.4)	1,154 (12.4)	1,952 (16.6)	3,046 (3.0)	1,626 (10.3)	711 (8.7)	1,301 (12.5)	2,212 (17.7)
Malawi	25.4 (0.17) %	10.2 (0.18) %	20.8 (0.31) %	36.4 (0.25) %	1,991 (13.0)	799 (14.3)	1,629 (24.1)	2,858 (2.9)	1,471 (11.1)	468 (9.7)	1,067 (17.7)	2,105 (16.9)
Mali	13.7 (0.08) %	8.6 (0.07) %	12.2 (0.07) %	17.3 (0.12) %	918 (5.0)	574 (4.9)	813 (4.8)	1,156 (1.2)	592 (3.8)	335 (2.6)	499 (3.6)	751 (6.6)
Mauritania	13.4 (0.09) %	4.6 (0.06) %	9.6 (0.08) %	18.5 (0.19) %	226 (1.5)	78 (1.1)	162 (1.3)	312 (0.3)	153 (1.4)	43 (0.7)	95 (1.0)	205 (2.6)
Mayotte	21.6 (0.21) %	3.9 (0.10) %	12.4 (0.24) %	32.7 (0.57) %	21 (0.2)	4 (0.1)	12 (0.2)	32 (0.0)	16 (0.2)	2 (0.1)	7 (0.2)	22 (0.5)
Mozambique	23.1 (0.09) %	14.7 (0.10) %	21.1 (0.15) %	29.8 (0.20) %	2,631 (9.7)	1,670 (11.8)	2,404 (16.9)	3,394 (3.4)	1,972 (9.0)	1,112 (9.3)	1,703 (15.4)	2,587 (20.6)
Namibia	4.0 (0.05) %	1.8 (0.03) %	2.8 (0.04) %	4.6 (0.07) %	44 (0.6)	20 (0.4)	31 (0.4)	51 (0.1)	26 (0.3)	11 (0.2)	17 (0.2)	29 (0.4)

	Allele frequency (SE)				G6PDd male population (SE)				G6PDd female population (SE)			
**Country**	**Mean**	**Q25%**	**Median**	**Q75%**	**Mean**	**Q25%**	**Median**	**Q75%**	**Mean**	**Q25%**	**Median**	**Q75%**
Niger	7.7 (0.08) %	2.6 (0.05) %	5.3 (0.06) %	10.3 (0.16) %	617 (6.0)	211 (3.7)	426 (4.7)	819 (0.8)	387 (4.7)	111 (2.1)	236 (2.7)	497 (8.5)
Nigeria	17.5 (0.07) %	14.1 (0.08) %	16.9 (0.07) %	20.2 (0.10) %	14,021 (52.3)	11,317 (63.9)	13,515 (54.2)	16,185 (16.2)	8,920 (36.9)	6,898 (44.0)	8,464 (39.2)	10,477 (51.0)
Rwanda	7.7 (0.04) %	3.3 (0.03) %	5.8 (0.06) %	10.1 (0.07) %	389 (2.1)	169 (1.5)	294 (2.8)	509 (0.5)	234 (1.6)	90 (0.6)	163 (1.7)	298 (2.8)
Sao Tome and Principe	15.0 (0.23) %	2.3 (0.05) %	7.4 (0.18) %	20.8 (0.57) %	12 (0.2)	2 (0.0)	6 (0.1)	17 (0.0)	9 (0.2)	1 (0.0)	3 (0.1)	11 (0.3)
Senegal	16.4 (0.05) %	11.3 (0.05) %	15.1 (0.06) %	20.3 (0.10) %	1,046 (3.4)	720 (3.0)	966 (4.1)	1,295 (1.3)	675 (2.7)	424 (1.6)	598 (2.3)	847 (5.2)
Sierra Leone	12.5 (0.13) %	3.4 (0.04) %	7.9 (0.07) %	17.0 (0.17) %	356 (3.6)	98 (1.1)	226 (2.0)	485 (0.5)	247 (3.3)	53 (0.7)	132 (1.6)	313 (4.8)
Somalia	6.1 (0.08) %	1.2 (0.03) %	3.1 (0.07) %	7.7 (0.16) %	282 (3.6)	56 (1.3)	145 (3.4)	356 (0.4)	186 (2.5)	29 (0.7)	80 (2.2)	215 (5.1)
South Africa	5.0 (0.04) %	1.8 (0.04) %	3.3 (0.04) %	6.2 (0.07) %	1,244 (9.4)	438 (9.0)	830 (10.8)	1,563 (1.6)	793 (6.7)	242 (4.9)	482 (5.6)	960 (11.9)
Sudan	15.6 (0.06) %	12.7 (0.06) %	15.3 (0.06) %	18.2 (0.08) %	3,402 (13.6)	2,763 (12.8)	3,322 (13.1)	3,964 (4.0)	2,202 (10.6)	1,691 (9.5)	2,118 (9.2)	2,623 (15.5)
Swaziland	11.6 (0.09) %	4.6 (0.05) %	8.7 (0.08) %	15.5 (0.12) %	68 (0.5)	27 (0.3)	51 (0.5)	91 (0.1)	44 (0.4)	15 (0.2)	29 (0.3)	57 (0.5)
The Gambia	12.6 (0.09) %	8.1 (0.06) %	11.5 (0.08) %	15.9 (0.16) %	109 (0.8)	70 (0.5)	99 (0.7)	138 (0.1)	67 (0.6)	39 (0.3)	58 (0.4)	85 (1.0)
Togo	22.1 (0.08) %	16.7 (0.09) %	21.2 (0.13) %	26.6 (0.12) %	741 (2.7)	560 (3.2)	712 (4.3)	893 (0.9)	499 (2.3)	346 (2.5)	463 (3.0)	616 (3.5)
Uganda	14.8 (0.02) %	12.8 (0.03) %	14.5 (0.03) %	16.5 (0.03) %	2,493 (3.5)	2,162 (5.1)	2,457 (4.8)	2,785 (2.8)	1,504 (2.2)	1,263 (2.8)	1,468 (2.8)	1,706 (3.5)
United Rep of Tanzania	17.9 (0.08) %	11.9 (0.10) %	16.4 (0.09) %	22.3 (0.16) %	4,027 (18.6)	2,671 (21.4)	3,685 (21.0)	5,019 (5.0)	2,716 (15.0)	1,643 (14.4)	2,372 (16.8)	3,431 (33.8)
Zambia	22.9 (0.13) %	14.6 (0.15) %	21.0 (0.11) %	29.4 (0.18) %	1,520 (8.8)	971 (9.7)	1,393 (7.1)	1,950 (2.0)	1,069 (7.6)	606 (6.2)	923 (6.5)	1,393 (10.2)
Zimbabwe	15.9 (0.05) %	11.2 (0.04) %	14.8 (0.07) %	19.4 (0.09) %	988 (3.2)	698 (2.4)	924 (4.3)	1,212 (1.2)	650 (2.6)	421 (1.5)	586 (3.6)	808 (4.3)
**American MECs**												
Argentina	1.3 (0.03) %	0.5 (0.01) %	0.9 (0.02) %	1.6 (0.04) %	261 (5.1)	98 (2.1)	169 (3.5)	313 (0.3)	159 (3.3)	51 (1.1)	92 (2.1)	181 (5.1)
Belize	4.1 (0.04) %	0.9 (0.01) %	2.2 (0.03) %	5.1 (0.07) %	6 (0.1)	1 (0.0)	3 (0.1)	8 (0.0)	4 (0.1)	1 (0.0)	2 (0.0)	4 (0.1)
Bolivia	1.0 (0.02) %	0.1 (0.00) %	0.2 (0.01) %	0.8 (0.02) %	51 (1.1)	3 (0.1)	11 (0.3)	41 (0.0)	30 (0.8)	2 (0.0)	6 (0.2)	21 (0.6)
Brazil	5.4 (0.04) %	3.6 (0.02) %	4.8 (0.04) %	6.5 (0.06) %	5,153 (38.8)	3,501 (21.3)	4,647 (40.4)	6,213 (6.2)	3,203 (28.3)	1,994 (16.6)	2,758 (25.1)	3,897 (48.0)
Colombia	5.8 (0.04) %	3.4 (0.02) %	4.9 (0.04) %	7.3 (0.06) %	1,311 (8.3)	764 (5.4)	1,118 (9.4)	1,667 (1.7)	798 (6.3)	419 (2.3)	638 (5.1)	1,008 (11.5)
Costa Rica	0.8 (0.01) %	0.2 (0.00) %	0.4 (0.00) %	1.0 (0.01) %	20 (0.2)	4 (0.1)	9 (0.1)	23 (0.0)	10 (0.1)	2 (0.0)	4 (0.0)	11 (0.1)
Dominican Republic	8.6 (0.14) %	0.9 (0.03) %	3.0 (0.08) %	10.0 (0.31) %	443 (7.1)	44 (1.5)	154 (4.2)	511 (0.5)	296 (5.3)	22 (0.7)	79 (2.3)	288 (10.2)
Ecuador	6.0 (0.03) %	2.4 (0.03) %	4.2 (0.05) %	7.5 (0.06) %	411 (2.1)	166 (2.1)	292 (3.2)	519 (0.5)	240 (1.5)	87 (1.1)	157 (1.9)	294 (2.0)
El Salvador	3.8 (0.01) %	2.4 (0.02) %	3.3 (0.02) %	4.8 (0.01) %	112 (0.3)	69 (0.5)	98 (0.6)	140 (0.1)	65 (0.2)	39 (0.3)	56 (0.3)	81 (0.2)
French Guiana	1.4 (0.02) %	0.3 (0.01) %	0.7 (0.01) %	1.6 (0.02) %	2 (0.0)	0 (0.0)	1 (0.0)	2 (0.0)	1 (0.0)	0 (0.0)	0 (0.0)	1 (0.0)
Guatemala	4.0 (0.04) %	1.5 (0.02) %	2.7 (0.03) %	5.1 (0.07) %	280 (2.8)	103 (1.1)	189 (2.4)	355 (0.4)	163 (1.9)	54 (0.6)	102 (1.4)	199 (2.8)
Guyana	5.3 (0.07) %	1.4 (0.02) %	3.0 (0.06) %	6.4 (0.12) %	20 (0.3)	5 (0.1)	11 (0.2)	25 (0.0)	12 (0.2)	3 (0.1)	6 (0.1)	13 (0.3)
Haiti	10.3 (0.14) %	1.9 (0.05) %	5.2 (0.12) %	13.2 (0.24) %	522 (6.9)	94 (2.4)	261 (6.1)	665 (0.7)	349 (5.5)	48 (1.2)	141 (3.4)	395 (9.6)
Honduras	4.6 (0.03) %	1.5 (0.02) %	2.9 (0.02) %	5.8 (0.07) %	176 (1.2)	55 (0.9)	111 (0.8)	219 (0.2)	101 (0.8)	28 (0.5)	58 (0.5)	118 (1.7)
Mexico	1.1 (0.01) %	0.8 (0.01) %	1.0 (0.01) %	1.3 (0.01) %	619 (5.7)	430 (5.0)	555 (6.1)	733 (0.7)	327 (3.2)	222 (2.5)	291 (3.3)	387 (4.4)
Nicaragua	3.0 (0.06) %	0.6 (0.01) %	1.5 (0.02) %	3.6 (0.07) %	88 (1.7)	18 (0.3)	43 (0.7)	103 (0.1)	51 (1.1)	9 (0.2)	22 (0.3)	56 (1.2)

Country	Allele frequency (SE)				G6PDd male population (SE)				G6PDd female population (SE)			
	Mean	Q25%	Median	Q75%	Mean	Q25%	Median	Q75%	Mean	Q25%	Median	Q75%
Panama	2.3 (0.05) %	0.4 (0.01) %	0.9 (0.02) %	2.5 (0.07) %	41 (0.9)	6 (0.1)	16 (0.3)	44 (0.0)	23 (0.6)	3 (0.1)	8 (0.2)	22 (0.7)
Paraguay	7.5 (0.09) %	1.1 (0.03) %	3.2 (0.06) %	8.8 (0.21) %	244 (2.8)	34 (0.8)	105 (1.9)	288 (0.3)	157 (2.2)	17 (0.4)	54 (0.9)	163 (3.5)
Peru	0.5 (0.01) %	0.1 (0.00) %	0.2 (0.00) %	0.6 (0.01) %	79 (1.2)	13 (0.3)	33 (0.5)	84 (0.1)	43 (0.7)	6 (0.1)	17 (0.4)	43 (0.8)
Suriname	1.1 (0.01) %	0.4 (0.01) %	0.7 (0.01) %	1.3 (0.02) %	3 (0.0)	1 (0.0)	2 (0.0)	3 (0.0)	1 (0.0)	1 (0.0)	1 (0.0)	2 (0.0)
Venezuela	13.3 (0.12) %	4.0 (0.06) %	8.6 (0.09) %	18.0 (0.32) %	1,936 (17.8)	583 (9.4)	1,251 (13.2)	2,617 (2.6)	1,325 (15.0)	316 (5.8)	732 (10.4)	1,701 (35.4)
**Eurasian MECs**												
Afghanistan	8.0 (0.06) %	5.6 (0.05) %	7.4 (0.05) %	9.8 (0.08) %	1,209 (9.5)	845 (7.2)	1,115 (8.2)	1,470 (1.5)	681 (6.0)	436 (3.6)	599 (5.2)	833 (7.8)
Argentina	1.3 (0.03) %	0.5 (0.01) %	0.9 (0.02) %	1.6 (0.04) %	261 (5.1)	98 (2.1)	169 (3.5)	313 (0.3)	159 (3.3)	51 (1.1)	92 (2.1)	181 (5.1)
Azerbaijan	10.4 (0.08) %	8.9 (0.07) %	10.2 (0.09) %	11.7 (0.09) %	461 (3.6)	393 (3.2)	452 (4.0)	518 (0.5)	275 (2.4)	228 (2.2)	267 (2.6)	314 (2.5)
Bangladesh	4.7 (0.03) %	2.4 (0.01) %	3.8 (0.02) %	5.9 (0.04) %	3,898 (25.1)	2,002 (12.1)	3,168 (20.3)	4,942 (4.9)	2,110 (15.5)	1,007 (7.0)	1,624 (12.3)	2,636 (19.4)
Bhutan	7.4 (0.05) %	3.6 (0.04) %	5.9 (0.06) %	9.6 (0.11) %	28 (0.2)	14 (0.1)	23 (0.2)	37 (0.0)	15 (0.1)	6 (0.1)	11 (0.1)	18 (0.2)
Cambodia	14.8 (0.03) %	11.8 (0.05) %	14.3 (0.04) %	17.2 (0.05) %	1,089 (2.1)	871 (3.4)	1,055 (3.3)	1,268 (1.3)	685 (1.6)	522 (2.2)	653 (1.7)	811 (3.2)
China	5.7 (0.04) %	3.5 (0.04) %	4.7 (0.05) %	6.8 (0.05) %	41,186 (314.1)	25,014 (273.3)	33,675 (350.2)	48,717 (48.7)	23,765 (192.0)	13,427 (164.0)	18,555 (204.4)	27,859 (222.7)
Dem People's Rep Korea	0.7 (0.02) %	0.0 (0.00) %	0.1 (0.00) %	0.4 (0.01) %	78 (2.8)	2 (0.0)	10 (0.2)	42 (0.0)	46 (2.0)	1 (0.0)	5 (0.1)	22 (0.4)
Georgia	1.3 (0.01) %	0.7 (0.01) %	1.1 (0.02) %	1.7 (0.02) %	26 (0.3)	14 (0.2)	21 (0.3)	33 (0.0)	15 (0.2)	8 (0.1)	12 (0.2)	19 (0.2)
India	8.2 (0.03) %	6.9 (0.03) %	8.0 (0.03) %	9.3 (0.04) %	51,368 (204.6)	43,246 (188.0)	50,009 (218.2)	57,985 (58.0)	28,756 (132.1)	23,452 (122.3)	27,708 (121.3)	32,947 (175.5)
Indonesia	7.7 (0.05) %	5.3 (0.05) %	7.1 (0.05) %	9.4 (0.06) %	8,948 (53.9)	6,180 (55.8)	8,204 (57.0)	10,901 (10.9)	5,494 (37.8)	3,484 (30.6)	4,856 (40.1)	6,756 (51.7)
Iran (Islamic Rep of)	12.3 (0.05) %	9.9 (0.04) %	11.8 (0.05) %	14.1 (0.06) %	4,672 (20.8)	3,788 (15.7)	4,510 (20.7)	5,356 (5.4)	2,803 (13.8)	2,186 (11.9)	2,661 (13.0)	3,256 (13.8)
Iraq	11.2 (0.03) %	8.1 (0.03) %	10.6 (0.04) %	13.5 (0.05) %	1,762 (5.1)	1,279 (5.3)	1,669 (6.0)	2,130 (2.1)	1,056 (3.5)	720 (4.0)	970 (4.1)	1,297 (6.1)
Korea, Rep of	0.8 (0.02) %	0.1 (0.00) %	0.2 (0.00) %	0.6 (0.01) %	182 (4.7)	17 (0.4)	50 (1.1)	145 (0.1)	100 (2.8)	8 (0.2)	24 (0.5)	72 (1.4)
Kyrgyzstan	1.6 (0.04) %	0.1 (0.00) %	0.3 (0.01) %	1.2 (0.04) %	43 (1.1)	3 (0.1)	9 (0.2)	33 (0.0)	26 (0.9)	1 (0.0)	5 (0.1)	17 (0.6)
Lao People's Dem Rep	16.6 (0.08) %	11.6 (0.09) %	15.6 (0.11) %	20.5 (0.11) %	533 (2.5)	372 (2.9)	500 (3.6)	657 (0.7)	350 (1.9)	224 (2.1)	315 (2.9)	437 (2.8)
Malaysia	8.3 (0.04) %	6.6 (0.04) %	8.0 (0.05) %	9.6 (0.06) %	1,177 (5.9)	942 (5.2)	1,129 (6.5)	1,359 (1.4)	669 (3.7)	506 (3.2)	627 (4.0)	782 (5.0)
Myanmar	7.4 (0.03) %	4.1 (0.03) %	6.1 (0.03) %	9.3 (0.04) %	1,833 (7.8)	1,028 (6.2)	1,523 (7.3)	2,309 (2.3)	1,115 (6.0)	577 (3.9)	880 (5.9)	1,384 (5.6)
Nepal	7.2 (0.06) %	2.9 (0.02) %	5.3 (0.03) %	9.4 (0.11) %	1,070 (8.8)	436 (3.4)	786 (4.2)	1,390 (1.4)	648 (6.5)	230 (2.0)	434 (3.2)	814 (10.2)
Pakistan	16.2 (0.09) %	10.8 (0.07) %	15.0 (0.11) %	20.4 (0.16) %	15,626 (90.9)	10,393 (63.4)	14,495 (108.4)	19,648 (19.6)	10,150 (75.4)	5,984 (50.3)	8,900 (71.1)	13,089 (130.8)
Papua New Guinea	7.9 (0.04) %	6.0 (0.03) %	7.4 (0.04) %	9.3 (0.05) %	277 (1.5)	212 (1.0)	261 (1.5)	325 (0.3)	157 (1.0)	114 (0.6)	143 (1.0)	185 (1.0)
Philippines	2.5 (0.01) %	2.4 (0.01) %	2.5 (0.01) %	2.5 (0.01) %	1,153 (6.1)	1,117 (5.9)	1,151 (6.0)	1,187 (1.2)	581 (3.2)	556 (3.0)	580 (3.2)	604 (3.6)
Saudi Arabia	12.9 (0.06) %	10.4 (0.08) %	12.4 (0.07) %	14.9 (0.05) %	1,877 (8.1)	1,511 (11.7)	1,794 (9.7)	2,161 (2.2)	957 (4.5)	741 (6.6)	899 (5.8)	1,113 (4.7)
Solomon Islands	24.0 (0.11) %	15.7 (0.11) %	22.3 (0.13) %	30.9 (0.17) %	66 (0.3)	43 (0.3)	62 (0.4)	86 (0.1)	43 (0.2)	25 (0.2)	38 (0.2)	56 (0.4)
Sri Lanka	3.0 (0.03) %	2.6 (0.02) %	2.9 (0.03) %	3.3 (0.03) %	299 (2.7)	258 (2.5)	291 (2.7)	331 (0.3)	168 (1.7)	140 (1.3)	161 (1.7)	188 (1.7)
Tajikistan	1.7 (0.03) %	0.4 (0.01) %	0.8 (0.02) %	1.9 (0.03) %	59 (1.0)	12 (0.2)	29 (0.6)	66 (0.1)	33 (0.7)	6 (0.1)	15 (0.3)	34 (0.5)
Thailand	13.8 (0.06) %	11.9 (0.06) %	13.6 (0.06) %	15.5 (0.05) %	4,636 (19.0)	3,981 (18.6)	4,544 (20.0)	5,188 (5.2)	2,923 (13.4)	2,428 (13.6)	2,830 (14.8)	3,312 (12.6)

Country	Allele frequency (SE)				G6PDd male population (SE)				G6PDd female population (SE)			
	Mean	Q25%	Median	Q75%	Mean	Q25%	Median	Q75%	Mean	Q25%	Median	Q75%
Timor-Leste	7.5 (0.07) %	2.5 (0.03) %	5.0 (0.05) %	9.7 (0.11) %	44 (0.4)	15 (0.2)	29 (0.3)	58 (0.1)	25 (0.3)	7 (0.1)	15 (0.2)	31 (0.5)
Turkey	4.1 (0.04) %	3.0 (0.03) %	3.8 (0.04) %	4.9 (0.05) %	1,551 (14.8)	1,128 (12.1)	1,437 (14.8)	1,850 (1.9)	863 (8.8)	601 (6.9)	783 (8.3)	1,034 (12.8)
Uzbekistan	2.2 (0.04) %	0.4 (0.01) %	1.0 (0.01) %	2.4 (0.04) %	298 (5.4)	53 (0.9)	132 (1.4)	330 (0.3)	176 (3.8)	27 (0.5)	68 (0.9)	179 (3.6)
Vanuatu	8.2 (0.03) %	6.9 (0.02) %	8.0 (0.03) %	9.3 (0.04) %	10 (0.0)	9 (0.0)	10 (0.0)	12 (0.0)	6 (0.0)	4 (0.0)	5 (0.0)	6 (0.0)
Viet Nam	10.8 (0.11) %	6.0 (0.06) %	8.9 (0.10) %	13.9 (0.15) %	4,745 (46.5)	2,625 (24.5)	3,902 (43.1)	6,102 (6.1)	3,098 (36.6)	1,488 (15.5)	2,310 (33.0)	3,922 (54.1)
Yemen	8.4 (0.08) %	1.9 (0.04) %	4.6 (0.06) %	10.9 (0.15) %	1,031 (9.9)	238 (5.2)	565 (7.0)	1,329 (1.3)	656 (7.2)	121 (2.8)	308 (4.7)	781 (11.9)
