# Supplementary material for: G6PD Deficiency Prevalence and Estimates of Affected Populations in Malaria Endemic Countries: A Geostatistical Model-Based Map
Source: PLoS Med. 2012 Nov 13;9(11):e1001339. doi: 10.1371/journal.pmed.1001339 (PMC3496665; doi:10.1371/journal.pmed.1001339)
Supplement: Table S3 — Reported observations of class II and III G6PD variants from malaria endemic countries. (PDF) [file pmed.1001339.s010.pdf]

Table S3. Reported observations of Class II and III G6PD variants from malaria endemic countries. Only MECs for which data were available are listed (n = 54 of 90 MECs).

The A- variant includes mostly variants carrying the 202 G→A and 376 A→G mutations; but also some diagnoses determined only from the 202 locus (in cases where only the 376A→G mutation was identified (G6PD A), the record was not included here as that mutation is from Class IV), as well as the 680 G→T/376 A→G and the 968 T→C/376 A→G variants.

Country	Number of occurrences	Class II variants	Class III variants
**African MECs**			
Angola	1		A- [1]
Benin	1		A- [2]
Burkina Faso	4		A- [3-5]
Cameroon	8		A- [6,7]
Cape Verde	1		A- [8]
Central African Republic	1		A- [9]
Comores	2	Mediterranean [10]	A- [10]
Congo	1		A- [11]
Côte d'Ivoire	4		A- [7,12,13]
DR Congo	1		A- [14]
Gabon	3		A- [7,15,16]
Ghana	7		A- [5,16-20]
Guinea	2		A- [21]
Kenya	6		A- [5,22-24]
Malawi	4		A- [25-27]
Mali	6		A- [5,7,28-30]
Mauritania	1		A- [7]
Mozambique	2		A- [31]
Namibia	6		A- [32,33]
Nigeria	21		A- [5,14,16,23,34-43]<br>Ilesha [44]
Rwanda	1		A- [45]
Sao Tome and Principe	1		A- [46]
Senegal	7	Santamaria [47]	A- [7,47-51]
Sierra Leone	2		A- [52]
South Africa	2	Mediterranean [53]	A- [14,33]
Sudan	7	Mediterranean [54]	A- [54-59]
The Gambia	8	Santamaria [60]	A- [14,60-63]
Uganda	3		A- [64-66]
United Republic of Tanzania	6		A- [5,67-70]
**American MECs**			
Brazil	39	Amazonia [71]<br>Ananindeau [71]<br>Belem [71]<br>Crispim [71]<br>Chatham [72]<br>Farroupilha [73]	A- [71-74,76-84]<br>Bahia [78]<br>Lages [73]<br>Seattle [71,73,75,81]<br>Seattle-like [76,85]

		Mediterranean [72-76]<br>Santamaria [71]	
Costa Rica	3	Santamaria [86,87]	A- [86]
Ecuador	2		A- [88]
Guyana	1		A- [7]
Mexico	24	Santamaria [89]<br>Union [89]<br>Valladolid [90]<br>Vanua Lava [89]<br>Viangchan-Jammu [90]	A- [89,91-94]<br>Mexico City [95]<br>Seattle [89,94]
Panama	3	Mediterranean [96]	A- [96]
**Eurasian MECs**			
Cambodia	11	Canton [97]<br>Kaiping [98]<br>Valladolid [97]<br>Viangchan-Jammu [97-99]	A- [100]<br>Mahidol [97,101]
China	266	Canton [102-136]<br>Chinese-1 [103,124,137]<br>Coimbra [106,109,130,136-138]<br>Fushan [109,121,139,140]<br>Haikou [103]<br>Hechi [109]<br>Kaiping [103-110,112,113,115-119,121-127,129,132-136,138-144]<br>Liuzhou [109]<br>Miaoli [109,113,126,129,137]<br>Nankang [103,124,126,145]<br>Songklanagarind [109]<br>Taipei [113,126,133,135,136]<br>Taipei-Hakka [111]<br>Union [106,124,126,127,132-134,136,146]<br>Valladolid [122,124]<br>Viangchan-Jammu [103,106,109,112,118,121-125,127,133,134]<br>Gaohe/Kaiping [106,124]	A- [109,117]<br>Chinese-5 [104-106,109,113,118,122-127,129,132-136]<br>Gaohe [103-106,108-110,112,113,115,117,118,121-124,126,127,129,131-136,143,144]<br>Guangzhou [106,117]<br>Keelung [137]<br>Mahidol [106,113,122,124,126,127,129,133,135,136,147]<br>Mahidol-like [117]<br>Nanning [109]<br>Quing Yan [103,104,106,109,113,117,122-124,126,127,129,133-136,148]<br>Ube Konan [144]
India	66	Chatham [149]<br>Coimbra [150-153]<br>Mediterranean [149,152-156]<br>Namouru [150,151,153]<br>Nilgiri [150,151]	Kalyan-Kerala [149-153,156-159];<br>Orissa [149,152-154,156,160,161]
Indonesia	76	Canton [162-167]<br>Chatham [162,165,166,168-171]<br>Coimbra [162,168,169,171]<br>Kaiping [162,164-166,168-172]<br>Mediterranean [163,171]<br>Surabaya [162]<br>Union [165,166]<br>Vanua Lava [162,165,166,168-171,173]<br>Viangchan-Jammu [165-169,171,174]	Bajo Maumere [168]<br>Chinese-5 [168]<br>Gaohe [162]<br>Mahidol [163]
Iran	29	Canton [175,176]<br>Chatham [175-182]<br>Cosenza [177,178,181,182]	A- [175,176]

		Mediterranean [155,175-186]	
Iraq	11	Chatham [187,188]<br>Mediterranean [187-191]	A- [188,189]
Lao PDR	1	Viangchan-Jammu [162]	
Malaysia	61	Andalus [192]<br>Canton [192-200]<br>Chatham [192,196,199]<br>Coimbra [192,198-200]<br>Kaiping [192,194,195,197-200]<br>Mediterranean [192,198-200]<br>Namouru [198]<br>Nankang [196]<br>Union [192,196]<br>Vanua Lava [192,199]<br>Viangchan-Jammu [192,194-196,198-200]	Chinese-5 [194-197]<br>Gaohe [194-198,200]<br>Mahidol [192,196,198-200]<br>Orissa [192,199]<br>Quing Yan [195,196]
Myanmar	27	Canton [162,201]<br>Coimbra [201,202]<br>Viangchan-Jammu [202]<br>Kaiping [202]<br>Mediterranean [202]<br>Union [162,201]<br>Valladolid [202]	Kerala-Kalyan [202]<br>Mahidol [162,201-203]`
Nepal	1	Mediterranean [204]	
Pakistan	9	Chatham [205]<br>Mediterranean [155,205-208]	Orissa [205]
Papua New Guinea	16	Viangchan-Jammu [209]<br>Kaiping [210]<br>Mediterranean-like [211,212]<br>Union [210]<br>Union-like [211,212]<br>Vanua Lava [209]	
Philippines	2	Union [213,214]	
Saudi Arabia	81	Aures [215-218]<br>Chatham [216-218]<br>Kaiping [218]<br>Mediterranean [191,215-230]<br>Mediterranean-like [221-226]<br>S. Antioco [216]<br>Union [216]<br>Viangchan [216]	A- [216,218,220-224,226-228,231]<br>Kerala-Kalyan [218]<br>Sibari [230]
Solomon Islands	1	Union [232]	
Thailand	45	Canton [233-237]<br>Kaiping [233-239]<br>Mediterranean [233]<br>Songklanagarind [233]<br>Union [233-237]<br>Vanua Lava [240]<br>Viangchan-Jammu [233,234,237-239]	Chinese-5 [234]<br>Gaohe [233,237,238]<br>Kerala-Kalyan [238]<br>Mahidol [233-239,241,242]<br>Quing Yan [233]
Turkey	7	Chatham [243,244]<br>Mediterranean [243,245-247]	A- [243]
Vanuatu	4	Namouru [248]<br>Naone [248]<br>Union [248]<br>Vanua Lava [248]	

Viet Nam	19	Bao Loc [249]<br>Canton [249-251]<br>Coimbra [252]<br>Kaiping [249]<br>Union [249]<br>Viangchan-Jammu [249,252]	Chinese-5 [252]<br>Gaohe [249,252]<br>Mahidol [250]<br>Quing Yan [249]

References

1. Nurse GT, Jenkins T, David JH, Steinberg AG (1979) The Njinga of Angola: a serogenetic study. Annals of Human Biology 6: 337-348.
2. Biondi G, Rickards O, Martinez-Labarga C, Taraborelli T, Ciminelli B, et al. (1996) Biodemography and genetics of the Berba of Benin. Am J Phys Anthropol 99: 519-535.
3. Meissner PE, Coulibaly B, Mandi G, Mansmann U, Witte S, et al. (2005) Diagnosis of red cell G6PD deficiency in rural Burkina Faso: comparison of a rapid fluorescent enzyme test on filter paper with polymerase chain reaction based genotyping. Br J Haematol 131: 395-399.
4. Modiano D, Luoni G, Sirima BS, Lanfrancotti A, Petrarca V, et al. (2001) The lower susceptibility to *Plasmodium falciparum* malaria of Fulani of Burkina Faso (West Africa) is associated with low frequencies of classic malaria-resistance genes. Trans R Soc Trop Med Hyg 95: 149-152.
5. Carter N, Pamba A, Duparc S, Waitumbi JN (2011) Frequency of glucose-6-phosphate dehydrogenase deficiency in malaria patients from six African countries enrolled in two randomized anti-malarial clinical trials. Malar J 10: 241.
6. Bernstein SC, Bowman JE, Kaptue Noche L (1980) Population studies in Cameroon: hemoglobin S, glucose-6-phosphate dehydrogenase deficiency and falciparum malaria. Hum Hered 30: 251-258.
7. Kahn A, Boivin P, Lagneau J (1973) [Phenotypes of erythrocytic glucose-6-phosphate dehydrogenase in black people. Examination of 301 black people living in France and description of 9 different variants. High incidence of deficiency of an enzyme of "B" mobility]. Humangenetik 18: 261-270.
8. Alves J, Machado P, Silva J, Goncalves N, Ribeiro L, et al. (2010) Analysis of malaria associated genetic traits in Cabo Verde, a melting pot of European and sub Saharan settlers. Blood Cells Mol Dis 44: 62-68.
9. Vergnes H, Sevin A, Sevin J, Jaeger G (1979) Population genetic studies of the Aka pygmies (Central Africa): a survey of red cell and serum enzymes. Hum Genet 48: 343-355.
10. Badens C, Martinez di Montemuros F, Thuret I, Michel G, Mattei JF, et al. (2000) Molecular basis of haemoglobinopathies and G6PD deficiency in the Comorian population. Hematol J 1: 264-268.
11. Bouanga JC, Mouele R, Prehu C, Wajcman H, Feingold J, et al. (1998) Glucose-6-phosphate dehydrogenase deficiency and homozygous sickle cell disease in Congo. Hum Hered 48: 192-197.

12. Coulibaly FH, Koffi G, Toure HA, Bouanga JC, Allangba O, et al. (2000) Molecular genetics of glucose-6-phosphate dehydrogenase deficiency in a population of newborns from Ivory Coast. Clin Biochem 33: 411-413.
13. Vergnes H, Cabannes R (1976) Polymorphism of erythrocyte and serum enzyme systems in the Gagu of the Ivory Coast. Ann Hum Biol 3: 423-429.
14. Saunders MA, Hammer MF, Nachman MW (2002) Nucleotide variability at G6pd and the signature of malarial selection in humans. Genetics 162: 1849-1861.
15. Mombo LE, Ntoumi F, Bisseye C, Ossari S, Lu CY, et al. (2003) Human genetic polymorphisms and asymptomatic Plasmodium falciparum malaria in Gabonese schoolchildren. Am J Trop Med Hyg 68: 186-190.
16. May J, Meyer CG (2003) A synonymous mutation of ancient origin in the glucose-6-phosphate dehydrogenase gene and assessment of haplotypes. Blood Cells Mol Dis 30: 144-145.
17. Acquaye CT, Oldham JH (1974) A preliminary report on survey and characterization of haemoglobins and glucose-6-phosphate dehydrogenase variants in Ghana. West Afr J Pharmacol Drug Res 1: 25P-28P.
18. Owusu SK, Opare-Mante A (1972) Electrophoretic characterisation of glucose-6-phosphate dehydrogenase in Ghana. Lancet 2: 44.
19. Timmann C, Evans JA, Konig IR, Kleensang A, Ruschendorf F, et al. (2007) Genome-wide linkage analysis of malaria infection intensity and mild disease. PLoS Genet 3: e48.
20. Armoo S, Wilson M, Boakye D, Quakyi I (2010) Studies on Abo Blood Groups, Haemoglobinopathies and G6pd Genotypes, and Plasmodium Falciparum Infection in Kpone-on-Sea, Ghana. American Journal of Tropical Medicine and Hygiene 83: 82-82.
21. Millimono TS, Loua KM, Rath SL, Relvas L, Bento C, et al. (2012) High prevalence of hemoglobin disorders and glucose-6-phosphate dehydrogenase (G6PD) deficiency in the Republic of Guinea (West Africa). Hemoglobin 36: 25-37.
22. Othieno-Obel A (1972) East African variant of glucose-6-phosphate dehydrogenase. East Afr Med J 49: 230-234.
23. Vulliamy TJ, Othman A, Town M, Nathwani A, Falusi Y, et al. (1991) Linkage disequilibrium of polymorphic sites in the G6PD gene in African populations and the origin of G6PD A. Gene Geogr 5: 13-21.
24. Shah SS, Macharia A, Uyoga S, Williams TN (2011) Modeling the relationship between genotype and biochemotype at the *G6PD* locus in Kenya. pp. Unpublished work.
25. Nkhoma ET, Mu JB, Krause MA, Diakite SA, Kalilani L, et al. (2009) Effect of the a-Form of G6pd Deficiency on Maternal Plasmodium Falciparum Parasitemia and Pregnancy Outcomes. American Journal of Tropical Medicine and Hygiene 81: 86-86.
26. Calis JC, Phiri KS, Faragher EB, Brabin BJ, Bates I, et al. (2008) Severe anemia in Malawian children. New England Journal of Medicine 358: 888-899.
27. Senga EL, Harper G, Koshy G, Kazembe PN, Brabin BJ (2011) Reduced risk for placental malaria in iron deficient women. Malaria journal 10: 47.
28. Duflo B, Diallo A, Toure K, Soula G (1979) [Glucose-6-phosphate dehydrogenase deficiency in Mali. Epidemiology and pathological aspects]. Bull Soc Pathol Exot Filiales 72: 258-264.
29. Junien C, Chaventre A, Fofana Y, Lapoumeroulie C, Floury B, et al. (1982) Glucose-6-phosphate dehydrogenase and hemoglobin variants in Kel Kummer Tuareg and related groups. Indirect evidence for alpha-thalassemia trait. Hum Hered 32: 318-328.
30. Crompton PD, Traore B, Kayentao K, Doumbo S, Ongoiba A, et al. (2008) Sickle cell trait is associated with a delayed onset of malaria: Implications for time-to-event analysis in clinical studies of malaria. Journal of Infectious Diseases 198: 1265-1275.
31. Reys L, Manso C, Stamatoyannopoulos G (1970) Genetic studies on southeastern Bantu of Mozambique. I. Variants of glucose-6-phosphate dehydrogenase. Am J Hum Genet 22: 203-215.

32. Nurse GT, Jenkins T (1977) Serogenetic studies on the Kavango peoples of South West Africa. Annals of Human Biology 4: 465-478.
33. Coetzee MJ, Bartleet SC, Ramsay M, Jenkins T (1992) Glucose-6-phosphate dehydrogenase (G6PD) electrophoretic variants and the PvuII polymorphism in southern African populations. Hum Genet 89: 111-113.
34. Ademowo OG, Falusi AG (2002) Molecular epidemiology and activity of erythrocyte G6PD variants in a homogeneous Nigerian population. East Afr Med J 79: 42-44.
35. Bienzle U, Guggenmoos-Holzmann I, Luzzatto L (1979) Malaria and erythrocyte glucose-6-phosphate dehydrogenase variants in West Africa. Am J Trop Med Hyg 28: 619-621.
36. Guggenmoos-Holzmann I, Bienzle U, Luzzatto L (1981) Plasmodium falciparum malaria and human red cells. II. Red cell genetic traits and resistance against malaria. Int J Epidemiol 10: 16-22.
37. Luzzatto L, Allan NC (1968) Relationship between the genes for glucose-6-phosphate dehydrogenase and for haemoglobin in a Nigerian population. Nature 219: 1041-1042.
38. Porter IH, Boyer SH, Watson-Williams EJ, Adam A, Szeinberg A, et al. (1964) Variation of Glucose-6-Phosphate Dehydrogenase in Different Populations. Lancet 283: 895-899.
39. Mockenhaupt FP, May J, Stark K, Falusi AG, Meyer CG, et al. (1999) Serum transferrin receptor levels are increased in asymptomatic and mild *Plasmodium falciparum*-infection. Haematologica 84: 869-873.
40. Bienzle U, Effiong CE, Aimaku VE, Luzzatto L (1976) Erythrocyte enzymes in neonatal jaundice. Acta Haematologica 55: 10-20.
41. Bienzle U, Ayeni O, Lucas AO, Luzzatto L (1972) Glucose-6-phosphate dehydrogenase and malaria. Greater resistance of females heterozygous for enzyme deficiency and of males with non-deficient variant. Lancet 1: 107-110.
42. May J, Meyer CG, Grossterlinden L, Ademowo OG, Mockenhaupt FP, et al. (2000) Red cell glucose-6-phosphate dehydrogenase status and pyruvate kinase activity in a Nigerian population. Trop Med Int Health 5: 119-123.
43. Porter IH, Boyer SH, Watson-Williams EJ, Adam A, Szeinberg A, et al. (1964) Variation of Glucose-6-Phosphate Dehydrogenase in Different Populations. Lancet 1: 895-899.
44. Vulliamy TJ, D'Urso M, Battistuzzi G, Estrada M, Foulkes NS, et al. (1988) Diverse point mutations in the human glucose-6-phosphate dehydrogenase gene cause enzyme deficiency and mild or severe hemolytic anemia. Proceedings of the National Academy of Sciences of the United States of America 85: 5171-5175.
45. Gahutu JB, Musemakweri A, Harms G, Mockenhaupt FP (2012) Prevalence of classic erythrocyte polymorphisms among 749 children in southern highland Rwanda. Transactions of the Royal Society of Tropical Medicine and Hygiene 106: 63-65.
46. Manco L, Botigue LR, Ribeiro ML, Abade A (2007) G6PD deficient alleles and haplotype analysis of human G6PD locus in Sao Tome e Principe (West Africa). Hum Biol 79: 679-686.
47. De Araujo C, Migot-Nabias F, Guitard J, Pelleau S, Vulliamy T, et al. (2006) The role of the G6PD AEth376G/968C allele in glucose-6-phosphate dehydrogenase deficiency in the seerer population of Senegal. Haematologica 91: 262-263.
48. Bouloux C, Gomila J, Langaney A (1972) Hemotypology of the Bedik. Hum Biol 44: 289-302.
49. Sarr JB, Pelleau S, Toly C, Guitard J, Konate L, et al. (2006) Impact of red blood cell polymorphisms on the antibody response to *Plasmodium falciparum* in Senegal. Microbes and Infection 8: 1260-1268.
50. Vergnes H, Gherardi M, Bouloux C (1975) Erythrocyte glucose-6-phosphate dehydrogenase in the Niokolonko (Malinke of the Niokolo) of Eastern Senegal. Identification of a slow variant with normal activity (Tacoma-like). Hum Hered 25: 80-87.
51. Courtin D, Milet J, Bertin G, Vafa M, Sarr JB, et al. (2011) G6PD A-variant influences the antibody responses to Plasmodium falciparum MSP2. Infection, genetics and evolution : journal of molecular epidemiology and evolutionary genetics in infectious diseases 11: 1287-1292.

52. Jalloh A, Jalloh M, Gamanga I, Baion D, Sahr F, et al. (2008) G6PD deficiency assessment in Freetown, Sierra Leone, reveals further insight into the molecular heterogeneity of G6PD A. J Hum Genet 53: 675-679.
53. Lane AB, Grant SC, Jenkins T (1983) G6pd Witwatersrand, a New Variant Found in a South-African Ashkenazi Jew and the Report of a 2nd Case of G6pd Panama. South African Journal of Science 79: 414-416.
54. Saha N, Samuel AP (1991) Characterization of glucose-6-phosphate dehydrogenase variants in the Sudan--including GdKhartoum, a hyperactive slow variant. Hum Hered 41: 17-21.
55. Bayoumi RA, Saha N (1987) Some blood genetic markers of the Nuba and Hawazma tribes of western Sudan. Am J Phys Anthropol 73: 379-388.
56. Saha N, el Seikh FS (1987) Some blood genetic characteristics of several Sudanese tribes. Am J Phys Anthropol 73: 397-406.
57. Saha N, Samuel AP, Omer A, Ahmed MA, Hussein AA, et al. (1978) A study of some genetic characteristics of the population of the Sudan. Ann Hum Biol 5: 569-575.
58. Saha N, Samuel AP, Omer A, Hoffbrand AV (1983) The inter- and intra-tribal distribution of red cell G6PD phenotypes in Sudan. Hum Hered 33: 39-43.
59. Samuel AP, Saha N, Omer A, Hoffbrand AV (1981) Quantitative expression of G6PD activity of different phenotypes of G6PD and haemoglobin in a Sudanese population. Hum Hered 31: 110-115.
60. Clark TG, Fry AE, Auburn S, Campino S, Diakite M, et al. (2009) Allelic heterogeneity of G6PD deficiency in West Africa and severe malaria susceptibility. Eur J Hum Genet 17: 1080-1085.
61. Atkinson SH, Rockett K, Sirugo G, Bejon PA, Fulford A, et al. (2006) Seasonal childhood anaemia in West Africa is associated with the haptoglobin 2-2 genotype. PLoS Med 3: e172.
62. Sirugo G, Schaefer EA, Mendy A, West B, Bailey R, et al. (2004) Is G6PD A- deficiency associated with recurrent stillbirths in The Gambia? Am J Med Genet A 128A: 104-105.
63. Welch SG, Lee J, McGregor IA, Williams K (1978) Red cell glucose 6 phosphate dehydrogenase genotypes of the population of two West African villages. Hum Genet 43: 315-320.
64. Johnson MK, Clark TD, Njama-Meya D, Rosenthal PJ, Parikh S (2009) Impact of the method of G6PD deficiency assessment on genetic association studies of malaria susceptibility. PLoS One 4: e7246.
65. Katrak S, Gasasira A, Arinaitwe E, Kakuru A, Wanzira H, et al. (2009) Safety and tolerability of artemether-lumefantrine versus dihydroartemisinin-piperaquine for malaria in young HIV-infected and uninfected children. Malar J 8: 272.
66. Parikh S, Johnson MK, Kamya MR, Dorsey G, Rosenthal PJ (2008) Glucose 6-Phosphate Dehydrogenase (G6pd) Deficiency Genotype-Phenotype Correlations in Malaria Association Studies. American Journal of Tropical Medicine and Hygiene 79: 243-243.
67. Enevold A, Lusingu JP, Mmbando B, Alifrangis M, Lemnge MM, et al. (2008) Reduced risk of uncomplicated malaria episodes in children with alpha+-thalassemia in northeastern Tanzania. Am J Trop Med Hyg 78: 714-720.
68. Shekalaghe S, Alifrangis M, Mwanziva C, Enevold A, Mwakalinga S, et al. (2009) Low density parasitaemia, red blood cell polymorphisms and Plasmodium falciparum specific immune responses in a low endemic area in northern Tanzania. BMC Infect Dis 9: 69.
69. Roberts DF, Papiha SS (1978) Les polymorphismes genetiques des Sukuma (Tanzania). L'Anthropologie 82: 565-574.
70. Shekalaghe SA, Ter Braak R, Daou M, Kavishe R, van den Bijllaardt W, et al. (2010) Haemolysis after a single dose of primaquine co-administered with an artemisinin is not restricted to glucose-6-phosphate dehydrogenase (G6PD A- variant) deficient individuals in Tanzania. Antimicrob Agents Chemother 54: 1762-1768.
71. Hamel AR, Cabral IR, Sales TS, Costa FF, Olalla Saad ST (2002) Molecular heterogeneity of G6PD deficiency in an Amazonian population and description of four new variants. Blood Cells Mol Dis 28: 399-406.

72. Saad ST, Salles TS, Carvalho MH, Costa FF (1997) Molecular characterization of glucose-6-phosphate dehydrogenase deficiency in Brazil. Hum Hered 47: 17-21.
73. Weimer TA, Salzano FM, Westwood B, Beutler E (1998) G6PD variants in three South American ethnic groups: population distribution and description of two new mutations. Hum Hered 48: 92-96.
74. Azevedo ES, Alves AF, Da Silva MC, Souza MG, Muniz Dias Lima AM, et al. (1980) Distribution of abnormal hemoglobins and glucose-6-phosphate dehydrogenase variants in 1200 school children of Bahia, Brazil. Am J Phys Anthropol 53: 509-512.
75. Weimer TA, Salzano FM, Westwood B, Beutler E (1993) Molecular characterization of glucose-6-phosphate dehydrogenase variants from Brazil. Hum Biol 65: 41-47.
76. Weimer TA, Salzano FM, Hutz MH (1981) Erythrocyte isozymes and hemoglobin types in a southern Brazilian population. Journal of Human Evolution 10: 319-328.
77. Saad ST, Costa FF (1992) Glucose-6-phosphate dehydrogenase deficiency and sickle cell disease in Brazil. Hum Hered 42: 125-128.
78. Neto JPD, Dourado MV, dos Reis MG, Goncalves MS (2008) A novel c. 197T -> A variant among Brazilian neonates with glucose-6-phosphate dehydrogenase deficiency. Genetics and Molecular Biology 31: 33-35.
79. Santana MS, de Lacerda MV, Barbosa MG, Alecrim WD, Alecrim MG (2009) Glucose-6-phosphate dehydrogenase deficiency in an endemic area for malaria in Manaus: a cross-sectional survey in the Brazilian Amazon. PLoS One 4: e5259.
80. de Castro SM, Weber R, Matte U, Giugliani R (2007) Molecular characterization of glucose-6-phosphate dehydrogenase deficiency in patients from the Southern Brazilian city of Porto Alegre, RS. Genetics and Molecular Biology 30: 10-13.
81. Oliveira RAG, Oshiro M, Hirata MH, Hirata RDC, Ribeiro GS, et al. (2009) A novel point mutation in a class IV glucose-6-phosphate dehydrogenase variant (G6PD Sao Paulo) and polymorphic G6PD variants in Sao Paulo State, Brazil. Genetics and Molecular Biology 32: 251-254.
82. Chan TY (1997) Co-trimoxazole-induced severe haemolysis: the experience of a large general hospital in Hong Kong. Pharmacoepidemiol Drug Saf 6: 89-92.
83. Mezzacappa MA, Facchini FP, Pinto AC, Cassone AE, Souza DS, et al. (2010) Clinical and genetic risk factors for moderate hyperbilirubinemia in Brazilian newborn infants. Journal of perinatology : official journal of the California Perinatal Association 30: 819-826.
84. Cardoso MA, Scopel KK, Muniz PT, Villamor E, Ferreira MU (2012) Underlying factors associated with anemia in amazonian children: a population-based, cross-sectional study. PLoS One 7: e36341.
85. Hutz MH, Yoshida A, Salzano FM (1977) Three rare G-6-PD variants from Porto Alegre, Brazil. Human Genetics 39: 191-197.
86. Beutler E, Kuhl W, Saenz GF, Rodriguez W (1991) Mutation analysis of glucose-6-phosphate dehydrogenase (G6PD) variants in Costa Rica. Hum Genet 87: 462-464.
87. Saenz GF, Chaves M, Berrantes A, Elizondo J, Montero AG, et al. (1984) A glucose-6-phosphate dehydrogenase variant, Gd(-) Santamaria found in Costa Rica. Acta Haematol 72: 37-40.
88. Martinez-Labarga C, Rickards O, Scacchi R, Corbo RM, Biondi G, et al. (1999) Genetic population structure of two African-Ecuadorian communities of Esmeraldas. Am J Phys Anthropol 109: 159-174.
89. Vaca G, Arambula E, Esparza A (2002) Molecular heterogeneity of glucose-6-phosphate dehydrogenase deficiency in Mexico: overall results of a 7-year project. Blood Cells Mol Dis 28: 436-444.
90. Vaca G, Arambula E, Monsalvo A, Medina C, Nunez C, et al. (2003) Glucose-6-phosphate dehydrogenase (G-6-PD) mutations in Mexico: four new G-6-PD variants. Blood Cells Mol Dis 31: 112-120.

91. Lisker R, Cordova MS, Graciela Zarate QB (1969) Studies on several genetic hematological traits of the Mexican population. XVI. Hemoglobin, S and glucose-6-phosphate dehydrogenase deficiency in the east coast. Am J Phys Anthropol 30: 349-354.
92. Beutler E, Kuhl W, Ramirez E, Lisker R (1991) Some Mexican glucose-6-phosphate dehydrogenase variants revisited. Hum Genet 86: 371-374.
93. Lisker R, Perez-Briceno R, Rave V, Yoshida A (1981) [Federal District glucose-6-phosphate dehydrogenase Gd(-). A new variant associated with moderate enzyme deficiency and occasional hemolytic anemia]. Rev Invest Clin 33: 209-211.
94. Medina MD, Vaca G, Lopez-Guido B, Westwood B, Beutler E (1997) Molecular genetics of glucose-6-phosphate dehydrogenase deficiency in Mexico. Blood Cells Mol Dis 23: 88-94.
95. Beutler E, Westwood B, Prchal JT, Vaca G, Bartsocas CS, et al. (1992) New glucose-6-phosphate dehydrogenase mutations from various ethnic groups. Blood 80: 255-256.
96. Cossio-Gurrola G, Arambula-Meraz E, Perea M, Garcia N, Correa AS, et al. (2010) Glucose-6-phosphate dehydrogenase (G6PD) molecular variant deficiency: identification in Panama pediatric population. Blood Cells Mol Dis 44: 115-116.
97. Kim S, Nguon C, Guillard B, Duong S, Chy S, et al. (2011) Performance of the CareStart G6PD deficiency screening test, a point-of-care diagnostic for primaquine therapy screening. PloS one 6: e28357.
98. Narazah MY, Devenish R, Rostenberghe HV, Nishiyama K, Shirakawa T, et al. (2004) Molecular basis of glucose-6-phosphate dehydrogenase (G6PD) deficiency in Cambodia. Faops 2004: Proceedings of the 13th Congress of the Federation of Asia and Oceania Perinatal Societies Faops 2004: 149-152

186.
99. Louicharoen C, Nuchprayoon I (2005) G6PD Viangchan (871G>A) is the most common G6PD-deficient variant in the Cambodian population. J Hum Genet 50: 448-452.
100. Viallard JL, Cottreau D, Kahn A, Dastugue B (1979) G6PD deficiency with Gd(-)A like variant in a Chinese family from Cambodia. Hum Genet 51: 213-215.
101. El-Hazmi MA (1987) Haemoglobinopathies, thalassaemias and enzymopathies in Saudi Arabia: the present status. Acta Haematol 78: 130-134.
102. Cai W, Filosa S, Martini G (1994) DNA haplotypes in the G6PD gene cluster studied in the Chinese Li population and their relationship to G6PDCanton. Hum Hered 44: 279-286.
103. Cai W, Filosa S, Martini G, Zhou Y, Zhou D, et al. (2001) [Molecular characterization of glucose-6-phosphate dehydrogenase deficiency in the Han and Li nationalities in Hainan, China and identification of a new mutation in human G6PD gene]. Zhonghua Yi Xue Yi Chuan Xue Za Zhi 18: 105-109.
104. Chiu DTY, Zuo L, Chao L, Chen E, Louie E, et al. (1993) Molecular characterization of glucose-6-phosphate dehydrogenase (G6PD) deficiency in patients of Chinese descent and identification of new base substitutions in the human G6PD gene. Blood 81: 2150-2154.
105. Jiang W, Du C, Duan S, Ma L, Yang L, et al. (1999) [Molecular characterization of glucose-6-phosphate dehydrogenase variants in four ethnic groups in Yunnan province of China]. Zhonghua Yi Xue Yi Chuan Xue Za Zhi 16: 149-152.
106. Jiang W, Yu G, Liu P, Geng Q, Chen L, et al. (2006) Structure and function of glucose-6-phosphate dehydrogenase-deficient variants in Chinese population. Hum Genet 119: 463-478.
107. Wu CX, He Y, Shan KR, Li Y, Xiu J, et al. (2006) Study the mutations of glucose-6-phosphate dehydrogenase gene in Yao ethnic group in Guizhou Libo. Chinese Journal of Endemiology 25: 402-404.
108. Xiu J, Qi XL, Shan KR, Xie Y, He Y, et al. (2005) [G6PD Gene Mutations in Shui people in Sandu of Guizhou]. Zhongguo Shi Yan Xue Ye Xue Za Zhi 13: 147-150.
109. Yan T, Cai R, Mo O, Zhu D, Ouyang H, et al. (2006) Incidence and complete molecular characterization of glucose-6-phosphate dehydrogenase deficiency in the Guangxi Zhuang

autonomous region of southern China: description of four novel mutations. Haematologica 91: 1321-1328.
110. Zhang DT, Hu LH, Yang YZ (2005) Detection of three common G6PD gene mutations in Chinese individuals by probe melting curves. Clin Biochem 38: 390-394.
111. Chan TK (1983) Glucose 6 phosphate dehydrogenase (G6PD) [MD]: University of Hong Kong.
112. Au WY, Ma ES, Lam VM, Chan JL, Pang A, et al. (2004) Glucose 6-phosphate dehydrogenase (G6PD) deficiency in elderly Chinese women heterozygous for G6PD variants. Am J Med Genet A 129A: 208-211.
113. Bang-Ce Y, Hongqiong L, Zhensong L (2004) Rapid detection of common Chinese glucose-6-phosphate dehydrogenase (G6PD) mutations by microarray-based assay. Am J Hematol 76: 405-412.
114. Chan TK, Todd D, Lai MC (1972) Glucose 6-phosphate dehydrogenase: identity of erythrocyte and leukocyte enzyme with report of a new variant in Chinese. Biochem Genet 6: 119-124.
115. Deng C, Guo CB, Xu YH, Deng B, Yu JL (2007) Three mutations analysis of glucose-6-phosphate dehydrogenase deficiency in neonates in South-west China. Pediatr Int 49: 463-467.
116. Du CS, Ren X, Chen L, Jiang W, He Y, et al. (1999) Detection of the most common G6PD gene mutations in Chinese using amplification refractory mutation system. Hum Hered 49: 133-138.
117. Du CS, Xu YK, Hua XY, Wu QL, Liu LB (1988) Glucose-6-phosphate dehydrogenase variants and their frequency in Guangdong, China. Hum Genet 80: 385-388.
118. Li L, Zhou YQ, Xiao QZ, Yan TZ, Xu XM (2008) Development and evaluation of a reverse dot blot assay for the simultaneous detection of six common Chinese G6PD mutations and one polymorphism. Blood Cells Mol Dis 41: 17-21.
119. Li P, Thompson JN, Wang X, Song L (1998) Analysis of common mutations and associated haplotypes in Chinese patients with glucose-6-phosphate dehydrogenase deficiency. Biochem Mol Biol Int 46: 1135-1143.
120. McCurdy PR, Kirkman HN, Naiman JL, Jim RT, Pickard BM (1966) A Chinese variant of glucose-6-phosphate dehydrogenase. J Lab Clin Med 67: 374-385.
121. Xu W, Westwood B, Bartsocas CS, Malcorra-Azpiazu JJ, Indrak K, et al. (1995) Glucose-6 phosphate dehydrogenase mutations and haplotypes in various ethnic groups. Blood 85: 257-263.
122. Wang YF, Xia WQ, Ni PH, Hu YQ, Jiang XC (2010) Analysis of glucose-6-phosphate dehydrogenase gene mutations: A novel missense mutation. Journal of Shanghai Jiaotong University (Medical Science) 30: 698-702.
123. Jiang WY, Zhou BY, Yu GL, Liu H, Zeng JB, et al. (2012) G6PD genotype and its associated enzymatic activity in a Chinese population. Biochemical genetics 50: 34-44.
124. Yan JB, Xu HP, Xiong C, Ren ZR, Tian GL, et al. (2010) Rapid and reliable detection of glucose-6-phosphate dehydrogenase (G6PD) gene mutations in Han Chinese using high-resolution melting analysis. The Journal of molecular diagnostics : JMD 12: 305-311.
125. Huang YY, Huang CS, Yang SS, Lin MS, Huang MJ, et al. (2005) Effects of variant UDP-glucuronosyltransferase 1A1 gene, glucose-6-phosphate dehydrogenase deficiency and thalassemia on cholelithiasis. World J Gastroenterol 11: 5710-5713.
126. Tseng CP, Huang CL, Chong KY, Hung IJ, Chiu DT (2005) Rapid detection of glucose-6-phosphate dehydrogenase gene mutations by denaturing high-performance liquid chromatography. Clin Biochem 38: 973-980.
127. Tang TK, Liu TH, Tang CJ, Tam KB (1995) Glucose-6-phosphate dehydrogenase (G6PD) mutations associated with F8C/G6PD haplotypes in Chinese. Blood 85: 3767-3768.
128. Chan TK, Todd D (1972) Characteristics and distribution of glucose-6-phosphate dehydrogenase-deficient variants in South China. Am J Hum Genet 24: 475-484.

129. Chiang SH, Wu SJ, Wu KF, Hsiao KJ (1999) Neonatal screening for glucose-6-phosphate dehydrogenase deficiency in Taiwan. Southeast Asian J Trop Med Public Health 30 Suppl 2: 72-74.
130. Tang TK, Huang WY, Tang CJ, Hsu M, Cheng TA, et al. (1995) Molecular basis of glucose-6-phosphate dehydrogenase (G6PD) deficiency in three Taiwan aboriginal tribes. Hum Genet 95: 630-632.
131. Wan GH, Lin KK, Tsai SC, Chiu DT (2006) Decreased glucose-6-phosphate-dehydrogenase (G6PD) activity and risk of senile cataract in Taiwan. Ophthalmic Epidemiol 13: 109-114.
132. Wan GH, Tsai SC, Chiu DT (2002) Decreased blood activity of glucose-6-phosphate dehydrogenase associates with increased risk for diabetes mellitus. Endocrine 19: 191-195.
133. Huang CS, Hung KL, Huang MJ, Li YC, Liu TH, et al. (1996) Neonatal jaundice and molecular mutations in glucose-6-phosphate dehydrogenase deficient newborn infants. Am J Hematol 51: 19-25.
134. Ko CH, Yung E, Li K, Li CL, Ng PC, et al. (2006) Multiplex primer extension reaction screening and oxidative challenge of glucose-6-phosphate dehydrogenase mutants in hemizygous and heterozygous subjects. Blood Cells Mol Dis 37: 21-26.
135. Chang JG, Chiou SS, Perng LI, Chen TC, Liu TC, et al. (1992) Molecular characterization of glucose-6-phosphate dehydrogenase (G6PD) deficiency by natural and amplification created restriction sites: five mutations account for most G6PD deficiency cases in Taiwan. Blood 80: 1079-1082.
136. Tang TK, Yeh CH, Huang CS, Huang MJ (1994) Expression and biochemical characterization of human glucose-6-phosphate dehydrogenase in Escherichia coli: a system to analyze normal and mutant enzymes. Blood 83: 1436-1441.
137. Chen HL, Huang MJ, Huang CS, Tang TK (1997) Two novel glucose 6-phosphate dehydrogenase deficiency mutations and association of such mutations with F8C/G6PD haplotype in Chinese. J Formos Med Assoc 96: 948-954.
138. Qi XL, Shan K, Xie Y, Wu CX, Xiu J, et al. (2006) Study on the mutations of G6PD gene in Dong ethnic group in Guizhou Congjiang. Chinese Journal of Endemiology 25: 283-285.
139. Du C, He Y (1997) [A case of nt 1004C --> A G6PD gene mutation in Yunnan Han people]. Zhonghua Xue Ye Xue Za Zhi 18: 535-537.
140. Ren X, He Y, Du C, Jiang W, Chen L, et al. (2001) A novel mis-sense mutation (G1381A) in the G6PD gene identified in a Chinese man. Chin Med J (Engl) 114: 399-401.
141. Wu CX, Shan KR, He Y, Qi XL, Li Y, et al. (2007) Detection of glucose-6-phosphate dehydrogenase gene mutations of Tujia ehtnic in Jiangkou, Guizhou. Chinese Journal of Endemiology 26: 415-417.
142. Wang J, Matsuoka H, Hirai M, Mu L, Yang L, et al. (2010) The First Case of a Class I Glucose-6-phosphate Dehydrogenase Deficiency, G6PD Santiago de Cuba (1339 GA), in a Chinese Population as Found in a Survey for G6PD Deficiency in Northeastern and Central China. Acta Med Okayama 64: 49-54.
143. Xu W, Wang J, Hua X, Du C (1994) Detection of point mutations in exon 2 of the G6PD gene in Chinese G6PD variants. Chin Med Sci J 9: 20-23.
144. Chao LT, Du CS, Louie E, Zuo L, Chen E, et al. (1991) A to G substitution identified in exon 2 of the G6PD gene among G6PD deficient Chinese. Nucleic Acids Res 19: 6056.
145. Chen HL, Huang MJ, Huang CS, Tang TK (1996) G6PD NanKang (517 T-->C; 173 Phe-->Leu): a new Chinese G6PD variant associated with neonatal jaundice. Hum Hered 46: 201-204.
146. Tang TK, Chen HL, Huang CS, Liu TH (1995) Identification of a novel G6PD mutation (G6PD NanKang) and the association of F8C/G6PD haplotypes in Chinese. Blood 86: 525-525.
147. Yang Y, Zhu Y, Li D, Li Z, Lu H, et al. (2007) Characterization of glucose-6-phosphate dehydrogenase deficiency and identification of a novel haplotype 487G>A/IVS5-612(G>C) in the Achang population of Southwestern China. Sci China C Life Sci 50: 479-485.

148. Du CS, Liu LB, Liu B, Tokunaga K, Omoto K (1988) Glucose-6-phosphate dehydrogenase deficiency among three national minorities in Hainan Island, China. Gene Geogr 2: 71-74.
149. Sukumar S, Mukherjee MB, Colah RB, Mohanty D (2004) Molecular basis of G6PD deficiency in India. Blood Cells Mol Dis 33: 141-145.
150. Chalvam R, Mukherjee MB, Colah RB, Mohanty D, Ghosh K (2007) G6PD Namoru (208 T--> C) is the major polymorphic variant in the tribal populations in southern India. Br J Haematol 136: 512-513.
151. Chalvam R, Kedar PS, Colah RB, Ghosh K, Mukherjee MB (2008) A novel R198H mutation in the glucose-6-phosphate dehydrogenase gene in the tribal groups of the Nilgiris in Southern India. J Hum Genet 53: 181-184.
152. Chalvam R, Colah RB, Mohanty D, Ghosh K, Mukherjee MB (2009) Molecular heterogeneity of glucose-6-phosphate dehydrogenase deficiency among the tribals in Western India. Blood Cells Mol Dis 43: 156-157.
153. Sarkar S, Biswas NK, Dey B, Mukhopadhyay D, Majumder PP (2010) A large, systematic molecular-genetic study of G6PD in Indian populations identifies a new non-synonymous variant and supports recent positive selection. Infection, genetics and evolution : journal of molecular epidemiology and evolutionary genetics in infectious diseases 10: 1228-1236.
154. Nishank SS, Chhotray GP, Kar SK, Ranjit MR (2008) Molecular variants of G6PD deficiency among certain tribal communities of Orissa, India. Ann Hum Biol 35: 355-361.
155. Beutler E, Kuhl W (1990) The NT 1311 polymorphism of G6PD: G6PD Mediterranean mutation may have originated independently in Europe and Asia. Am J Hum Genet 47: 1008-1012.
156. Sukumar S, Colah R, Mohanty D (2002) G6PD gene mutations in India producing drug-induced haemolytic anaemia. Br J Haematol 116: 671-672.
157. Ishwad CS, Naik SN (1984) A new glucose-6-phosphate dehydrogenase variant (G-6-PD Kalyan) found in a Koli family. Hum Genet 66: 171-175.
158. Sayyed Z, Mukherjee MB, Mudera VC, Colah R, Gupte S (1992) Characterization of G6PD Rohini--a new class III Indian variant. Indian J Med Res 96: 96-100.
159. Sukumar S, Mukherjee MB, Colah RB, Mohanty D (2005) Two distinct Indian G6PD variants G6PD Jamnagar and G6PD Rohini caused by the same 949 G-->A mutation. Blood cells, molecules & diseases 35: 193-195.
160. Murhekar KM, Murhekar MV, Mukherjee MB, Gorakshakar AC, Surve R, et al. (2001) Red cell genetic abnormalities, beta-globin gene haplotypes, and APOB polymorphism in the Great Andamanese, a primitive Negrito tribe of Andaman and Nicobar Islands, India. Hum Biol 73: 739-744.
161. Kaeda JS, Chhotray GP, Ranjit MR, Bautista JM, Reddy PH, et al. (1995) A new glucose-6-phosphate dehydrogenase variant, G6PD Orissa (44 Ala-->Gly), is the major polymorphic variant in tribal populations in India. Am J Hum Genet 57: 1335-1341.
162. Iwai K, Hirono A, Matsuoka H, Kawamoto F, Horie T, et al. (2001) Distribution of glucose-6-phosphate dehydrogenase mutations in Southeast Asia. Hum Genet 108: 445-449.
163. Soemantri AG, Saha S, Saha N, Tay JS (1995) Molecular variants of red cell glucose-6-phosphate dehydrogenase deficiency in Central Java, Indonesia. Hum Hered 45: 346-350.
164. Yuniar I (2006) Prevalensi varian Canton dan Kaiping pada defisiensi enzim glukosa-6-fosfat dehidrogenase yang mengalami ikterus pada masa neonatus. Jakarta: Universitas Indonesia.
165. Suhartati (2007) Pola mutan gen glukosa 6 fosfat dehidrogenase di Surabaya. Majalah Ilmu Faal Indonesia: 130-139.
166. Suhartati (2007) Mutasi gen penyebab defisiensi glukosa 6 fosfat dehidrogenase (G6PD) di Surabaya dan Kepulauan Maluku Tenggara : Tinjauan biologi molekuler dan genetika populasi terhadap G6PD. Surabaya: Universitas Airlangga.
167. Francine MR (2000) Molecular analysis for the detection of glucose-6-phosphate dehydrogenase (G6PD) deficiency. Yogyakarta: Gadjah Mada University.

168. Kawamoto F, Matsuoka H, Kanbe T, Tantular IS, Pusarawati S, et al. (2006) Further investigations of glucose-6-phosphate dehydrogenase variants in Flores Island, eastern Indonesia. J Hum Genet 51: 952-957.
169. Matsuoka H, Arai M, Yoshida S, Tantular IS, Pusarawati S, et al. (2003) Five different glucose-6-phophate [correction phosphate]dehydrogenase (G6PD) variants found among 11 G6PD-deficient persons in Flores Island, Indonesia. J Hum Genet 48: 541-544.
170. Soewono S, Martini T, Shirakawa T, Nishiyama K (2000) Glucose-6-phosphate dehydrogenase (G6PD) deficiency variants in small isolated islands in eastern Indonesia. Jurnal Kedokteran Yarsi 8: 87-92.
171. Tantular IS, Matsuoka H, Kasahara Y, Pusarawati S, Kanbe T, et al. (2010) Incidence and mutation analysis of glucose-6-phosphate dehydrogenase deficiency in eastern Indonesian populations. Acta medica Okayama 64: 367-373.
172. Suhartati, Martini T, Soewono I, Shirakawa T, Nishiyama K (2002) Molecular study in G6PD deficiency, a pedigree analysis of a Javanese-Chinese family in Surabaya, Indonesia. Jurnal Kedokteran Yarsi 10: 30-34.
173. Kurniatun Y (2000) Deteksi defisiensi dehidrogenase glukosa-6-fosfat (G6PD) pada daerah endemik malaria dengan pendekatan molekuler. Yogyakarta: Universitas Gadjah Mada.
174. Francine MR, Sofro ASM, Artama WT (2001) Molecular analysis for the detection of glucose 6-phosphate dehydrogenase (G6PD) deficiency. Teknosains 14: 169-178.
175. Karimi M, Martinez di Montemuros F, Danielli MG, Farjadian S, Afrasiabi A, et al. (2003) Molecular characterization of glucose-6-phosphate dehydrogenase deficiency in the Fars province of Iran. Haematologica 88: 346-347.
176. Karimi M, Yavarian M, Afrasiabi A, Dehbozorgian J, Rachmilewitz E (2008) Prevalence of beta-thalassemia trait and glucose-6-phosphate dehydrogenase deficiency in Iranian Jews. Arch Med Res 39: 212-214.
177. Rahimi Z, Vaisi-Raygani A, Nagel RL, Muniz A (2006) Molecular characterization of glucose-6-phosphate dehydrogenase deficiency in the Kurdish population of Western Iran. Blood Cells Mol Dis 37: 91-94.
178. Mesbah-Namin SA, Sanati MH, Mowjoodi A, Mason PJ, Vulliamy TJ, et al. (2002) Three major glucose-6-phosphate dehydrogenase-deficient polymorphic variants identified in Mazandaran state of Iran. Br J Haematol 117: 763-764.
179. Noori-Daloii MR, Hajebrahimi Z, Najafi L, Mesbah-Namin SA, Mowjoodi A, et al. (2007) A comprehensive study on the major mutations in glucose-6-phosphate dehydrogenase-deficient polymorphic variants identified in the coastal provinces of Caspian Sea in the north of Iran. Clin Biochem 40: 699-704.
180. Noori-Daloii MR, Najafi L, Mohammad Ganji S, Hajebrahimi Z, Sanati MH (2004) Molecular identification of mutations in G6PD gene in patients with favism in Iran. J Physiol Biochem 60: 273-277.
181. Nezhad SRK, Fahmi F, Khatami SR, Musaviun M (2011) Molecular characterization of cosenza mutation among patients with glucose-6-phosphate dehydrogenase deficiency in Khuzestan province, southwest Iran. Iranian Journal of Medical Sciences 36: 40-44.
182. Gandomani MG, Khatami SR, Nezhad SR, Daneshmand S, Mashayekhi A (2011) Molecular identification of G6PD Chatham (G1003A) in Khuzestan province of Iran. Journal of genetics 90: 143-145.
183. Mortazavi YM, Soleimani MS, Lahijani ANA, Omidkhoda AO, Ghavamzadeh A (2008) The Frequency and Molecular Genetics of G6pd Deficiency in Northwest and Southeast of Iran. Haematologica-the Hematology Journal 93: 485-486.
184. Nezhad SRK, Mashayekhi A, Khatami SR, Daneshmand S, Fahmi F, et al. (2009) Prevalence and Molecular Identification of Mediterranean Glucose-6-Phosphate Dehydrogenase Deficiency in Khuzestan Province, Iran. Iranian Journal of Public Health 38: 127-131.

185. Mortazavi Y, Mirzamohammadi F, Ardestani MT, Mirimoghadam E, Vulliamy TJ (2010) Glucos 6-phosphate dehydrogenase deficiency in Tehran, Zanjan and sistan-balouchestan provinces: Prevalence and frequency of Mediterranean variant of G6PD. Iranian Journal of Biotechnology 8: 229-233.
186. Nakhaee A, Salimi S, Zadehvakili A, Dabiri S, Noora M, et al. (2012) The Prevalence of Mediterranean Mutation of Glucose-6-Phosphate Dehydrogenase (G6PD) in Zahedan. Zahedan Journal of Research in Medical Sciences 14: 39-43.
187. Al-Allawi N, Eissa AA, Jubrael JM, Jamal SA, Hamamy H (2010) Prevalence and molecular characterization of Glucose-6-Phosphate dehydrogenase deficient variants among the Kurdish population of Northern Iraq. BMC Blood Disord 10: 6.
188. Al-Musawi BM, Al-Allawi N, Abdul-Majeed BA, Eissa AA, Jubrael JM, et al. (2012) Molecular characterization of glucose-6-phosphate dehydrogenase deficient variants in Baghdad city - Iraq. BMC blood disorders 12: 4.
189. Hilmi FA, Al-Allawi NA, Rassam M, Al-Shamma G, Al-Hashimi A (2002) Red cell glucose-6-phosphate dehydrogenase phenotypes in Iraq. East Mediterr Health J 8: 42-48.
190. Benbassat J, Ben-Ishay D (1969) Hereditary hemolytic anemia associated with glucose-6-phosphate dehydrogenase deficiency (Mediterranean type). Isr J Med Sci 5: 1053-1059.
191. Kurdi-Haidar B, Mason PJ, Berrebi A, Ankra-Badu G, al-Ali A, et al. (1990) Origin and spread of the glucose-6-phosphate dehydrogenase variant (G6PD-Mediterranean) in the Middle East. Am J Hum Genet 47: 1013-1019.
192. Ainoon O, Yu YH, Amir Muhriz AL, Boo NY, Cheong SK, et al. (2002) Glucose-6-phosphate dehydrogenase (G6PD) variants in Malaysian Malays. Hum Mutat 21: 101.
193. Ainoon O, Joyce J, Boo NY, Cheong SK, Hamidah NH (1995) Nucleotide 1376 G-->T mutation in G6PD-deficient Chinese in Malaysia. Malays J Pathol 17: 61-65.
194. Othman A, Wong F, Boo N, Wang M, Nh H (2008) Rapid molecular screening of G6PD variants in Malaysian Chinese newborns using Taqman MGB SNP assay. International Journal of Laboratory Hematology. pp. (Suppl 1) 125-125.
195. Boo NY, Wong FL, Wang MK, Othman A (2009) Homozygous variant of UGT1A1 gene mutation and severe neonatal hyperbilirubinemia. Pediatr Int 51: 488-493.
196. Ainoon O, Boo NY, Yu YH, Cheong SK, Hamidah HN, et al. (2004) Complete molecular characterisation of glucose-6-phosphate dehydrogenase (G6PD) deficiency in a group of Malaysian Chinese neonates. Malays J Pathol 26: 89-98.
197. Ainoon O, Joyce J, Boo NY, Cheong SK, Zainal ZA, et al. (1999) Glucose-6-phosphate dehydrogenase (G6PD) variants in Malaysian Chinese. Hum Mutat 14: 352.
198. Wang J, Luo E, Hirai M, Arai M, Abdul-Manan E, et al. (2008) Nine different glucose-6-phosphate dehydrogenase (G6PD) variants in a Malaysian population with Malay, Chinese, Indian and Orang Asli (aboriginal Malaysian) backgrounds. Acta Med Okayama 62: 327-332.
199. Yusoff NM, Shirakawa T, Nishiyama K, Ghazali S, Ee CK, et al. (2002) Molecular heterogeneity of glucose-6-phosphate dehydrogenase deficiency in Malays in Malaysia. Int J Hematol 76: 149-152.
200. Nadarajan V, Shanmugam H, Sthaneshwar P, Jayaranee S, Sultan KS, et al. (2011) Modification to reporting of qualitative fluorescent spot test results improves detection of glucose-6-phosphate dehydrogenase (G6PD)-deficient heterozygote female newborns. International journal of laboratory hematology 33: 463-470.
201. Matsuoka H, Wang J, Hirai M, Arai M, Yoshida S, et al. (2004) Glucose-6-phosphate dehydrogenase (G6PD) mutations in Myanmar: G6PD Mahidol (487G>A) is the most common variant in the Myanmar population. J Hum Genet 49: 544-547.
202. Nuchprayoon I, Louicharoen C, Charoenvej W (2008) Glucose-6-phosphate dehydrogenase mutations in Mon and Burmese of southern Myanmar. J Hum Genet 53: 48-54.

203. Than AM, Harano T, Harano K, Myint AA, Ogino T, et al. (2005) High incidence of 3-thalassemia, hemoglobin E, and glucose-6-phosphate dehydrogenase deficiency in populations of malaria-endemic southern Shan State, Myanmar. Int J Hematol 82: 119-123.
204. Matsuoka H, Jichun W, Hirai M, Yoshida S, Arai M, et al. (2003) Two cases of glucose-6-phosphate dehydrogenase-deficient Nepalese belonging to the G6PD Mediterranean-type, not India-Pakistan sub-type but Mediterranean-Middle East sub-type. J Hum Genet 48: 275-277.
205. Moiz B, Nasir A, Moatter T, Naqvi ZA, Khurshid M (2011) Molecular characterization of glucose-6-phosphate dehydrogenase deficiency in Pakistani population. International journal of laboratory hematology 33: 570-578.
206. Saha N, Ramzan M, Tay JS, Low PS, Basair JB, et al. (1994) Molecular characterisation of red cell glucose-6-phosphate dehydrogenase deficiency in north-west Pakistan. Hum Hered 44: 85-89.
207. Moiz B, Nasir A, Moatter T, Naqvi ZA, Khurshid M (2009) Population study of 1311 C/T polymorphism of Glucose 6 Phosphate Dehydrogenase gene in Pakistan - an analysis of 715 X-chromosomes. BMC Genet 10: 41.
208. Leslie T, Briceno M, Mayan I, Mohammed N, Klinkenberg E, et al. (2010) The impact of phenotypic and genotypic G6PD deficiency on risk of plasmodium vivax infection: a case-control study amongst Afghan refugees in Pakistan. PLoS medicine 7: e1000283.
209. Hung NM, Eto H, Mita T, Tsukahara T, Hombhanje FW, et al. (2008) Glucose - 6 - Phosphate Dehydrogenase (G6PD) variants in East Sepik Province of Papua New Guinea : G6PD Jammu, G6PD Vanua Lava, and a novel variant (G6PD Dagua). Tropical Medicine and Health 36: 163-169.
210. Wagner G, Bhatia K, Board P (1996) Glucose-6-phosphate dehydrogenase deficiency mutations in Papua New Guinea. Hum Biol 68: 383-394.
211. Yoshida A, Giblett ER, Malcolm LA (1973) Heterogeneous distribution of glucose-6-phosphate dehydrogenase variants with enzyme deficiency in the Markham Valley Area of New Guinea. Ann Hum Genet 37: 145-150.
212. Chockkalingam K, Board PG, Nurse GT (1982) Glucose-6-phosphate dehydrogenase deficiency in Papua New Guinea. The description of 13 new variants. Hum Genet 60: 189-192.
213. Silao CL, Shirakawa T, Nishiyama K, Padilla C, Matsuo M (1999) Molecular basis of glucose-6-phosphate dehydrogenase deficiency among Filipinos. Pediatr Int 41: 138-141.
214. Yoshida A, Baur EW, Moutlsky AG (1970) A Philippino glucose-6-phosphate dehydrogenase variant (G6PD Union) with enzyme deficiency and altered substrate specificity. Blood 35: 506-513.
215. Niazi GA, Adeyokunnu A, Westwood B, Beutler E (1996) Neonatal jaundice in Saudi newborns with G6PD Aures. Ann Trop Paediatr 16: 33-37.
216. Mohamed MM, El-Humiany AUR (2006) Molecular characterization of new variants of glucose-6-phosphate dehydrogenase deficiency gene isolated in Western province of Saudi Arabia causing hemolytic anemia. Pakistan Journal of Biological Sciences 9: 1605-1616.
217. Al-Jaouni SK, Jarullah J, Azhar E, Moradkhani K (2011) Molecular characterization of glucose-6-phosphate dehydrogenase deficiency in Jeddah, Kingdom of Saudi Arabia. BMC research notes 4: 436.
218. Faiyaz-Ul-Haque M, Zaidi SH, Hasanato RM, Al-Abdullatif A, Cluntun A, et al. (2010) Genetics of glucose-6-phosphate dehydrogenase deficiency in Saudi patients. Clinical genetics 78: 98-100.
219. Al-Ali AK (1996) Common G6PD variant from Saudi population and its prevalence. Ann Saudi Med 16: 654-656.
220. El-Hazmi MA, Warsy AS (1990) Frequency of glucose-6-phosphate dehydrogenase variants and deficiency in Arabia. Gene Geogr 4: 15-19.

221. El-Hazmi MA, Warsy AS (1991) Glucose-6-phosphate dehydrogenase variants and sickle cell genes in Al-Qunfuda, Saudi Arabia. Trop Geogr Med 43: 174-179.
222. El-Hazmi MA, Warsy AS (1992) The frequency of glucose-6-phosphate dehydrogenase phenotypes and sickle cell gene in Al-Qassim. Ann Saudi Med 12: 463-467.
223. El-Hazmi MA, Warsy AS (1994) The frequency of glucose-6-phosphate dehydrogenase phenotypes and sickle cell genes in Al-Qatif oasis. Ann Saudi Med 14: 491-494.
224. El-Hazmi MA, Warsy AS, Bahakim HH, Al-Swailem A (1994) Glucose-6-phosphate dehydrogenase deficiency and the sickle cell gene in Makkah, Saudi Arabia. J Trop Pediatr 40: 12-16.
225. El-Hazmi MA, Warsy AS, Bahakim HM, Al-Swailem A (1993) Glucose-6-phosphate dehydrogenase deficiency and sickle cell genes in two regions of western Saudi Arabia. Ann Saudi Med 13: 250-254.
226. El-Hazmi MAF, Warsy AS (1986) Glucose-6-phosphate dehydrogenase polymorphism in the Saudi population. Hum Hered 36: 24-30.
227. El-Hazmi MAF, Warsy AS (1993) The Frequency of Hbs and Glucose-6-Phosphate-Dehydrogenase Phenotypes in Relation to Malaria in Western Saudi-Arabia. Saudi Medical Journal 14: 121-125.
228. Gelpi AP, King MC (1977) New data on glucose-6-phosphate dehydrogenase deficiency in Saudi Arabia. G6PD variants, and the association between enzyme deficiency and hemoglobins S. Hum Hered 27: 285-291.
229. Hellani A, Al-Akoum S, Abu-Amero KK (2009) G6PD Mediterranean S188F codon mutation is common among Saudi sickle cell patients and increases the risk of stroke. Genet Test Mol Biomarkers 13: 449-452.
230. Gari MA, Chaudhary AG, Al-Qahtani MH, Abuzenadah AM, Waseem A, et al. (2010) Frequency of Mediterranean mutation among a group of Saudi G6PD patients in Western region-Jeddah. International journal of laboratory hematology 32: 17-21.
231. Samuel AP, Saha N (1986) Distribution of red cell G6PD and 6PGD phenotypes in Saudi Arabia. Trop Geogr Med 38: 287-291.
232. Hirono A, Ishii A, Kere N, Fujii H, Hirono K, et al. (1995) Molecular analysis of glucose-6-phosphate dehydrogenase variants in the Solomon Islands. Am J Hum Genet 56: 1243-1245.
233. Laosombat V, Sattayasevana B, Janejindamai W, Viprakasit V, Shirakawa T, et al. (2005) Molecular heterogeneity of glucose-6-phosphate dehydrogenase (G6PD) variants in the south of Thailand and identification of a novel variant (G6PD Songklanagarind). Blood Cells Mol Dis 34: 191-196.
234. Nuchprayoon I, Sanpavat S, Nuchprayoon S (2002) Glucose-6-phosphate dehydrogenase (G6PD) mutations in Thailand: G6PD Viangchan (871G>A) is the most common deficiency variant in the Thai population. Hum Mutat 19: 185.
235. Panich V (1973) G6pd-Characterization in Thailand. Genetics 74: S208-S208.
236. Panich V, Sungnate T (1973) Characterization of glucose-6-phosphate dehydrogenase in Thailand. The occurrence of 6 variants among 50 G-6-PD deficient Thai. Humangenetik 18: 39-46.
237. Phompradit P, Kuesap J, Chaijaroenkul W, Rueangweerayut R, Hongkaew Y, et al. (2011) Prevalence and distribution of glucose-6-phosphate dehydrogenase (G6PD) variants in Thai and Burmese populations in malaria endemic areas of Thailand. Malar J 10: 368.
238. Ninokata A, Kimura R, Samakkarn U, Settheetham-Ishida W, Ishida T (2006) Coexistence of five G6PD variants indicates ethnic complexity of Phuket islanders, Southern Thailand. J Hum Genet 51: 424-428.
239. Laosombat V, Sattayasevana B, Chotsampancharoen T, Wongchanchailert M (2006) Glucose-6-phosphate dehydrogenase variants associated with favism in Thai children. Int J Hematol 83: 139-143.
240. Tanphaichitr VS, Hirono A, Pung-amritt P, Treesucon A, Wanachiwanawin W (2011) Chronic nonspherocytic hemolytic anemia due to glucose-6-phosphate dehydrogenase deficiency:

report of two families with novel mutations causing G6PD Bangkok and G6PD Bangkok Noi. Annals of hematology 90: 769-775.
241. Panich V, Sungnate T, Wasi P, Na-Nakorn S (1972) G-6-PD Mahidol. The most common glucose-6-phosphate dehydrogenase variant in Thailand. J Med Assoc Thai 55: 576-585.
242. Louicharoen C, Patin E, Paul R, Nuchprayoon I, Witoonpanich B, et al. (2009) Positively selected G6PD-Mahidol mutation reduces *Plasmodium vivax* density in Southeast Asians. Science 326: 1546-1549.
243. Oner R, Gumruk F, Acar C, Oner C, Gurgey A, et al. (2000) Molecular characterization of glucose-6-phosphate dehydrogenase deficiency in Turkey. Haematologica 85: 320-321.
244. Cappellini MD, di Montemuros FM, Prandoni S, Tavazzi D, Iolascon A, et al. (2001) Molecular characterization of G6PD deficiency in subjects negative for common Mediterranean mutations. Blood 98: 13a-13a.
245. Canatan D, Bagci H, Gumuslu S, Bilmen S, Acikbas I, et al. (2006) The features of patients with favism in Turkey. HAEMA 9: 247-250.
246. Keskin N, Ozdes I, Keskin A, Acikbas I, Bagci H (2002) Incidence and molecular analysis of glucose-6-phosphate dehydrogenase deficiency in the province of Denizli, Turkey. Med Sci Monit 8: CR453-456.
247. Yildiz SM, Ariyurek SY, Aksoy K (2010) Detection of Mediterranean Mutation in the Glucose-6-Phosphate Dehydrogenase Gene with Microarray Technique. Turkish Journal of Biochemistry-Turk Biyokimya Dergisi 35: 63-66.
248. Ganczakowski M, Town M, Bowden DK, Vulliamy TJ, Kaneko A, et al. (1995) Multiple glucose 6-phosphate dehydrogenase-deficient variants correlate with malaria endemicity in the Vanuatu archipelago (southwestern Pacific). Am J Hum Genet 56: 294-301.
249. Matsuoka H, Thuan DT, van Thien H, Kanbe T, Jalloh A, et al. (2007) Seven different glucose-6-phosphate dehydrogenase variants including a new variant distributed in Lam Dong Province in southern Vietnam. Acta Med Okayama 61: 213-219.
250. Panich V, Bumrungtrakul P, Jitjai C, Kamolmatayakul S, Khoprasert B, et al. (1980) Glucose-6-phosphate dehydrogenase deficiency in South Vietnamese. Hum Hered 30: 361-364.
251. Toncheva D (1986) Variants of glucose-6-phosphate dehydrogenase in a Vietnamese population. Hum Hered 36: 348-351.
252. Hue NT, Charlieu JP, Chau TT, Day N, Farrar JJ, et al. (2009) Glucose-6-phosphate dehydrogenase (G6PD) mutations and haemoglobinuria syndrome in the Vietnamese population. Malar J 8: 152.
